# Active learning with human heuristics: an algorithm robust to labeling bias

**DOI:** 10.3389/frai.2024.1491932

**Published:** 2024-11-19

**Authors:** Sriram Ravichandran, Nandan Sudarsanam, Balaraman Ravindran, Konstantinos V. Katsikopoulos

**Affiliations:** ^1^Department of Management Studies, Indian Institute of Technology Madras, Chennai, Tamil Nadu, India; ^2^Department of Data Science and AI, Indian Institute of Technology Madras, Chennai, Tamil Nadu, India; ^3^Wadhwani School of Data Science and AI, Indian Institute of Technology Madras, Chennai, Tamil Nadu, India; ^4^Department of Decision Analytics and Risk, University of Southampton Business School, Southampton, United Kingdom

**Keywords:** active learning, human in the loop, human behavior, biases, robustness, fast-and-frugal heuristics

## Abstract

Active learning enables prediction models to achieve better performance faster by adaptively querying an oracle for the labels of data points. Sometimes the oracle is a human, for example when a medical diagnosis is provided by a doctor. According to the behavioral sciences, people, because they employ heuristics, might sometimes exhibit biases in labeling. How does modeling the oracle as a human heuristic affect the performance of active learning algorithms? If there is a drop in performance, can one design active learning algorithms robust to labeling bias? The present article provides answers. We investigate two established human heuristics (fast-and-frugal tree, tallying model) combined with four active learning algorithms (entropy sampling, multi-view learning, conventional information density, and, our proposal, inverse information density) and three standard classifiers (logistic regression, random forests, support vector machines), and apply their combinations to 15 datasets where people routinely provide labels, such as health and other domains like marketing and transportation. There are two main results. First, we show that if a heuristic provides labels, the performance of active learning algorithms significantly drops, sometimes below random. Hence, it is key to design active learning algorithms that are robust to labeling bias. Our second contribution is to provide such a robust algorithm. The proposed inverse information density algorithm, which is inspired by human psychology, achieves an overall improvement of 87% over the best of the other algorithms. In conclusion, designing and benchmarking active learning algorithms can benefit from incorporating the modeling of human heuristics.

## 1 Introduction: active learning with human heuristics

Building prediction models is crucial for automating management decision processes because it enables organizations to make informed decisions based on data rather than relying solely on intuition or past experiences. There is an increasing need for training such models in conditions where obtaining labels is significantly more expensive than their attributes. For example, the safety of automobile designs is assessed by crash tests under carefully controlled conditions which is expensive. An active learning algorithm selects efficiently data points for training prediction models. This selection is made by adaptively querying an oracle for the labels of data points. That is, the training process starts from a small number of labeled data points and queries the oracle for further labels, wherein each query is a function of previously provided labels. Thus, prediction models can achieve better performance faster by employing *active learning* modules (Settles, 2009; Monarch, 2021).

Crucially, the oracle providing the labels is typically assumed to be unbiased (Wu et al., 2012; Cohn et al., 1994; Lan et al., 2024). This is sometimes a valid assumption when reliable and accurate data may be gathered through extensive, automated experimentation, such as the example provided earlier. But in many situations there is a need to consult a human oracle—a medical diagnosis must be provided by a doctor, a loan application must be decided on by a bank manager, and so on. In principle, such cases could also be approached by automated extensive experimentation, but there are ethical or business considerations that limit the extent to which this can be done.

The behavioral sciences, such as the psychology of judgment, decision-making, and behavioral economics, have found that people exhibit systematic biases in the sense of deviations from norms of logic and probability (Kahneman et al., 1982; Gilovich et al., 2002). Whereas such biases might be attributed to the structure of the decision environment or can be viewed as adaptive given a focus on accuracy or transparency (Todd et al., 1999; Katsikopoulos et al., 2020). This structured decision environment refers to the heuristics a human uses in decision-making, which may be biased. It remains a fact that human oracles sometimes provide biased labels, which challenges the common assumption in active learning literature.

This calls for an investigation into the impact of human heuristics used by human oracles on the performance of active learning algorithms(henceforth AL). Such a study would show whether AL algorithms are as effective as commonly assumed. Furthermore, this problem motivates the development of a novel AL algorithm specifically designed to be robust against human-induced biases in the labeling process. Our work successfully addresses both of these objectives.

This research is necessary because investigating the impact of biased oracles will prompt active learning researchers to consider human psychology when designing and evaluating algorithms. By addressing human-induced biases, the development of more robust AL algorithms can lead to more accurate prediction models with fewer labeled instances. This improvement will help practitioners optimize data labeling efforts, enhancing the overall efficiency and performance of AL systems in the presence of biased human inputs. The expected outcomes include more reliable models, reduced labeling costs, and improved algorithmic generalization.

The format of the paper is as follows: Literature pertinent to the investigation is discussed in Section 2. Section 3 provides a methodological overview, including information on the experimental design, AL algorithms, and human heuristic models. Section 4 presents the findings from rigorous investigations conducted in three phases, followed by the Conclusion.

## 2 Background literature

In this section, we provide some background on (*i*) AL algorithms, (*ii*) models of human heuristics, and (*iii*) literature addressing the research problem, which involves the intersection of active learning and biased oracles. We discuss the basic concepts; concrete examples with formal details are given in Section 3, which describes our methodology.

### 2.1 AL algorithms

In what follows, we consider a pool-based sampling scenario where a small number of labeled data points exist and the rest are unlabeled and available at once.

In the first family of AL algorithms, data points are ranked according to metrics such as each point's *uncertainty* or *entropy* (Shannon, 1948). The querying of labels is done based on the rank obtained over the pool of unlabeled data points. They might appear too simple, but such algorithms can be comparatively well-performing (Raj and Bach, 2022; Liu and Li, 2023). Recently, these methods have shown good performance when applied to convolutional auto-encoders for image classification (Roda and Geva, 2024).

The second family of AL algorithms also utilizes uncertainty, though not of data points per se, but rather uncertainty stemming from the predictions of classifiers. Each unlabeled data point is classified in multiple ways to measure this type of uncertainty. In an initial version of this approach (Mitchell, 1982), multiple classifiers are used (these classifiers perform well in the pool of labeled data points). Preference for querying is given to data points receiving contradicting labels from the classifiers. In a variant of this approach (Muslea et al., 2006), called *multi-view learning*, a classifier is trained with different sets of attributes —these are the multiple views—and again, preference is given to data points receiving contradicting labels based on these views.

The third family of AL algorithms considered here tends to outperform the first two families. The approach is to combine uncertainty with what is called *information density*. The aim of information density is to measure how representative an unlabeled data point is of the distribution of all unlabeled data points. The uncertainty and information density measures are typically multiplied to form the combined measure (Settles and Craven, 2008).

### 2.2 Models of human heuristics

Answering Herbert Simon's call for precise models of how people make decisions under realistic conditions of time, information, computation, and other resources (Simon, 1990), the *fast-and-frugal heuristics* approach has provided mathematical models that describe how people judge a quantity, choose one of several options, or classify objects into categories. These heuristics have been empirically validated (Gigerenzer et al., 2011). While fast-and-frugal heuristics can perform competitively to standard statistics and operations research benchmarks or even near-optimally or optimally (Baucells et al., 2008; Katsikopoulos, 2011) under certain conditions, they also commit systematic mistakes. For these reasons, fast-and-frugal heuristics constitute a viable possibility for modeling how human oracles provide labels.

A characteristic property of fast-and-frugal heuristics is that they use a few attributes and combine them in simple ways, for example, by ordering or summing attributes and relying on numerical thresholds. The spectrum of fast-and-frugal heuristics runs from the so-called non-compensatory to fully-compensatory models. Non-compensatory models make decisions without allowing for the values of some attributes to compensate for the values of other attributes. For example, in *fast-and-frugal trees* (Martignon et al., 2008), attributes encountered after an exit is reached cannot reverse the decision embodied in the exit. Of course, this is the case for all decision trees, but fast-and-frugal trees are special cases of decision trees (Section 3). In fully compensatory models, any attribute value can, in principle, compensate for the values of any other attribute. For instance, this is the case in *tallying* (Dawes, 1979), which is a linear model where all attribute weights equal one. Because these two extremes of the fast-and-frugal-heuristics spectrum can cover a large part of the behaviors produced by the heuristics (Katsikopoulos, 2013), thus we consider just fast-and-frugal trees and tallying as models of human heuristics. It must be noted that this work is based on the assumption that fast and frugal heuristics are good models for automating human labeling, which is based on the work of Gigerenzer et al. (2011) and this assumption is not validated in this study.

### 2.3 AL algorithms and biased oracles

A small part of the AL literature has considered biased oracles. Settles (2009) suggested the possibility of incorrect labeling because of the human oracle experiencing fatigue due to, for example, having to provide too many labels. Consistently, some AL algorithms modeling oracles that provide low-quality labels have been developed (Sheng et al., 2008; Groot et al., 2011). However, such algorithms model labeling error as random noise or uniformly distributed error, whereas, as discussed previously, the error is due to human bias and is systematic.

Agarwal et al. (2022) calculated that labeling biases would decrease the predictive accuracy of classifiers by at least 20%. In another approach, Du and Ling (2010) proposed an algorithm with an exploration and exploitation approach by relabeling data points that could be wrongly labeled. The oracle here was modeled based on the assumption that the probability of obtaining biased labels depends on the maximum posterior probability of an instance that is computed with the ground truth labels. We consider this idea promising because it models the effects of oracle behavior.

A detailed discussion of the above literature, along with other notable studies, is presented in Table 1. It is important to highlight that none of the current research models the oracle based on human heuristics or designs AL algorithms with this consideration. This paper addresses this gap by explicitly modeling oracle behavior using well-established human heuristics.

**Table 1:** Literature relevant to AL with biased oracles.

**References**	**Methodology**	**Contribution**	**Research gap**
Agarwal et al. (2022)	Impact of Behavioral biases such as Hot-hand fallacy and Regret aversion bias on Active learning were demonstrated using experiments conducted on the Pancreatic dataset	Established that behavioral bias reduces the classification accuracy of the decision model by at least 20%	The study does not propose novel strategies to mitigate the impact of behavioral bias in models.
Sheng et al. (2008)	The authors analyze various repeated-labeling strategies and introduce a robust technique that combines different measures of uncertainty to selectively choose data points, demonstrating improved results over uniform relabeling.	The key contribution is showing that repeated labeling of selected data points improves label quality and model performance, especially in noisy settings or when processing unlabeled data is costly.	The study does not account for label noise caused by systematic human bias, and the proposed query strategy of repeated labeling for the same query may not be cost-effective across all domains.
Groot et al. (2011)	The researchers use a Gaussian Process framework to model regression with noisy, subjective labels from multiple annotators, demonstrating through experiments that their multi-annotator model outperforms other approaches by effectively capturing annotators' expertise and handling disagreements.	Propose a non-parametric model that can automatically estimate the reliability of annotators from data without requiring prior knowledge.	The estimation of annotator reliability aids in detecting bias but does not contribute to its mitigation.
Du and Ling (2010)	The authors analyze human-like oracles, assuming noise decreases with oracle confidence, and design an active learning algorithm that balances exploration and exploitation. Empirical validation on synthetic and real-world datasets shows its superiority over traditional uncertainty-based methods.	Introduces a realistic model of human oracles in active learning, where labeling noise depends on oracle confidence. The key contribution is a novel algorithm that accounts for example-dependent noise, closely mimicking human behavior.	The oracle confidence model overlooks human heuristics, and the proposed AL algorithm's repeated re-labeling of misclassified data points may not be cost-effective.
Harpale and Yang (2008)	They develop an extended Bayesian active learning strategy tailored to individual users, ensuring that queries are relevant to their potential ratings. A comparative evaluation of benchmark datasets assesses the effectiveness of this personalized method against a well-established baseline.	Presents a novel approach to Collaborative Filtering (CF) that personalizes active learning by querying only items users are likely to rate, thereby addressing the criticality in human labeling.	Oracle modeling does not involve systematic bias injected by human heuristic models.
Raghavan et al. (2006)	The authors extend the traditional active learning framework by incorporating feedback on feature importance alongside labeling instances. They conduct a series of experiments in text categorization, comparing the effects of feature selection and human feedback on classifier performance and developing an algorithm that alternates between labeling features and instances.	The study shows that human feedback on feature relevance improves classifier performance through feature re-weighting, outperforming traditional active learning. Feature labeling is faster than instance labeling, accelerating active learning in applications like news filtering and email classification.	Alternating between querying features and instances may confuse human annotators, complicating implementation. Additionally, the query strategy doesn't account for the heuristics used by annotators.
Hoarau et al. (2024)	The paper introduces two active learning strategies, Klir uncertainty sampling and evidential epistemic uncertainty sampling, both based on belief function theory, to address the exploration-exploitation trade-off and handle reducible uncertainty.	The proposed methods incorporate oracle uncertainty into active learning and demonstrate superior performance compared to traditional uncertainty sampling in experimental evaluations, simplifying computational processes without relying on specific observations.	Decision strategies used by humans were not considered toward the computation of oracle uncertainty.

## 3 Methodology

**Figure 1:**
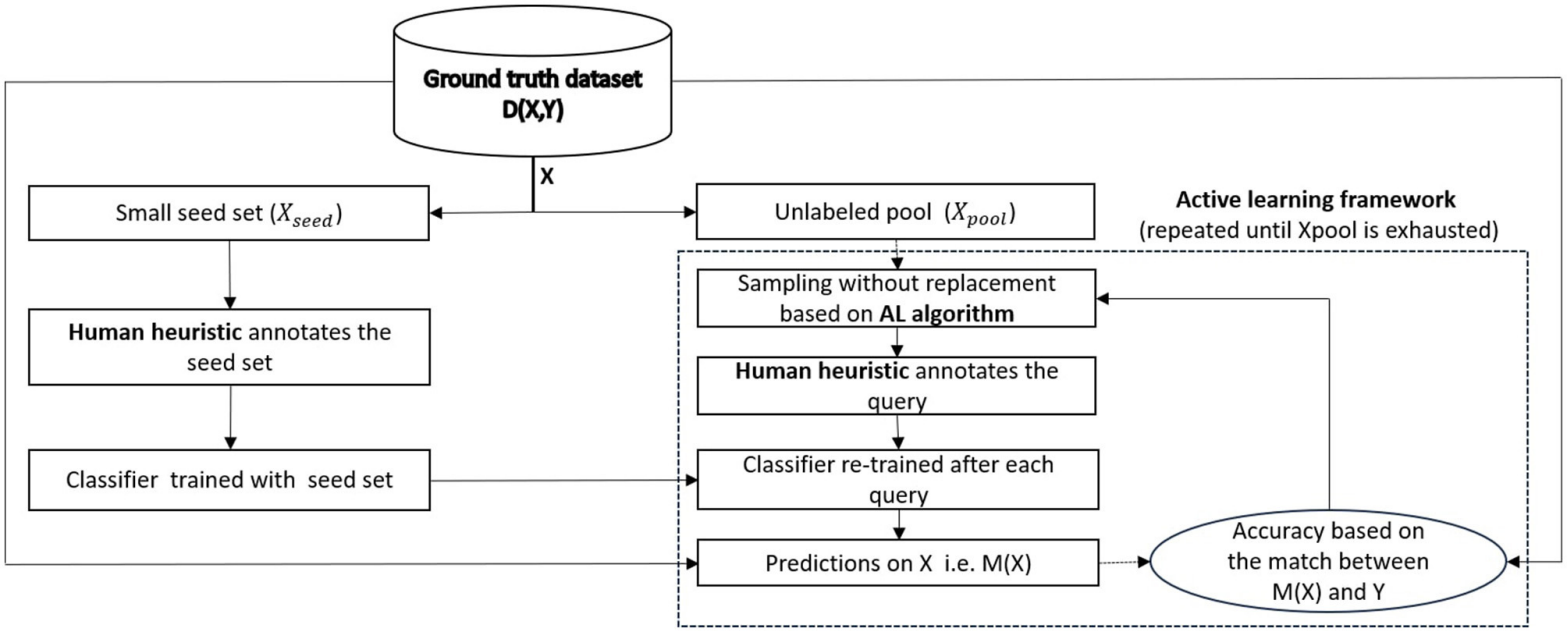
Methodological framework.

The methodological framework is presented in Figure 1. Out of the dataset *D*(*X, Y*), where *Y* represents the ground truth labels for the set of data points *X* characterized by their attributes, a small fraction *X*_*seed*_⊂*X* is used to train the classifier with the labels provided by the human heuristic. This operation is portrayed in the left part of the figure. On the other hand, as seen in the right part of the figure, the remaining large pool of data points *X*_*pool*_⊂*X* is used by the AL algorithm to identify the next data point to query. The queried data point and its heuristic-provided label are used to retrain the classifier. The accuracy of the whole model *M* is recomputed after each query.

We use three standard classifiers, logistic regression (LR), random forest (RF), and support vector machines (SVM) that were predominantly used in AL literature (Yang and Loog, 2018; Gu et al., 2014; Kremer et al., 2014).

### 3.1 AL algorithms

This section includes a description of three well-known AL algorithms selected for this study (one from each AL family discussed in section 2.1), which are not only widely accepted and commonly used for benchmarking but also well-performing to date (Liapis et al., 2024; Tan et al., 2024; Moles et al., 2024). Following this, the novel inverse information density method is presented.

#### 3.1.1 Entropy

The entropy *E*(*x*) of a data point *x* measures the information required to label this data point with certainty. The following equation calculates this value, where *p*_*M*_(*y*_*i*_/*x*) denotes the probability of a data point *x* belonging to class *y*_*i*_, ranging over *K* possible label assignments. This probability is derived from the model M, which is trained using the labels acquired up to the previous query.


(1) $$\begin{array}{l} E\left(x\right)=-\overset{K}{\underset{i=1}{\sum }}p_{M}\left(y_{i}/x\right)\log\left(p_{M}\left(y_{i}/x\right)\right) \end{array}$$

The unlabeled data point with maximum *E*(*x*) is chosen to be queried:


(2) $$\begin{array}{l} x^{*}=\underset{x\in X_{U}}{arg max}\left[E\left(x\right)\right] \end{array}$$

#### 3.1.2 Multi-view learning (MVL) with co-testing

The following pseudo-code describes an algorithm that incorporates uncertainty stemming from using different classification processes. There is a single classifier, trained with two different sets, called *views*, of attributes (Step 1). Unlabeled data points with different predicted labels in the two views form the co-testing set (Steps 2 and 3), where the point with maximum entropy is chosen to be queried (Step 4).

**Input:** Labeled set of data points (*X*_*L*_, *Y*_*L*_), unlabeled pool of data points (*X*_*U*_)

1: The labeled data (*X*_*L*_) is split into two attribute sets (views), $X_{L}^{1}$ and $X_{L}^{2}$, and trains two classifiers using these different views.

2: For each unlabeled data point *x* in *X*_*U*_, the predictions from the two classifiers are compared.

3: If the classifiers disagree on the label for *x*, this point is added to the co-testing set *C*. If no disagreements are found, all points in *X*_*U*_ are added to *C*.

4: x* = argmax_*x*∈*C*_[*E*(*x*)]

**Output:** Data point x* to query

It is important to emphasize that the labeled set (*X*_*L*_, *Y*_*L*_) is updated with the newly acquired labels after each query. Similarly, the queried data point is removed from the pool of unlabeled data (*X*_*pool*_) following every query, consistent with standard practices in other algorithms.

#### 3.1.3 Conventional information density (CID)

This algorithm evaluates data points on two measures. The first measure captures the uncertainty of a data point's most probable label, as formulated below, where *K* represents the number of possible labels.


(3) $$\begin{array}{l} U\left(x\right)=1-\underset{i\in 1,..K}{\max}P_{M}\left(y_{i}|x\right) \end{array}$$

The second measure captures how representative a data point of the distribution of unlabeled data points *x*_*u*_ by using the cosine similarity function *sim* (Settles and Craven, 2008), where U represents the size of the unlabeled set *X*_*u*_ as shown in the following.


(4) $$\begin{array}{l} R\left(x\right)=\frac{1}{U}\underset{x_{u}\in X_{U}}{\sum }sim\left(x,x_{u}\right) \end{array}$$

The unlabeled data point with the maximum product of the two measures is chosen to be queried:


(5) $$x*=\;\underset{x\in X_{U}}{\arg\max}[U(x)*(R(x)]$$

#### 3.1.4 The proposed Inverse Information Density (IID)

The aim of this algorithm is to achieve robustness to labeling bias. We design an algorithm inspired by human psychology. Human heuristics are robust across a host of real-world situations, including prediction in classification tasks (Gigerenzer et al., 2011; Katsikopoulos et al., 2020).

The IID algorithm shares the basic concepts of the CID algorithm, but it employs them differently. There are two differences. First, IID does not use all available attributes but only the attributes that a statistical test (Pearson correlation test) has found to be significantly related to ground truth labels. People's fast-and-frugal heuristics routinely narrow down the set of available attributes, and this has been shown to, under some conditions, enhance their predictive accuracy (Baucells et al., 2008; Simşek, 2013). In IID, representativeness is computed using the *narrowed* set of attributes (*N*) significantly correlated with the previous set of labels obtained and the function *sim* stands for Euclidean distance.

The second difference between IID and CID is that, in IID, representativeness is seen as a reason to *not* query a data point. People have a natural tendency to explore uncharted territory, sometimes with good success, as in armed bandit problems (Stojić et al., 2015; Brown et al., 2022), and the IID tweak in using information density captures this tendency.

The following pseudo-code describes the IID algorithm.

**Input:** Labeled set of data points (*X*_*L*_, *Y*_*L*_), unlabeled pool of data points (*X*_*U*_), attribute list (*ATT*), $s=\underset{x_{1},x_{2}\in X}{\sum }sim\left(x_{1},x_{2}\right)$

1: For all *att* in *ATT*:

        If *Corr*_*att*_(*X*_*L*_, *Y*_*L*_) ≠0 (α = 0.001):

           *N* ← *att*

2: For all *x* in *X*_*U*_:

            $R\left(x\right)=\frac{1}{s}\underset{N;x_{u}\in X_{U}}{\sum }sim\left(x,x_{u}\right)$

3: x* = argmax_*x*∈_*X*__*U*__[*U*(*x*)−*R*(*x*)]

**Output**: Data point x* to query

The IID algorithm begins by identifying the subset of attributes *N* that are significantly correlated with the labels in the labeled set (*X*_*L*_, *Y*_*L*_), using a Pearson correlation test at a significance level α = 0.001 (Step 1). For each data point *x* in the unlabeled pool *X*_*U*_, the representativeness *R*(*x*) is computed based on its similarity to other points in *X*_*U*_, using the Euclidean distance *sim*(*x, x*_*u*_) specifically focusing on attributes contained in N (Step 2). Finally, the algorithm selects the data point x^*^ that has the maximum difference between uncertainty *U*(*x*) and representativeness *R*(*x*).

### 3.2 Models of human heuristics

#### 3.2.1 Fast-and-frugal trees

A fast-and-frugal tree (*FFT*) is a tree for making classifications such that it (i) always has an exit after it queries an attribute (two exits after it queries the last attribute), (ii) has only a ‘few' attributes (a common default value is three attributes) and (iii) queries each attribute once and does not query multiple attributes together.

These three conditions jointly imply that fast-and-frugal trees are, all else being equal, sparser than standard classification trees. In general, trees are made sparser by using fewer attributes or by using each attribute fewer times; methods of statistical induction of trees include pruning modules that pursue these goals (Bertsimas and Dunn, 2017; Breiman et al., 1984). Fast-and-frugal trees further increase sparsity by using each attribute at most once.

**Figure 2:**
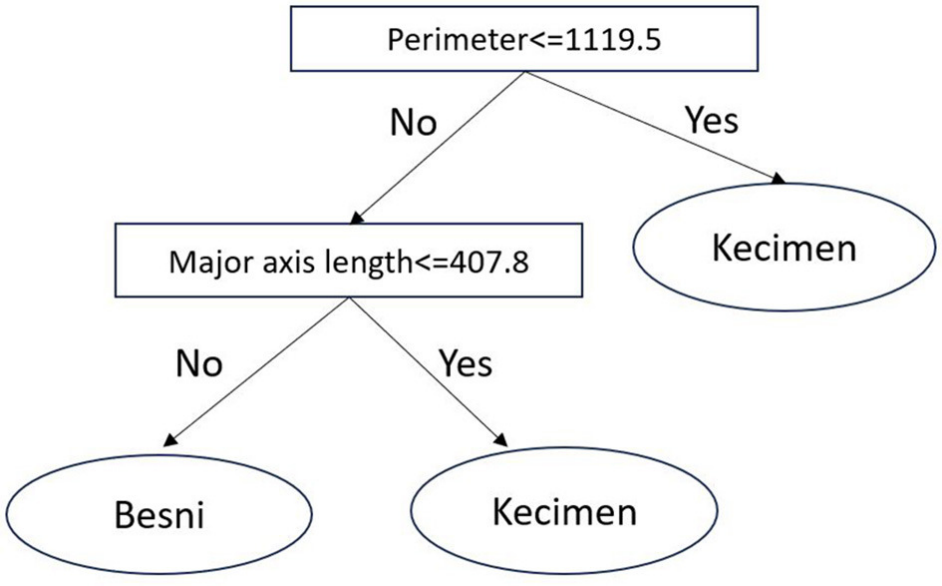
A fast-and-frugal tree for predicting raisin type.

There are several statistical and qualitative methods for inducing fast-and-frugal trees from data (Katsikopoulos et al., 2020). Here, we build fast-and-frugal trees via the fan algorithm (Phillips et al., 2017), where attributes were binarized using a median split. Additionally, the maximum depth of the tree is set to three. An example fast-and-frugal tree induced in the 'Raisin' dataset (Cinar et al., 2020), where the task is to predict the type of raisin (Kecimen or Besni) based on two morphological features of raisins, is shown in Figure 2.

#### 3.2.2 Tallying

According to (Martignon et al., 2008), a tallying model is a unit-weight linear model for making classifications, with the number of its parameters equalling the number of possible classes minus one.

For example, assume that there are two classes, *C*_1_, *C*_2_, and one wishes to classify a data point *x* with binary attribute values *x*_*i*_, *i* = 1, ..., *n* to one class. Tallying can be described by the following, where the parameter *k* can take any integer value from 1 to *n*.


(6) $$\begin{array}{l} \text{Assign}x\to C_{1}\text{iff}\underset{i=1,...,n}{\sum }x_{i}>k \end{array}$$

## 4 Results

We use 15 datasets from the UCI ML Repository (Kelly et al., n.d.), where people routinely provide labels. Datasets come mostly from health but other domains too, such as marketing and transportation. For brief descriptions of the datasets, see Supplementary material A. We chose datasets with two possible classes because there is more empirical evidence for people's use of fast-and-frugal heuristics in such classification tasks (Katsikopoulos et al., 2020) It must be noted that all the datasets used for the study were used and cited by multiple published works (Jalali et al., 2017; Xie et al., 2019).

Our investigations were carried out in three phases. In the first phase, a hypothesis on the nature of human heuristics is proposed, and its validity is empirically explored to comprehend the points susceptible to labeling bias. The second phase aims to establish that our algorithm has the characteristics that make it robust toward such bias. An evaluation of the performance of Active learning algorithms is provided in the final section.

### 4.1 Phase 1: experimental validation on the hypothesized nature of human heuristics

To understand the nature of human heuristics, we develop a hypothesis. We hypothesize that data points farther away from the data points with median values for the most important attributes are more likely to be accurately labeled by human heuristics.

**Figure 3:**
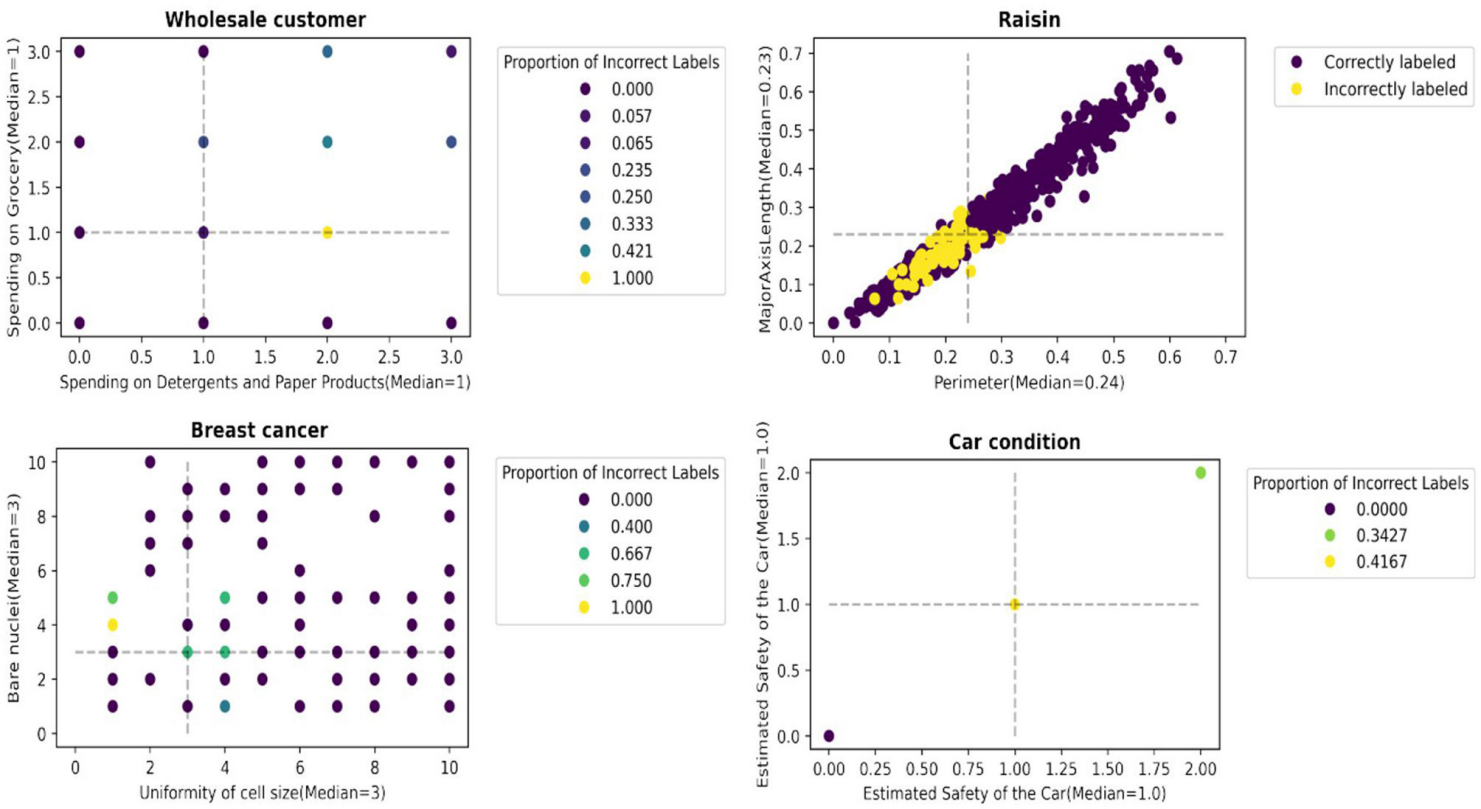
Data points farther away from the data points with median values for the most important attributes are more likely to be accurately labeled by the fast-and-frugal tree.

**Figure 4:**
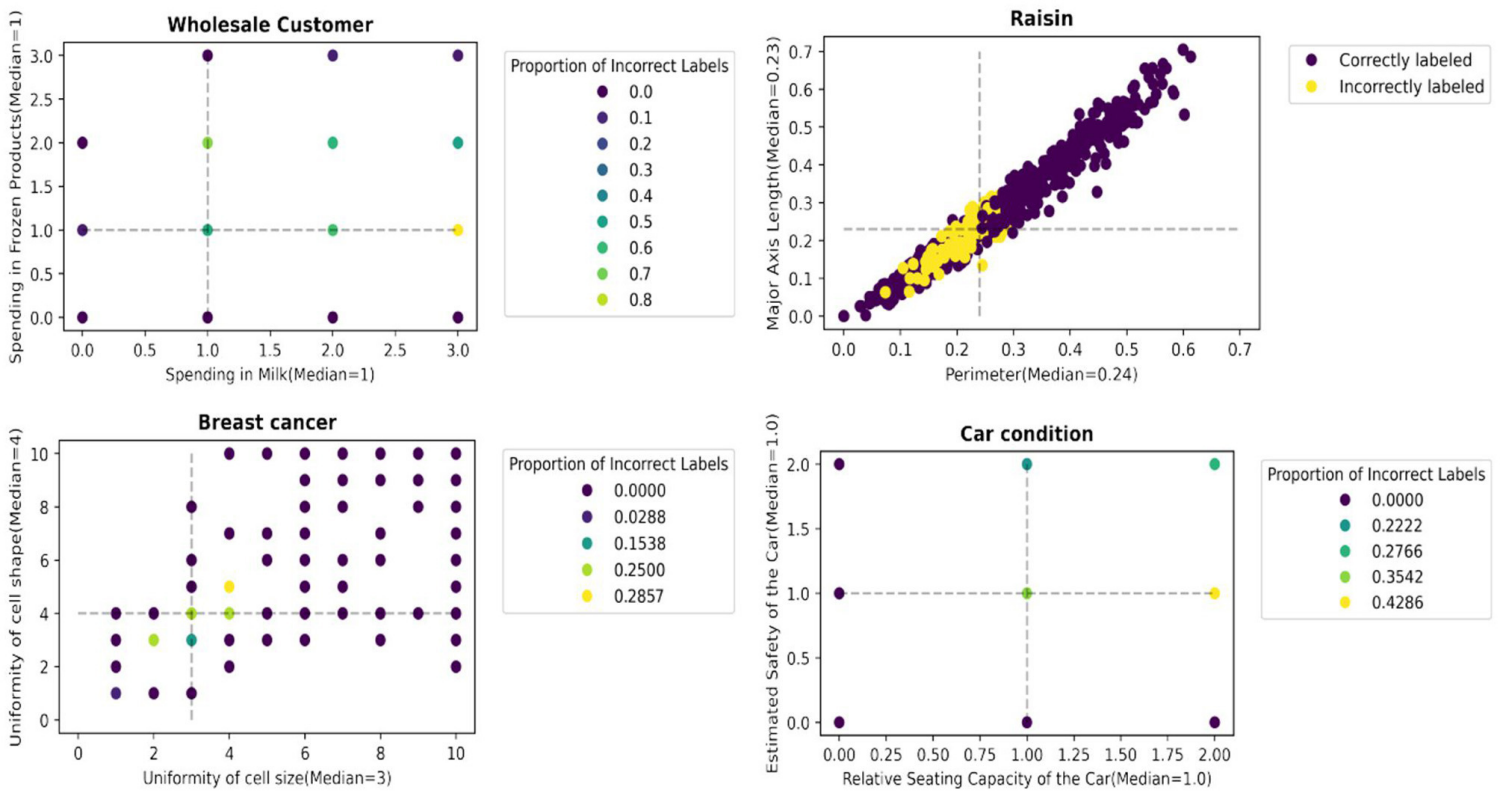
Data points farther away from the data points with median values for the most important attributes are more likely to be accurately labeled by tallying.

We report an empirical test that supports the hypothesis. Figures 3, 4 illustrate the distribution of correctly/incorrectly labeled data points with respect to important attribute values used by heuristics in the decision-making process. Notably, the correctly classified points, represented in purple, tend to lie farther from the median attribute values, marked by the dotted lines. This pattern holds overall for all 30 scenarios ( 15 datasets x 2 heuristics), as shown in Supplementary material B. These results support our assertion that data points with attribute values deviating from their population median are more likely to be labeled correctly by the heuristics.

### 4.2 Phase 2: assessment on AL algorithm's robustness for human heuristics

The robustness of an AL algorithm toward labeling bias depends on (*i*) the independence of the algorithm on labeling accuracy and (*ii*) the ability of the algorithm to identify and query data points that are more likely to be accurately labeled. In this section, we find that the IID algorithm is well-suited for the aforementioned factors. Therefore, we hypothesize that the IID algorithm would perform better than the existing ones.

Factor (i) favors the information density algorithms, CID and IID. This is so because Entropy and MVL only rely on *E*(*x*) and *U*(*x*) that are dependent on labeling accuracy, whereas the information density algorithms also use *R*(*x*).

When the experimentally validated hypothesis is combined with the fact that IID prefers querying such points more than CID (because only in IID R(x) measures how close a data point is to the data points with median values for the most important attributes), They jointly imply that IID has a higher ability than CID to identify and query data points more likely to be accurately labeled.

**Figure 5:**
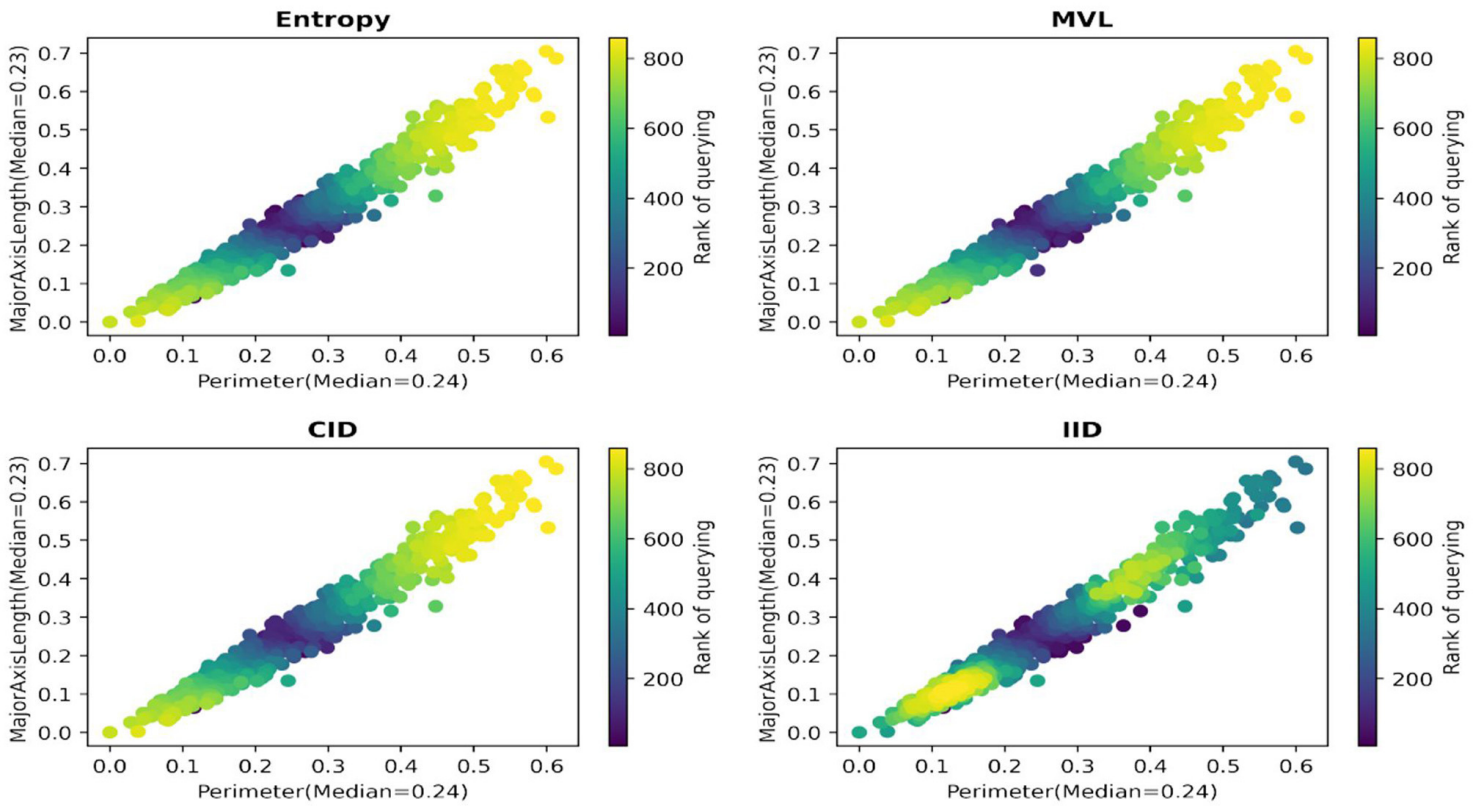
Rank of querying for the Raisin dataset.

In Figure 5, evidence is provided for the Raisin dataset (one run, the fast-and-frugal tree provided labels) that IID is the only algorithm that prefers querying the data points farther away from the data points with median values for the most important attributes. This pattern holds overall for the 30 scenarios (see Supplementary material B).

### 4.3 Phase 3: performance of AL algorithms

Experiments were carried out on datasets using human heuristics and AL query methods. In every iteration, a randomly chosen seed set (*X*_*seed*_) labeled with the human heuristic was used to train a classifier, and the remaining pool (*X*_*pool*_) was used by the AL query strategies to choose the data points to query. The classifier was re-trained to predict the entire dataset after every query. The above process was pursued for 30 iterations by varying the *X*_*seed*_ and *X*_*pool*_ chosen from X after each iteration.

**Figure 6:**
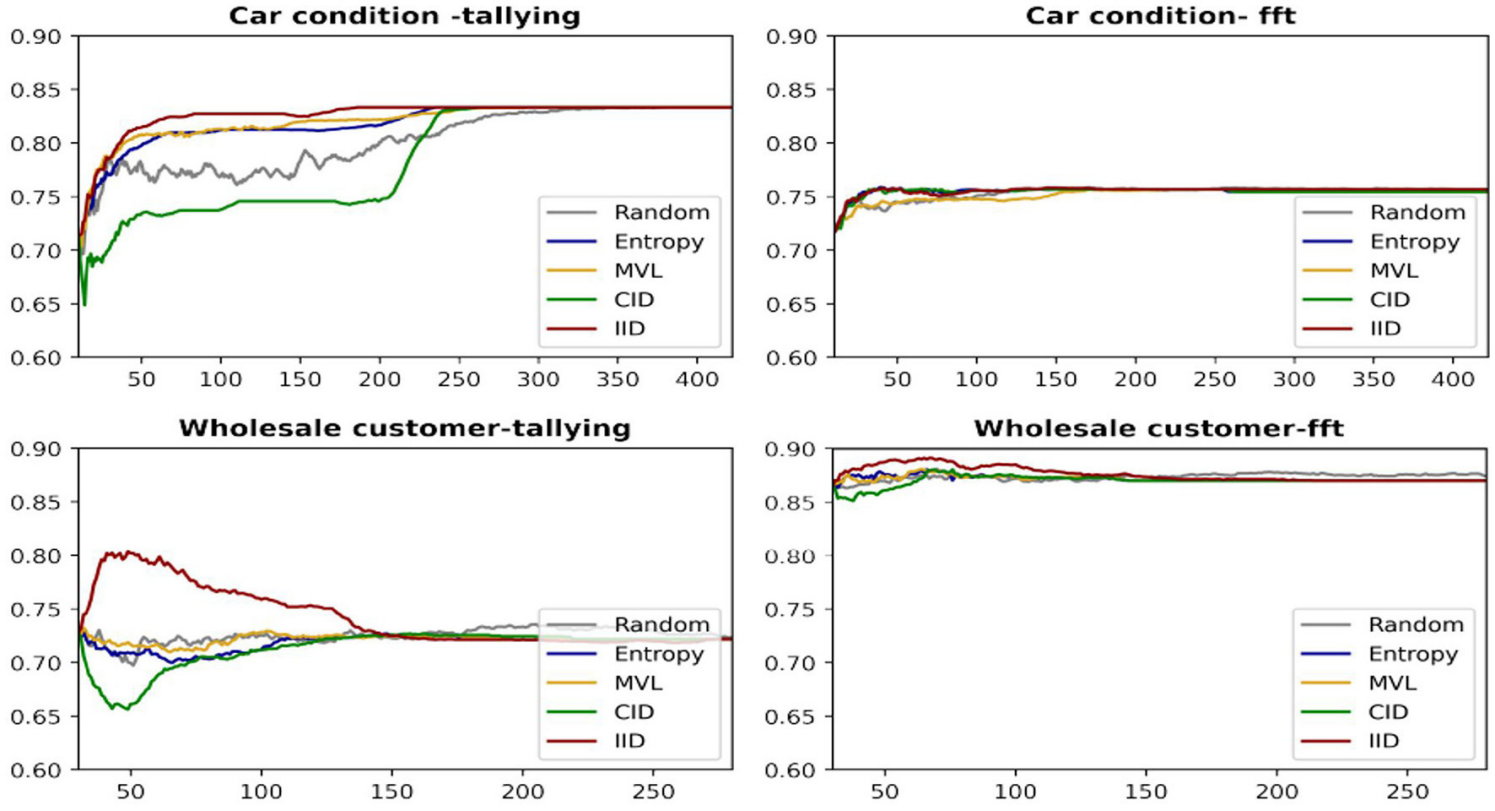
Learning curves for two datasets using the LR classifier.

Figure 6 provides learning curves (accuracy as a function of the number of data points queried) for the four AL algorithms(averaged over all iterations), including random sampling as a benchmark for two datasets and both human heuristics. The IID algorithm has superior performance in these cases.

Tables 2–5 report the area under the learning curve for both human heuristics for the LR classifier. Bold font denotes the algorithm with the best performance, and underlined font denotes that an algorithm performed worse than random. It can be inferred that irrespective of the performance metric, entropy sampling consistently outperforms other methods in most scenarios when ground truth labels are available. Additionally, the proposed IID algorithm demonstrates superior performance when heuristics are employed for labeling. Table 6 summarizes these findings by illustrating the frequency of algorithms that exhibit optimal performance. Similar results were obtained for the RF and SVM classifiers (see Supplementary material C).

**Table 2:** Area under the learning curve (accuracy) for all 15 datasets and the LR classifier.

**Dataset**	**Labels provided by**	**Max. value**	**Random**	**Entropy**	**MVL**	**CID**	**Proposed IID**
Car condition	Ground truth	422	356.62	**358.84**	357.92	357.50	358.83
	FFT	422	318.16	318.58	317.25	318.50	**318.91**
	Tallying	422	338.44	345.43	346.33	330.92	**348.63**
Breast cancer	Ground truth	439	417.97	419.77	419.50	419.41	**420.85**
	FFT	439	410.50	411.70	411.55	**411.92**	411.77
	Tallying	439	418.48	418.67	418.34	418.40	**419.27**
Wholesale customer	Ground truth	279	247.11	249.86	249.88	249.50	**250.53**
	FFT	279	243.82	243.33	243.27	242.88	**244.36**
	Tallying	279	202.25	200.65	201.42	199.31	**206.40**
Raisin	Ground truth	852	729.16	**734.42**	733.16	731.80	734.14
	FFT	852	722.54	728.78	728.46	728.36	**729.97**
	Tallying	852	715.86	**715.88**	715.54	715.94	714.65
Wine	Ground truth	161	155.83	158.01	158.00	157.82	**158.09**
	FFT	161	153.18	154.38	154.25	154.04	**154.40**
	Tallying	161	152.46	**153.11**	153.03	153.05	152.76
Maternal health	Ground truth	958	669.81	**691.84**	688.49	689.04	684.78
	FFT	958	619.51	633.70	633.37	625.60	**641.78**
	Tallying	958	579.58	593.34	596.42	594.30	**596.88**
Algerian forest	Ground truth	231	187.20	194.57	**195.43**	192.67	194.34
	FFT	231	190.31	195.68	195.58	193.10	**195.80**
	Tallying	231	180.00	185.02	185.06	183.20	**186.84**
Contraceptive	Ground truth	1222	798.77	821.41	**822.81**	821.30	818.55
	FFT	1222	700.78	697.07	699.99	693.52	**700.99**
	Tallying	1222	707.88	744.96	746.24	741.94	**747.03**
Echocardiogram	Ground truth	55	51.96	**52.07**	**52.07**	51.65	**52.07**
	FFT	55	51.90	51.42	51.43	51.36	**51.42**
	Tallying	55	47.71	45.61	45.45	45.24	**45.97**
Chronic kidney disease	Ground truth	143	140.84	**142.86**	**142.86**	**142.87**	**142.86**
	FFT	143	140.84	**142.86**	**142.86**	142.59	**142.86**
	Tallying	143	139.08	141.20	141.19	141.04	**141.43**
Cervical cancer	Ground truth	65	59.09	**60.95**	60.88	60.33	60.86
	FFT	65	56.06	**57.25**	56.91	56.20	57.24
	Tallying	65	55.57	56.29	56.24	56.11	**56.34**
Parkinsons disease	Ground truth	186	156.10	**161.42**	161.35	159.84	160.76
	FFT	186	145.33	145.06	145.39	144.75	**145.75**
	Tallying	186	133.71	130.57	130.51	130.88	**131.97**
Indian liver patient	Ground truth	568	406.85	406.54	406.57	**406.63**	406.45
	FFT	568	384.03	371.19	375.64	357.64	**384.57**
	Tallying	568	366.08	356.93	359.72	349.50	**369.22**
Happiness survey	Ground truth	135	82.45	**86.60**	85.95	83.86	86.53
	FFT	135	82.60	82.46	82.90	82.14	**84.03**
	Tallying	135	88.20	89.11	**89.13**	89.03	89.03
Breast cancer-prognostic	Ground truth	185	146.48	**150.13**	150.06	146.88	149.97
	FFT	185	135.80	137.02	137.91	137.18	**138.40**
	Tallying	185	108.36	114.57	114.80	110.38	**117**

Algorithm with best performance is bolded.

**Table 3:** Area under the learning curve (Precision) for all 15 datasets and the LR classifier.

**Dataset**	**Labels provided by**	**Max. value**	**Random**	**Entropy**	**MVL**	**CID**	**Proposed IID**
Car condition	Ground truth	422	345.60	349.71	348.91	348.35	**349.71**
	FFT	422	294.19	294.79	292.03	294.65	**295.38**
	Tallying	422	323.53	331.61	331.20	326.07	**332.62**
Breast cancer	Ground truth	439	418.09	419.85	419.57	419.57	421.21
	FFT	439	412.26	413.92	413.63	**413.98**	413.92
	Tallying	439	419.16	419.22	418.85	418.87	419.86
Wholesale customer	Ground truth	279	241.69	245.52	245.81	244.78	**247.43**
	FFT	279	234.71	234.13	234.00	233.51	**235.94**
	Tallying	279	207.60	207.35	207.56	206.78	**209.64**
Raisin	Ground truth	852	703.26	**707.83**	706.99	707.75	706.94
	FFT	852	699.78	700.99	701.06	700.94	**701.75**
	Tallying	852	**690.00**	688.22	688.06	688.99	686.74
Wine	Ground truth	161	155.46	**157.75**	**157.77**	157.64	157.66
	FFT	161	154.08	**156.04**	156.02	155.95	**155.59**
	Tallying	161	151.34	**152.47**	152.33	152.18	152.16
Maternal health	Ground truth	958	660.90	**688.79**	682.17	684.92	680.88
	FFT	958	616.45	699.71	690.80	701	**702.08**
	Tallying	958	607.16	614.20	616.04	611.27	**616.52**
Algerian forest	Ground truth	231	190.11	**194.69**	**195.38**	193.76	194.77
	FFT	231	194.04	195.09	195.75	194.05	**195.93**
	Tallying	231	185.42	187.77	187.97	187.03	**188.44**
Contraceptive	Ground truth	1,222	792.33	825.85	**826.80**	851	817.34
	FFT	1,222	705.40	708.17	707.88	707.55	**708.87**
	Tallying	1222	708.28	739.36	738.48	732.74	**741.25**
Echocardiogram	Ground truth	55	51.07	**50.65**	**50.62**	50.28	**50.63**
	FFT	55	50.59	49.85	49.85	49.82	49.85
	Tallying	55	46.63	44.89	44.71	45.68	45.20
Chronic kidney disease	Ground truth	143	141.41	**142.91**	**142.91**	**142.91**	**142.90**
	FFT	143	141.41	**142.91**	**142.91**	**142.91**	**142.90**
	Tallying	143	140.53	141.79	141.79	141.68	**141.95**
Cervical cancer	Ground truth	65	60.56	**61.78**	61.58	61.15	61.61
	FFT	65	58.46	58.11	57.88	57.90	58.19
	Tallying	65	54.47	54.39	54.45	54.32	**54.48**
Parkinson's disease	Ground truth	186	154.85	**164.80**	164.60	162.70	162.02
	FFT	186	135.16	136.92	137.12	136.53	**137.60**
	Tallying	186	129.59	128.98	129.00	129.21	129.56
Indian liver patient	Ground Truth	568	300.23	273.78	**278.85**	333.81	276.62
	FFT	568	339.09	359.30	359.96	358.81	**361.52**
	Tallying	568	356.01	367.45	366.64	366.26	**368.01**
Happiness survey	Ground Truth	135	82.38	**88.47**	**88.51**	83.34	87.35
	FFT	135	84.92	84.57	84.91	84.58	**85.37**
	Tallying	135	88.32	**89.10**	**89.13**	89.05	89.03
Breast cancer-prognostic	Ground Truth	185	140.84	**159.76**	158.26	142.29	158.26
	FFT	185	119.62	121.75	122.22	121.40	**123.02**
	Tallying	185	116.01	116.26	116.65	115.82	**116.89**

Algorithm with best performance is bolded. Underlined font denotes that an algorithm performed worse than random.

**Table 4:** Area under the learning curve (Recall) for all 15 datasets and the LR classifier.

**Dataset**	**Labels provided by**	**Max. value**	**Random**	**Entropy**	**MVL**	**CID**	**Proposed IID**
Car condition	Ground truth	422	332.47	334.70	333.18	334.95	**334.85**
	FFT	422	290.68	291.12	288.96	290.76	**292.71**
	Tallying	422	334.99	339.64	339.58	339.82	**340.55**
Breast cancer	Ground truth	439	417.91	419.62	419.36	419.30	**420.62**
	FFT	439	409.49	410.60	410.31	**410.80**	410.56
	Tallying	439	417.95	418.14	417.84	417.87	**418.59**
Wholesale customer	Ground truth	279	236.42	**239.07**	238.85	239.01	238.74
	FFT	279	238.78	238.02	237.89	237.97	**238.09**
	Tallying	279	223.07	222.52	222.85	221.60	**225.60**
Raisin prediction	Ground truth	852	702.00	**705.42**	704.90	705.17	704.99
	FFT	852	696.41	698.92	698.99	698.88	**699.85**
	Tallying	852	687.29	**686.95**	686.75	687.67	685.69
Wine prediction	Ground truth	161	152.40	**155.84**	155.75	155.80	155.23
	FFT	161	152.88	157.22	157.09	157.29	**157.77**
	Tallying	161	151.28	154.61	154.38	154.36	**155.47**
Maternal health	Ground truth	958	649.37	**686.61**	681.64	681.25	679.71
	FFT	958	667.56	671.24	669.63	676.82	**677.69**
	Tallying	958	604.03	613.86	616.38	611.95	**616.69**
Algerian prediction	Ground truth	231	186.86	193.78	**194.84**	191.94	193.91
	FFT	231	187.95	195.66	195.50	191.74	**195.76**
	Tallying	231	183.71	187.69	188.02	186.57	**189.03**
Contraceptive	Ground truth	1,222	770.91	778.84	**783.65**	768.69	776.65
	FFT	1,222	707.59	709.00	709.26	707.49	**709.84**
	Tallying	1,222	710.49	741.70	740.83	732.77	**743.62**
ECG preds	Ground truth	55	51.75	52.37	**52.66**	50.89	52.39
	FFT	55	52.52	52.40	52.40	52.38	52.40
	Tallying	55	49.88	48.45	48.32	48.24	48.79
Chronic kidney	Ground truth	143	138.57	**142.74**	**142.74**	**142.76**	**142.73**
	FFT	143	138.57	**142.74**	**142.74**	**142.76**	**142.73**
	Tallying	143	136.09	139.69	139.69	139.40	**140.14**
Cervical cancer	Ground truth	65	55.27	**58.62**	58.53	57.80	58.46
	FFT	65	50.46	52.18	51.41	50.27	**52.42**
	Tallying	65	53.48	55.36	**55.51**	55.16	**55.55**
Parkinsons	Ground truth	186	129.25	**139.75**	139.51	137.03	**139.70**
	FFT	186	142.44	149.08	149.21	148.34	**149.92**
	Tallying	186	141.05	141.31	141.33	141.49	**141.99**
Indian liver	Ground truth	568	287.23	286.02	286.12	287.00	285.55
	FFT	568	339.35	372.28	372.51	372.08	**373.77**
	Tallying	568	370.58	**382.88**	382.46	380.60	376.35
Happiness survey	Ground truth	135	81.68	**85.65**	85.59	82.04	85.18
	FFT	135	84.11	83.71	84.12	83.68	**84.81**
	Tallying	135	88.41	89.22	89.24	89.15	**89.34**
Breast cancer-prognostic	Ground truth	185	104.96	**113.42**	113.16	105.77	112.74
	FFT	185	122.22	124.83	123.78	123.77	**126.04**
	Tallying	185	123.63	125.08	125.66	123.69	**126.03**

Algorithm with best performance is bolded. Underlined font denotes that an algorithm performed worse than random.

**Table 5:** Area under the learning curve (F1 score) for all 15 datasets and the LR classifier.

**Dataset**	**Labels provided by**	**Max. value**	**Random**	**Entropy**	**MVL**	**CID**	**Proposed IID**
Car condition	Ground truth	422	338.91	342.04	340.87	341.52	**342.12**
	FFT	422	292.42	292.94	290.49	292.70	**294.04**
	Tallying	422	329.16	335.58	335.34	332.80	**336.54**
Breast cancer	Ground truth	439	418.00	419.74	419.47	419.44	**420.91**
	FFT	439	410.87	**412.25**	411.96	412.38	**412.23**
	Tallying	439	418.56	418.68	418.34	418.37	**419.23**
Wholesale customer	Ground truth	279	239.03	242.25	242.28	241.86	**243.01**
	FFT	279	236.73	236.06	235.93	235.72	**237.01**
	Tallying	279	215.06	214.67	214.93	213.93	**217.33**
Raisin	Ground truth	852	702.63	706.62	705.94	706.46	**705.97**
	FFT	852	698.09	699.95	700.02	699.91	**700.80**
	Tallying	852	688.64	687.59	687.40	688.33	686.21
Wine	Ground truth	161	153.92	**156.79**	**156.75**	156.71	156.44
	FFT	161	153.48	**156.67**	156.56	**156.62**	**156.63**
	Tallying	161	151.31	153.53	153.35	153.27	**153.80**
Maternal health	Ground truth	958	655.09	**687.70**	681.91	683.08	680.29
	FFT	958	640.99	685.18	680.05	688.70	**689.67**
	Tallying	958	605.59	614.03	616.21	611.61	**616.60**
Algerian prediction	Ground truth	231	188.47	194.23	**195.11**	192.84	194.34
	FFT	231	190.95	195.37	195.63	192.89	**195.84**
	Tallying	231	184.56	187.73	188.00	186.80	**188.74**
Contraceptive	Ground truth	1222	781.47	801.65	804.65	**807.75**	796.47
	FFT	1222	706.49	708.58	708.57	707.52	**709.35**
	Tallying	1222	709.38	740.53	739.65	732.76	**742.43**
ECG preds	Ground truth	55	51.41	51.49	**51.62**	50.58	51.49
	FFT	55	51.54	51.09	51.10	51.07	51.10
	Tallying	55	48.20	46.60	46.44	46.40	46.93
Chronic kidney	Ground truth	143	139.97	**142.82**	**142.82**	**142.81**	**142.84**
	FFT	143	139.97	**142.82**	**142.82**	**142.81**	**142.84**
	Tallying	143	138.28	140.73	140.73	140.53	**141.04**
Cervical cancer	Ground truth	65	57.79	**60.16**	**60.02**	59.43	**59.99**
	FFT	65	54.16	54.98	54.45	53.82	**55.16**
	Tallying	65	53.97	**55.01**	**54.98**	54.73	54.87
Parkinsons	Ground truth	186	140.90	**151.25**	151.02	148.77	150.03
	FFT	186	138.71	142.74	142.91	142.19	**143.50**
	Tallying	186	135.07	**135.49**	134.89	135.07	134.86
Indian liver	Ground truth	568	293.59	279.77	282.43	281.02	**308.64**
	FFT	568	339.22	365.68	366.13	365.32	**367.54**
	Tallying	568	363.15	**375.00**	374.39	373.29	374.00
Happiness survey	Ground truth	135	82.03	**87.03**	**87.02**	82.69	86.25
	FFT	135	84.51	84.14	84.51	84.13	**85.09**
	Tallying	135	88.37	89.16	**89.18**	89.10	**89.19**
Breast cancer-prognostic	Ground truth	185	120.28	**132.66**	131.96	121.34	131.69
	FFT	185	120.91	123.27	122.99	122.57	**124.51**
	Tallying	185	119.70	120.51	120.99	119.63	**121.29**

Algorithm with best performance is bolded. Underlined font denotes that an algorithm performed worse than random.

**Table 6:** Recurrence in best performance across all datasets (from Tables 2–5).

**Labels provided by**	**Entropy**	**MVL**	**CID**	**Proposed IID**
**Metric: accuracy**				
Ground truth	**9**	4	3	3
FFT	2	1	1	**13**
Tallying	0	2	1	**12**
**Metric: precision**				
Ground truth	**10**	7	2	4
FFT	3	2	3	**14**
Tallying	2	1	1	**11**
**Metric: recall**				
Ground truth	**9**	4	1	4
FFT	2	2	3	**13**
Tallying	2	2	1	**13**
**Metric: F1 score**				
Ground truth	**7**	6	2	7
FFT	4	2	3	**15**
Tallying	3	2	1	**11**

To provide insights into the overall trends in the behavior of active learning (AL) algorithms, irrespective of the specific prediction tasks, we utilize Table 7. This table presents the average performance of all classifiers across all datasets, using accuracy as the primary metric. The best performances within 0.05 are highlighted in bold. The proposed IID method showed an overall effectiveness improvement of 19.8% compared to Entropy sampling, which was the best-performing alternative. This boost in performance is primarily due to the Inverse Information Density (IID) metric, which complements the uncertainty measure captured by entropy sampling. Notably, the improvement increases to 87% when labels are generated by human heuristic models, further demonstrating the suitability of the proposed method in such environments, as anticipated. However, this summary masks data-specific variations in performance, which serves as a notable caveat.

**Table 7:** Average area under the learning curves, based on accuracy, across all datasets, presented for each classifier.

**Labels provided by**	**Max. value (approx.)**	**Random**	**Entropy**	**MVL**	**CID**	**Proposed IID**
**Classifier: LR**						
Ground truth	393	307.08	**312.62**	312.33	311.41	311.97
FFT	393	290.36	291.37	291.78	289.32	**293.48**
Tallying	393	282.24	286.09	286.63	283.95	**288.23**
**Classifier: RF**						
Ground truth	393	331.18	**340.20**	339.29	338.08	339.64
FFT	393	293.41	293.98	**294.03**	292.04	**294.01**
Tallying	393	288.49	288.90	288.93	288.75	**289.08**
**Classifier: SVM**						
Ground truth	393	314.07	**317.80**	314.85	317.53	317.39
FFT	393	290.97	291.26	290.20	289.91	**291.62**
Tallying	393	284.25	285.83	284.38	284.19	**287.68**
Average (heuristics)	393	288.29	289.57	289.325	288.03	**290.68**
Overall average	393	298.01	300.89	300.27	299.46	**301.46**

Despite the significant performance improvements demonstrated by the proposed model, it lacks a specific mechanism for handling adversarial samples. Adversarial examples are generated by introducing small perturbations to normal data points, which remain correctly recognizable to humans but are misclassified by prediction models (Kwon, 2023; Kwon and Kim, 2023). Given the potential application of this model for automating critical human decisions, such as detecting diseases or forest fires, it is crucial for the model to be resilient to adversarial attacks. Several mitigation strategies, including adversarial training and transfer learning, have been developed to address this issue (Kwon and Lee, 2022; Kwon et al., 2022). Incorporating such mitigation strategies into the proposed model presents a promising direction for future work.

## 5 Conclusion: active learning and oracle uncertainty

AL algorithms hold tremendous potential but should be based on realistic assumptions. Starting from the commonsense observation that sometimes the labels necessary for AL must be provided by a human, who might be biased, we model the oracle by fast-and-frugal heuristics. In other words, we also modeled the labeling strategy used by an oracle beyond the known modeling of data and prediction uncertainty in active learning. Our study showed the need to design AL algorithms robust to labeling bias, and this was pursued by taking inspiration from heuristics research. More generally, this exercise shows that it may be beneficial to consider human psychology in the design of active learning algorithms.

## Data Availability

The original contributions presented in the study are included in the article/Supplementary material. The codeset required to replicate this study is available at https://github.com/SriramML/AL-with-Human-Heuristics.git. Further inquiries can be directed to the corresponding author.
