# Supplementary figures and images for: Active learning with human heuristics: an algorithm robust to labeling bias
Source: Front Artif Intell. 2024 Nov 19;7:1491932. doi: 10.3389/frai.2024.1491932 (PMC11611880; doi:10.3389/frai.2024.1491932)

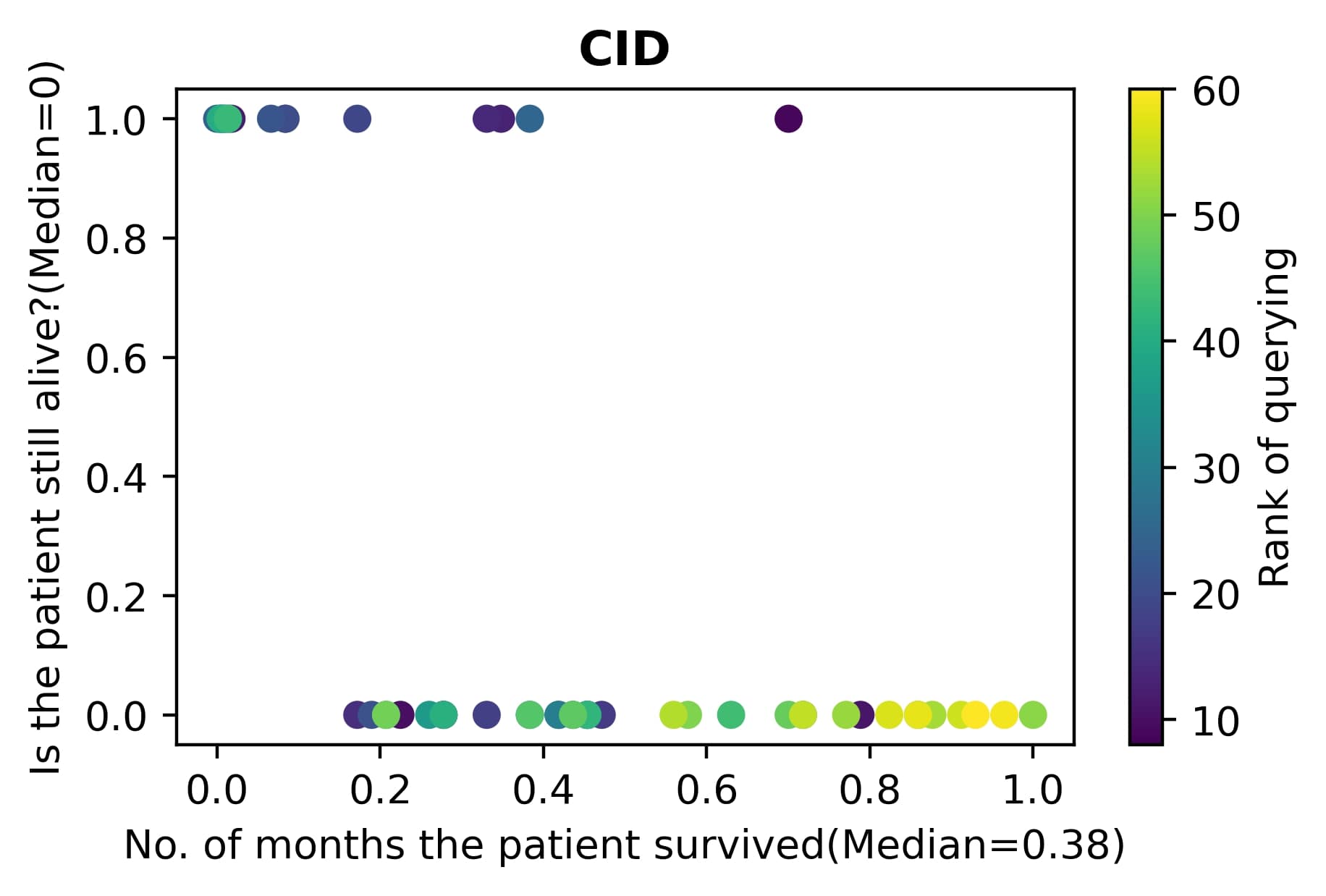

Supplement: Supplementary file 1 [file Data_Sheet_1.zip › Figures in Supplimentary Material/CID_Figure_10.jpg]

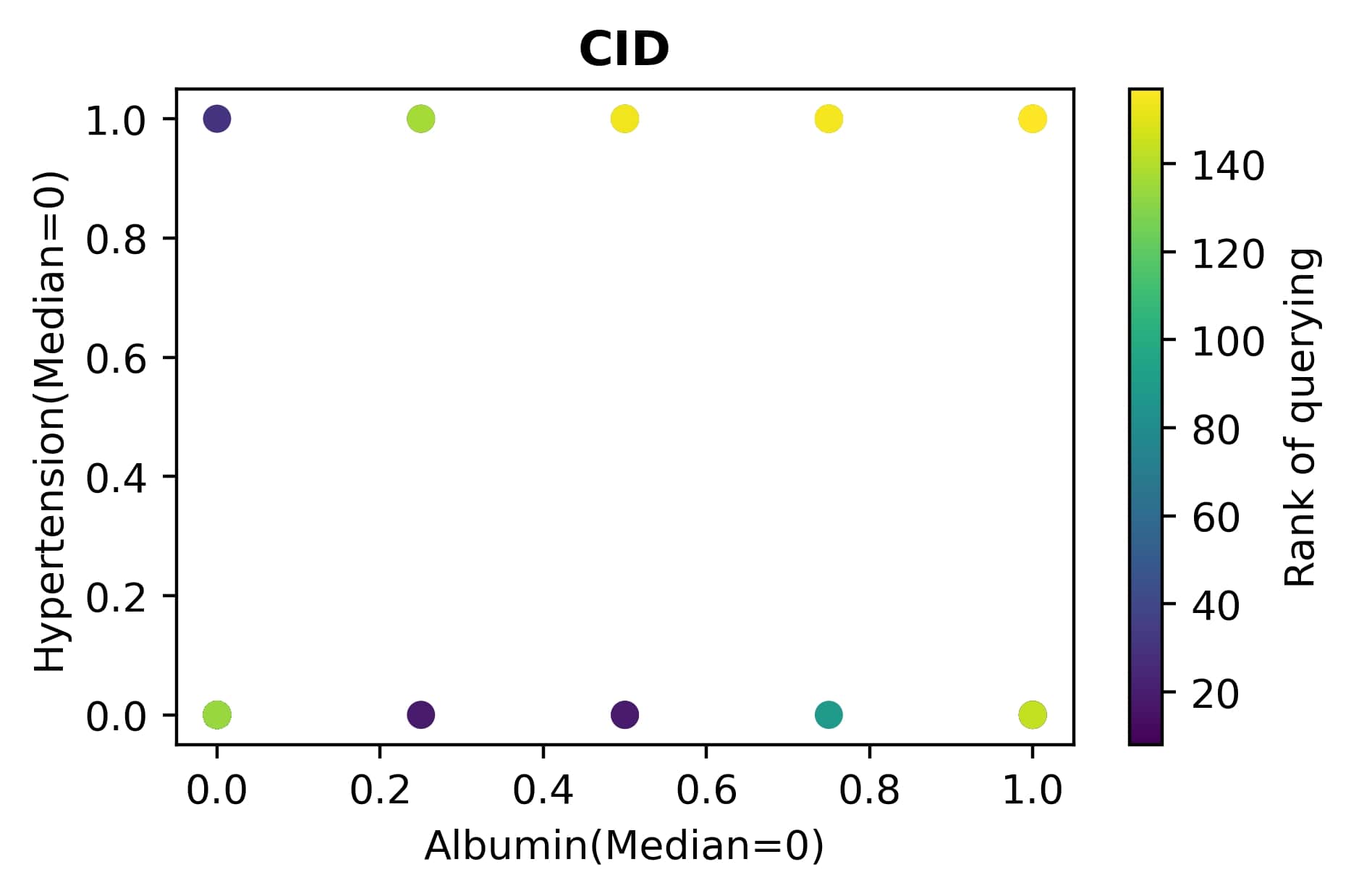

Supplement: Supplementary file 1 [file Data_Sheet_1.zip › Figures in Supplimentary Material/CID_Figure_11.jpg]

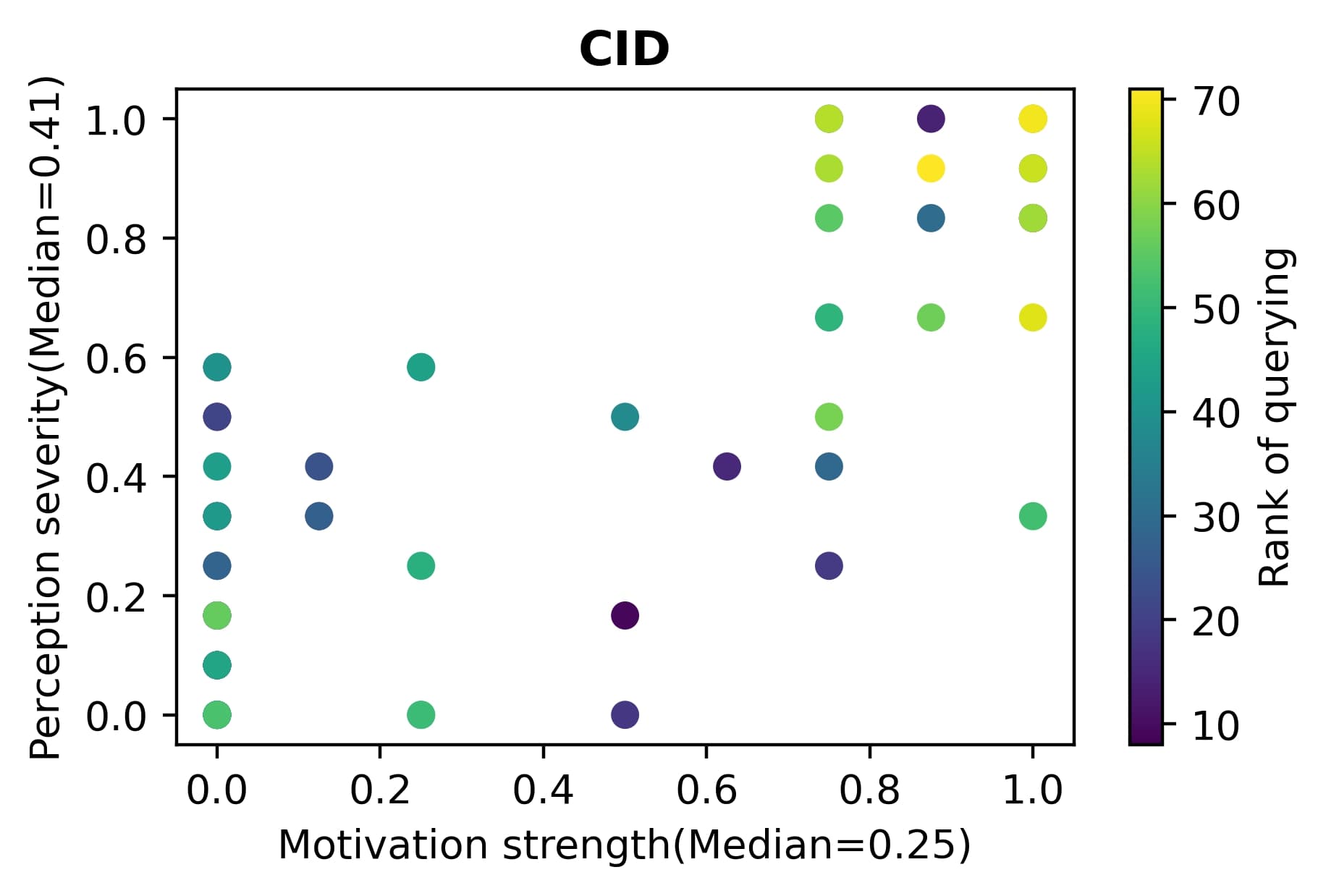

Supplement: Supplementary file 1 [file Data_Sheet_1.zip › Figures in Supplimentary Material/CID_Figure_12.jpg]

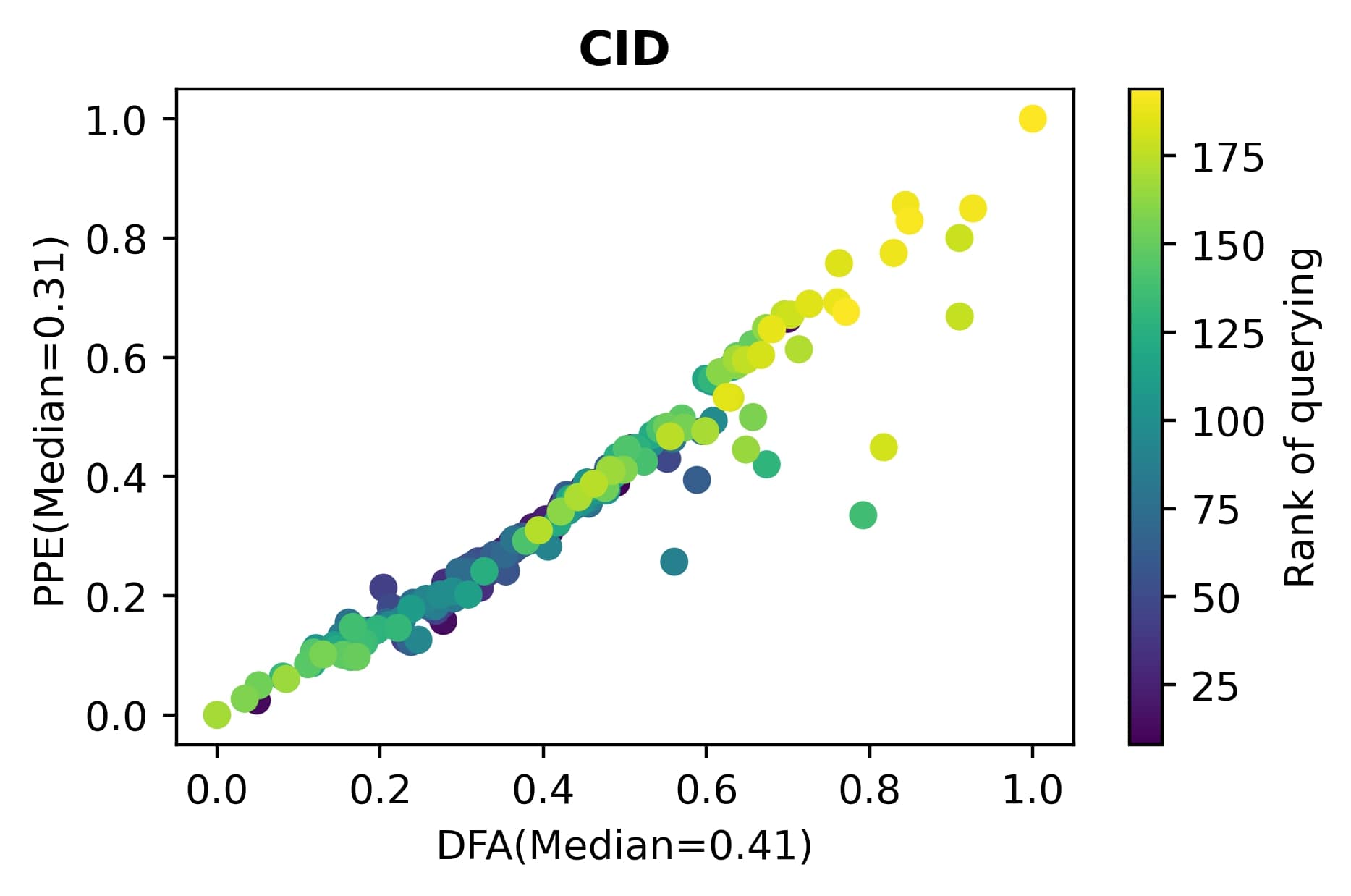

Supplement: Supplementary file 1 [file Data_Sheet_1.zip › Figures in Supplimentary Material/CID_Figure_13.jpg]

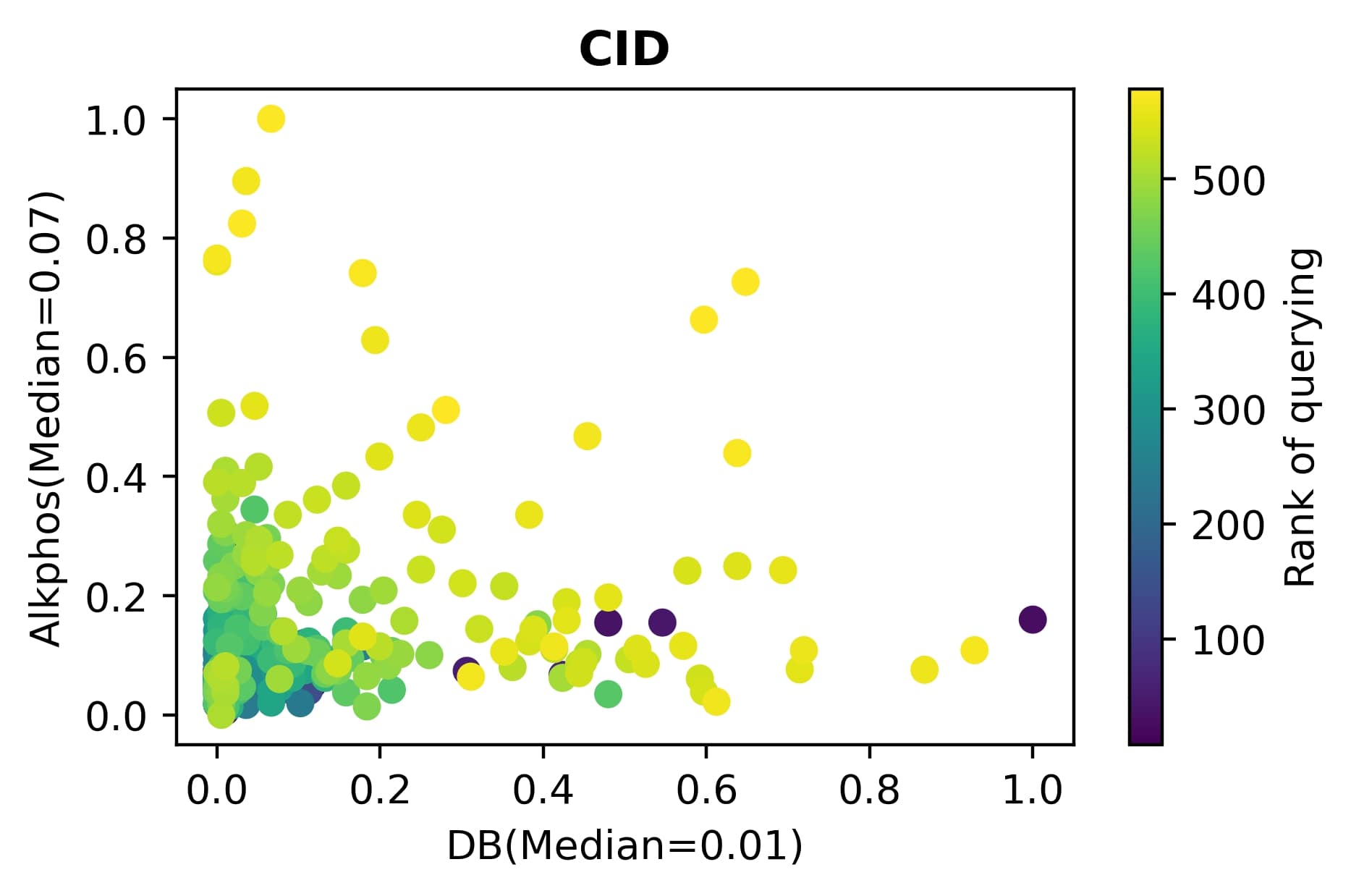

Supplement: Supplementary file 1 [file Data_Sheet_1.zip › Figures in Supplimentary Material/CID_Figure_14.jpg]

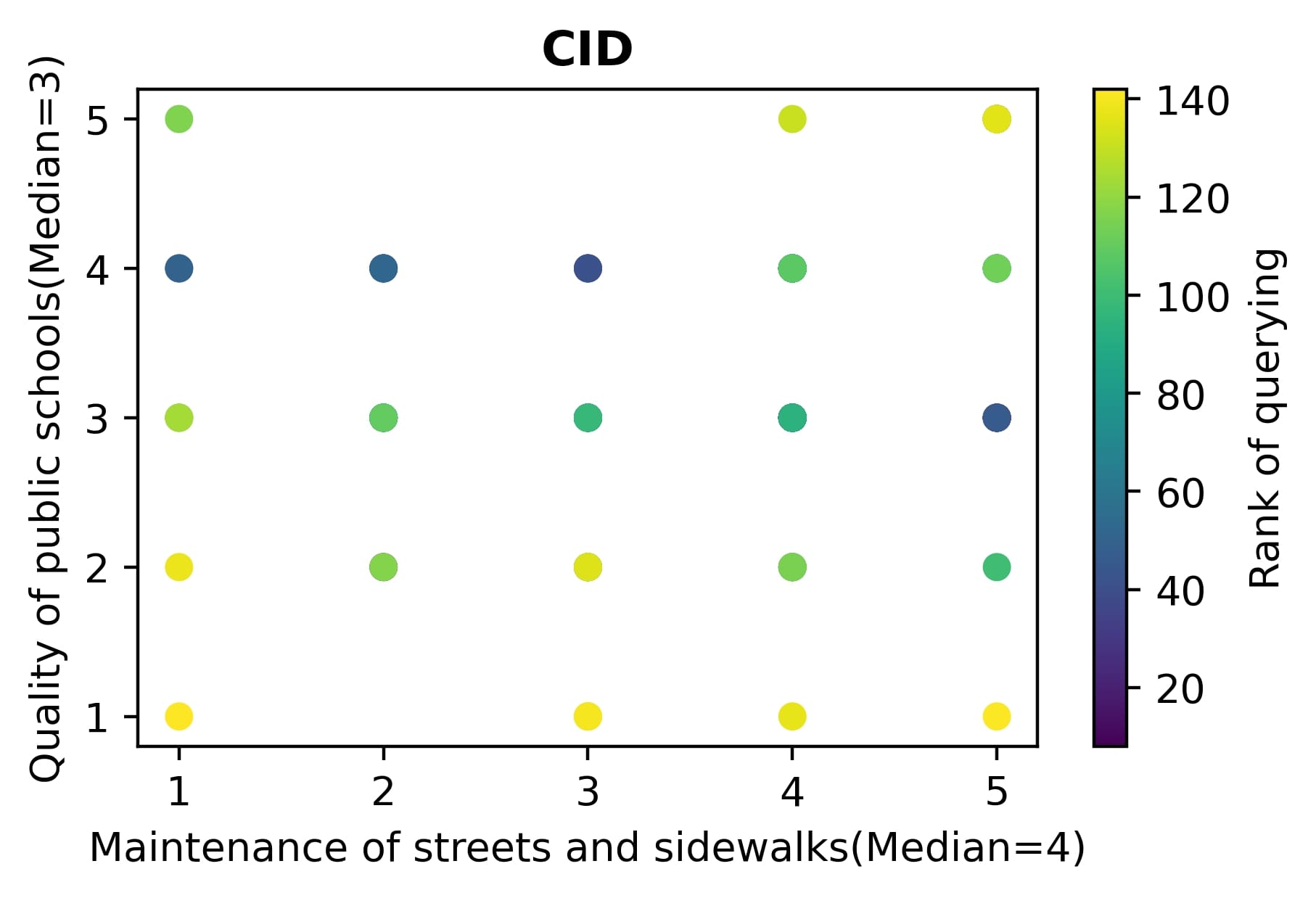

Supplement: Supplementary file 1 [file Data_Sheet_1.zip › Figures in Supplimentary Material/CID_Figure_15.jpg]

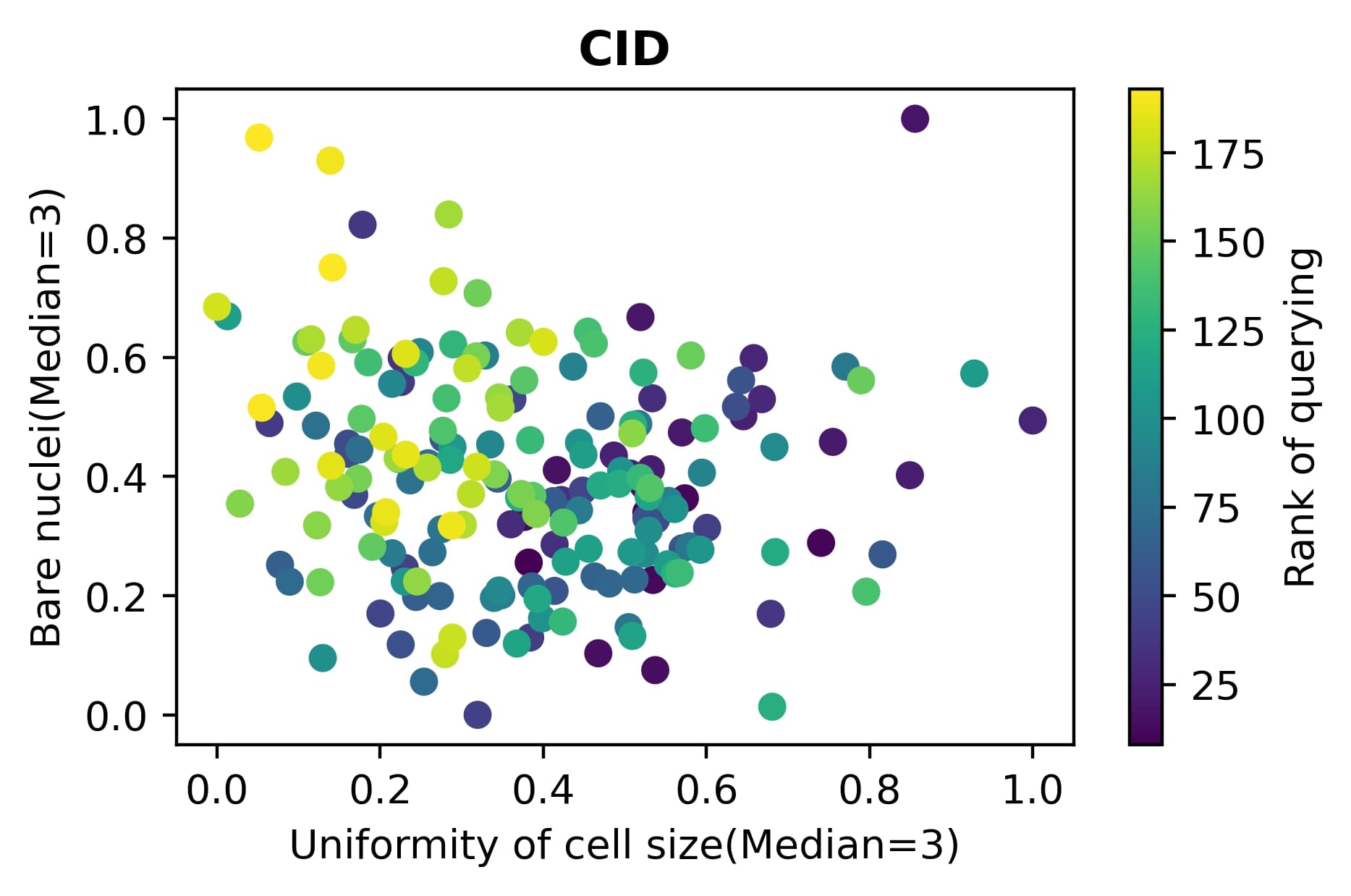

Supplement: Supplementary file 1 [file Data_Sheet_1.zip › Figures in Supplimentary Material/CID_Figure_16.jpg]

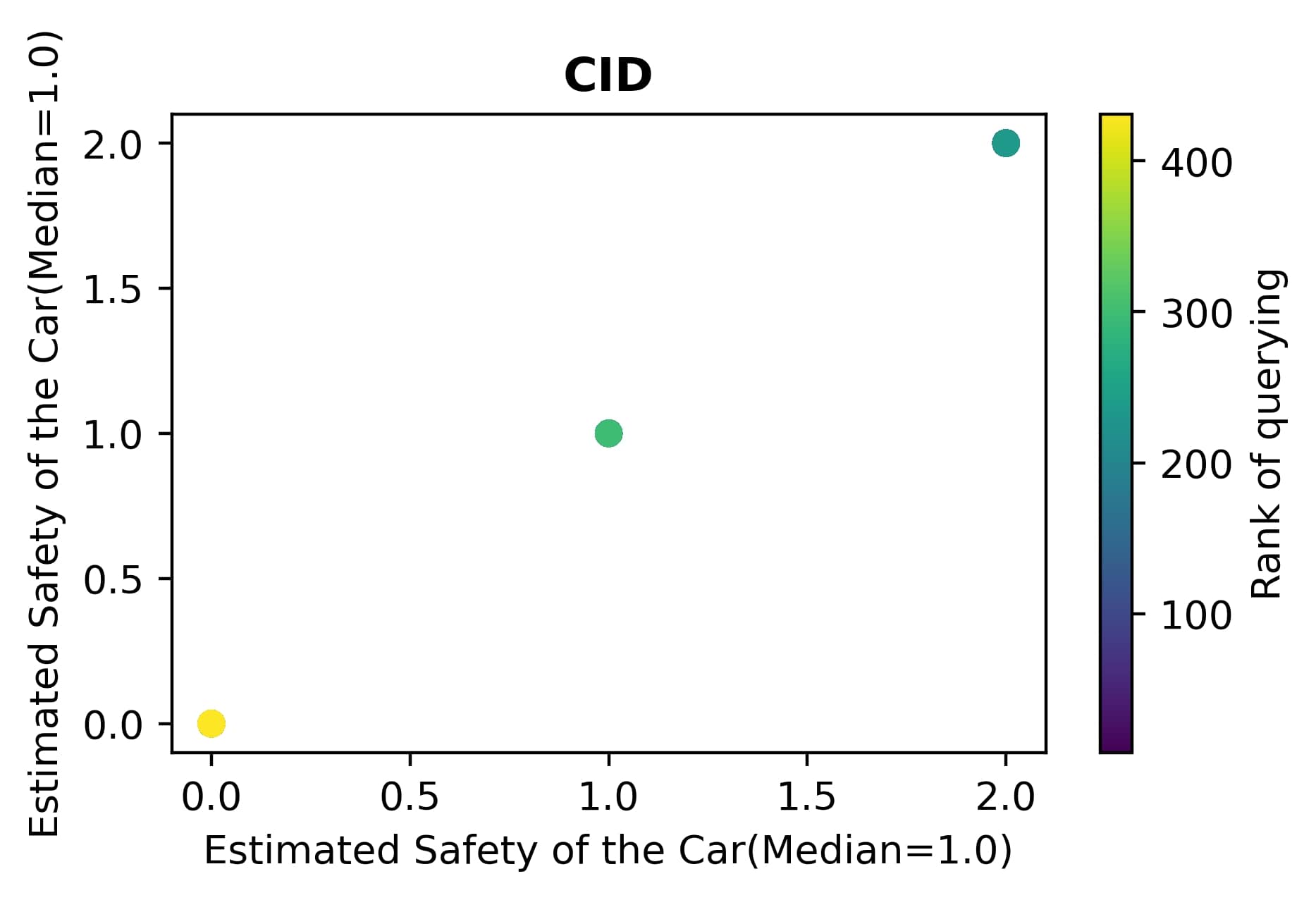

Supplement: Supplementary file 1 [file Data_Sheet_1.zip › Figures in Supplimentary Material/CID_Figure_3.jpg]

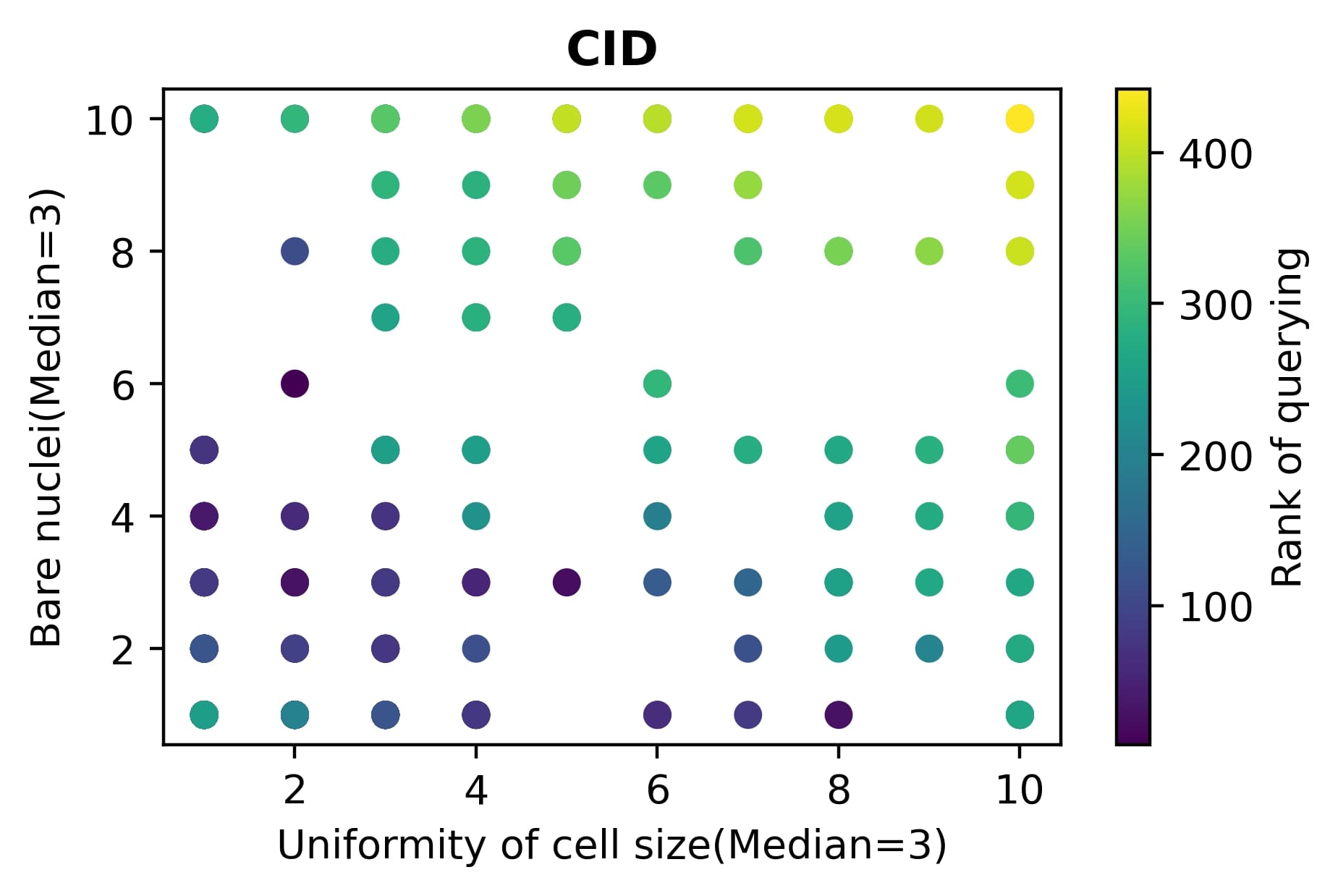

Supplement: Supplementary file 1 [file Data_Sheet_1.zip › Figures in Supplimentary Material/CID_Figure_4.jpg]

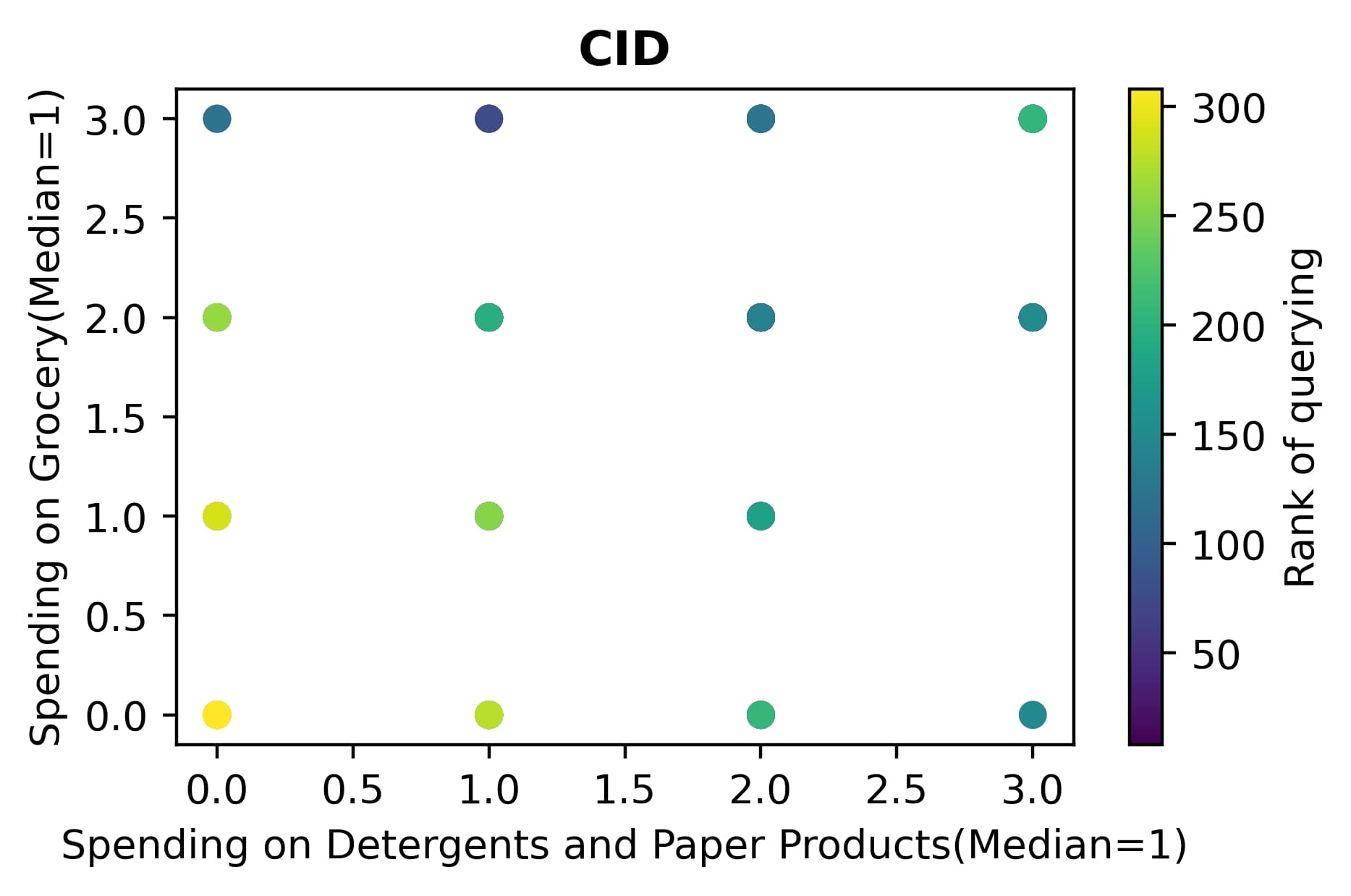

Supplement: Supplementary file 1 [file Data_Sheet_1.zip › Figures in Supplimentary Material/CID_Figure_5.jpg]

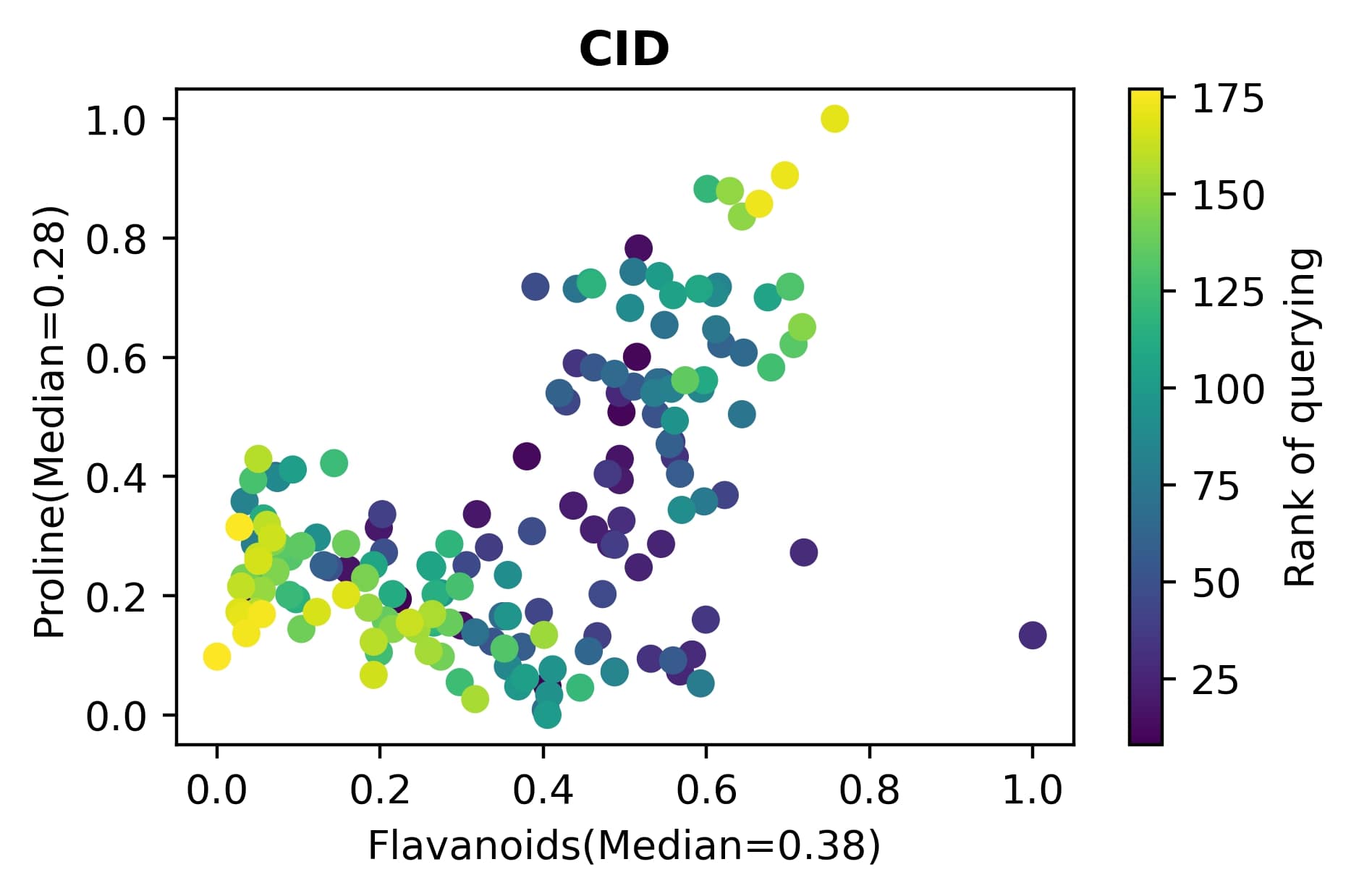

Supplement: Supplementary file 1 [file Data_Sheet_1.zip › Figures in Supplimentary Material/CID_Figure_6.jpg]

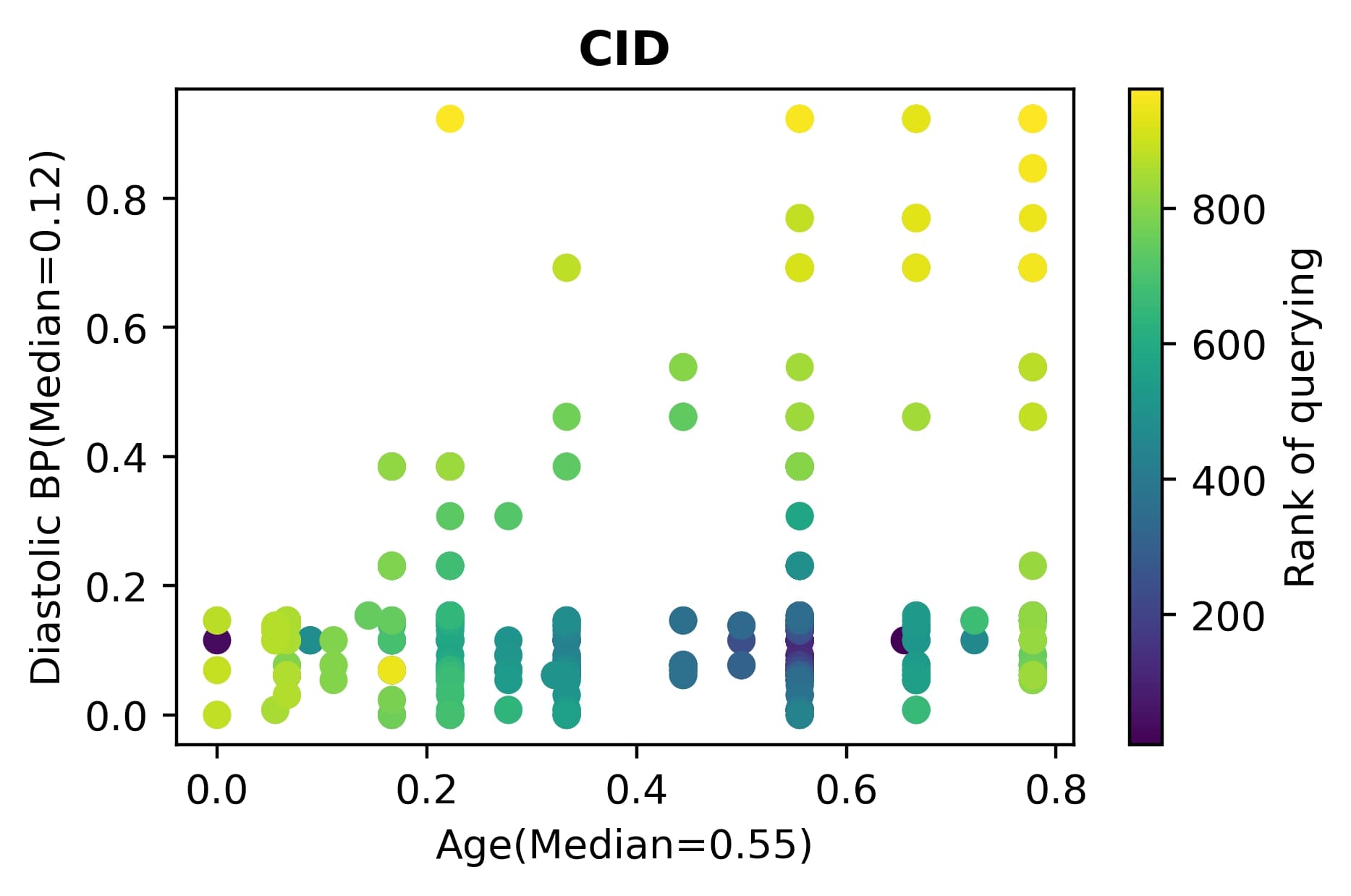

Supplement: Supplementary file 1 [file Data_Sheet_1.zip › Figures in Supplimentary Material/CID_Figure_7.jpg]

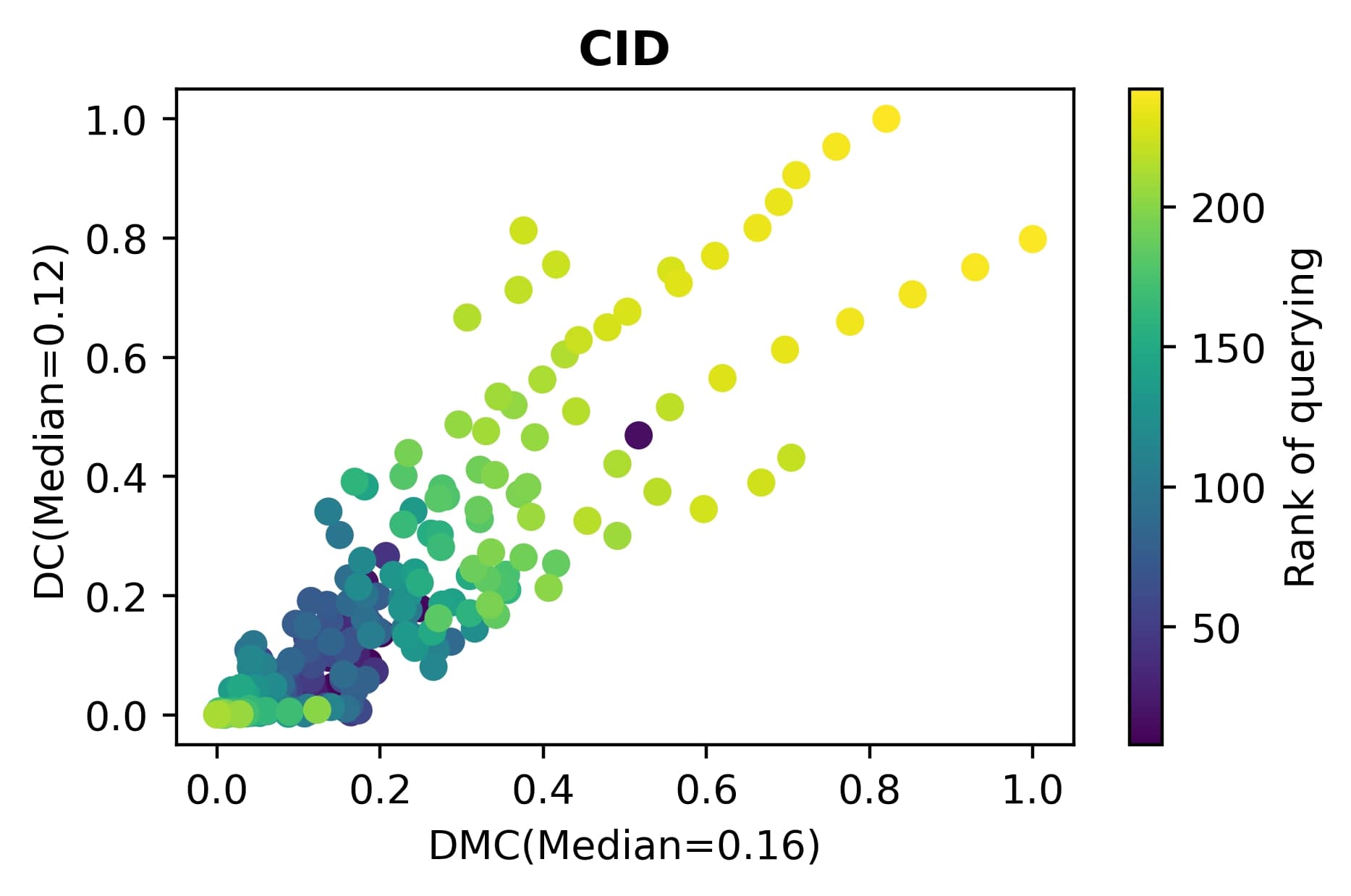

Supplement: Supplementary file 1 [file Data_Sheet_1.zip › Figures in Supplimentary Material/CID_Figure_8.jpg]

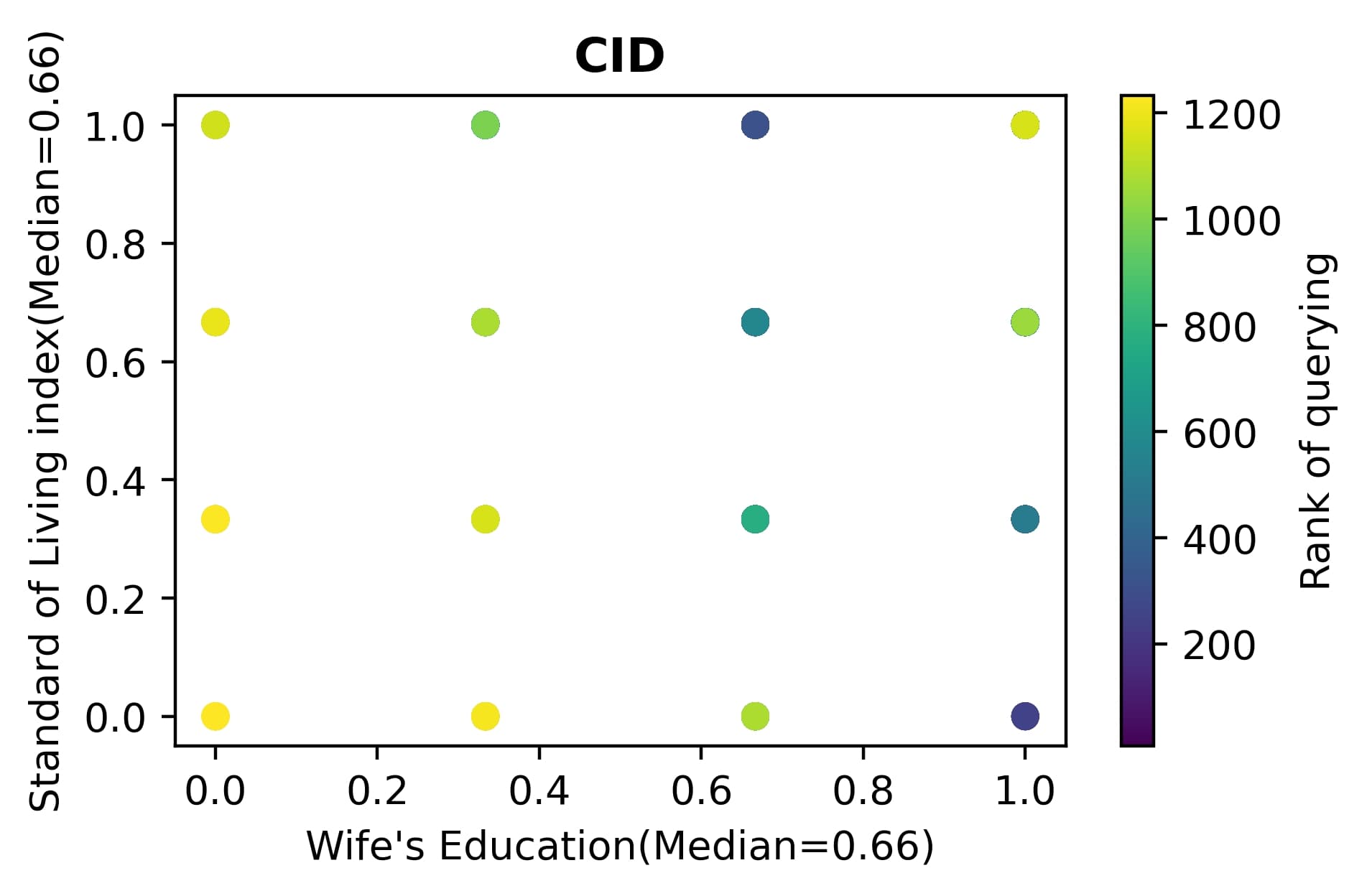

Supplement: Supplementary file 1 [file Data_Sheet_1.zip › Figures in Supplimentary Material/CID_Figure_9.jpg]

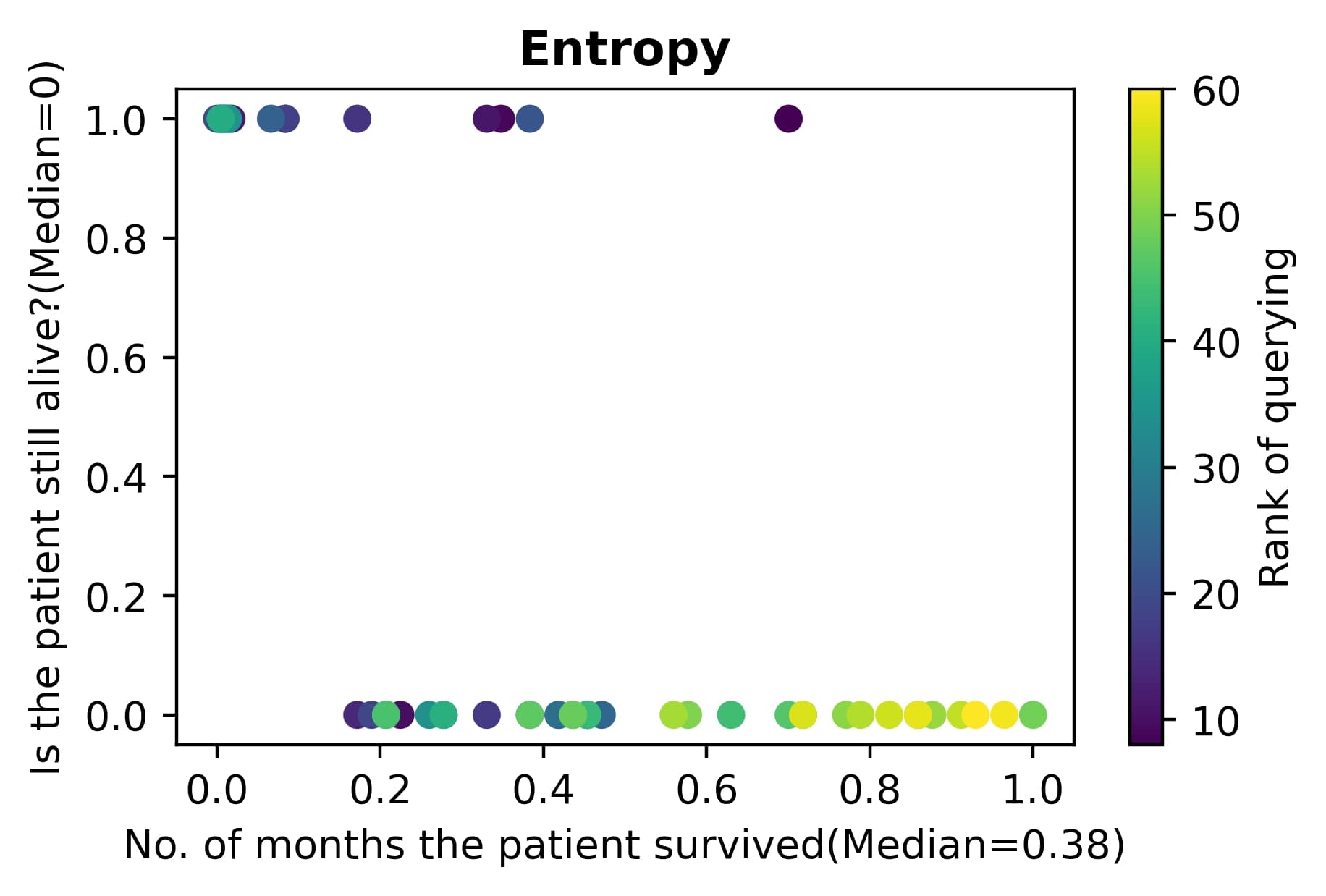

Supplement: Supplementary file 1 [file Data_Sheet_1.zip › Figures in Supplimentary Material/Entropy_Figure_10.jpg]

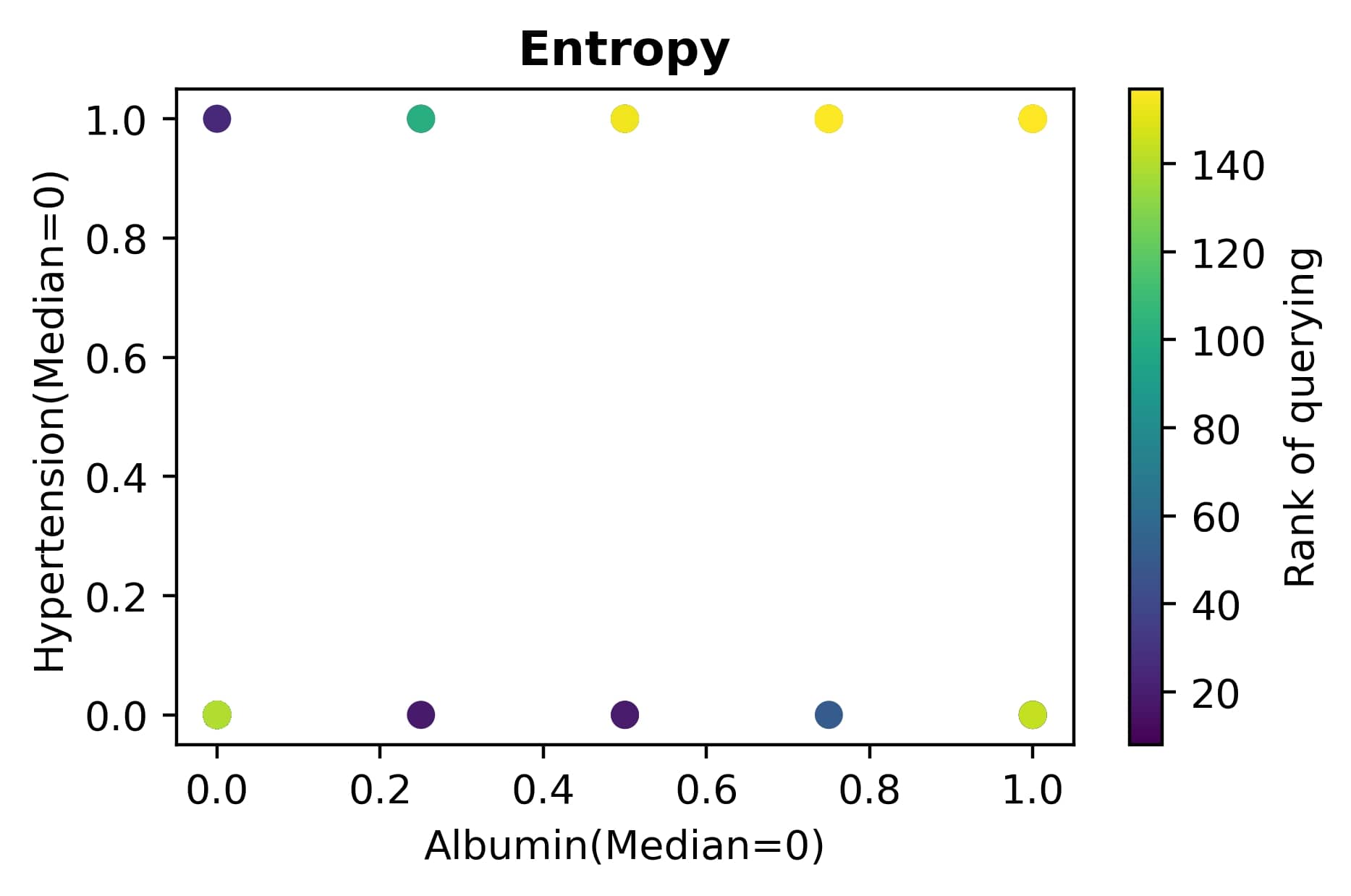

Supplement: Supplementary file 1 [file Data_Sheet_1.zip › Figures in Supplimentary Material/Entropy_Figure_11.jpg]

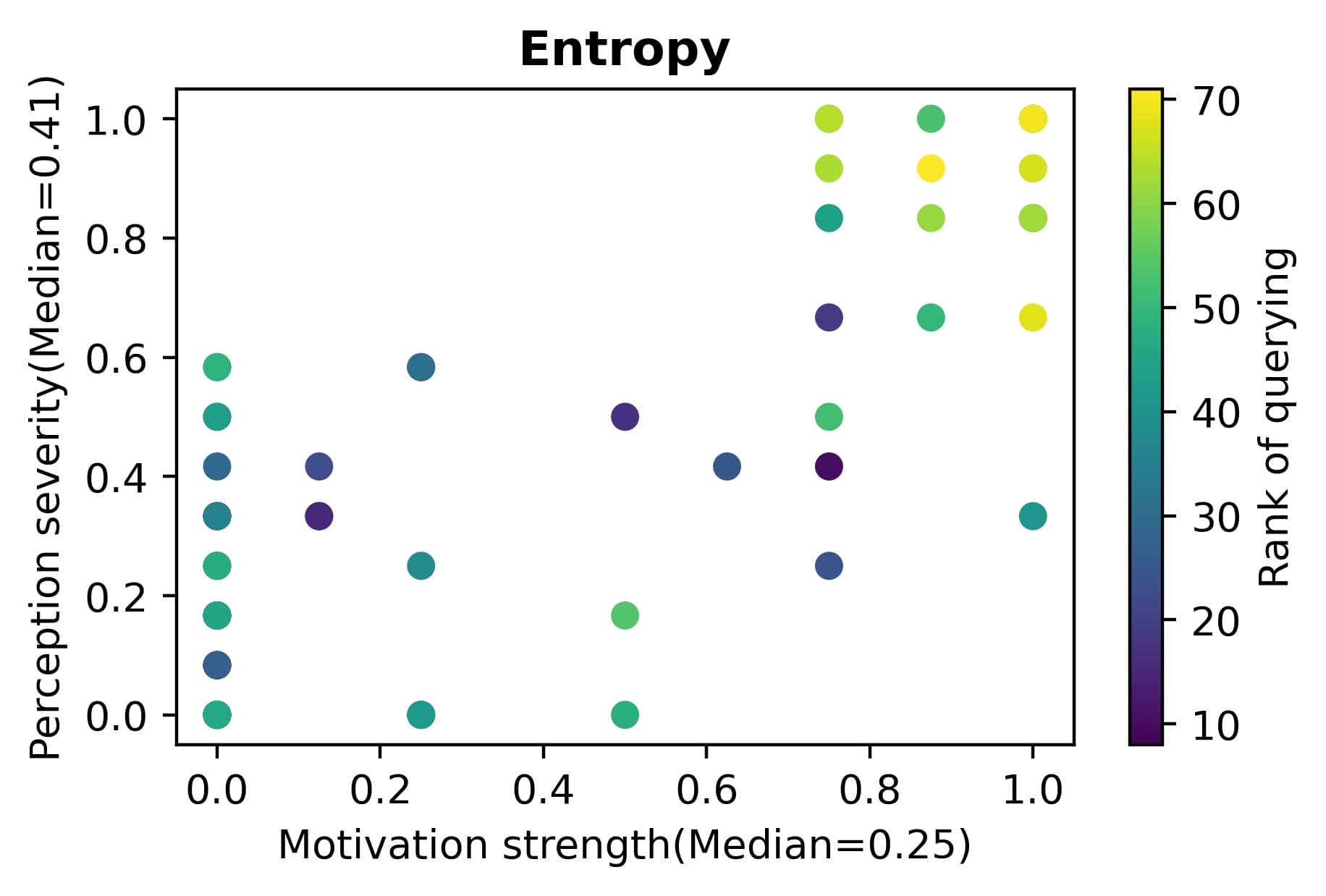

Supplement: Supplementary file 1 [file Data_Sheet_1.zip › Figures in Supplimentary Material/Entropy_Figure_12.jpg]

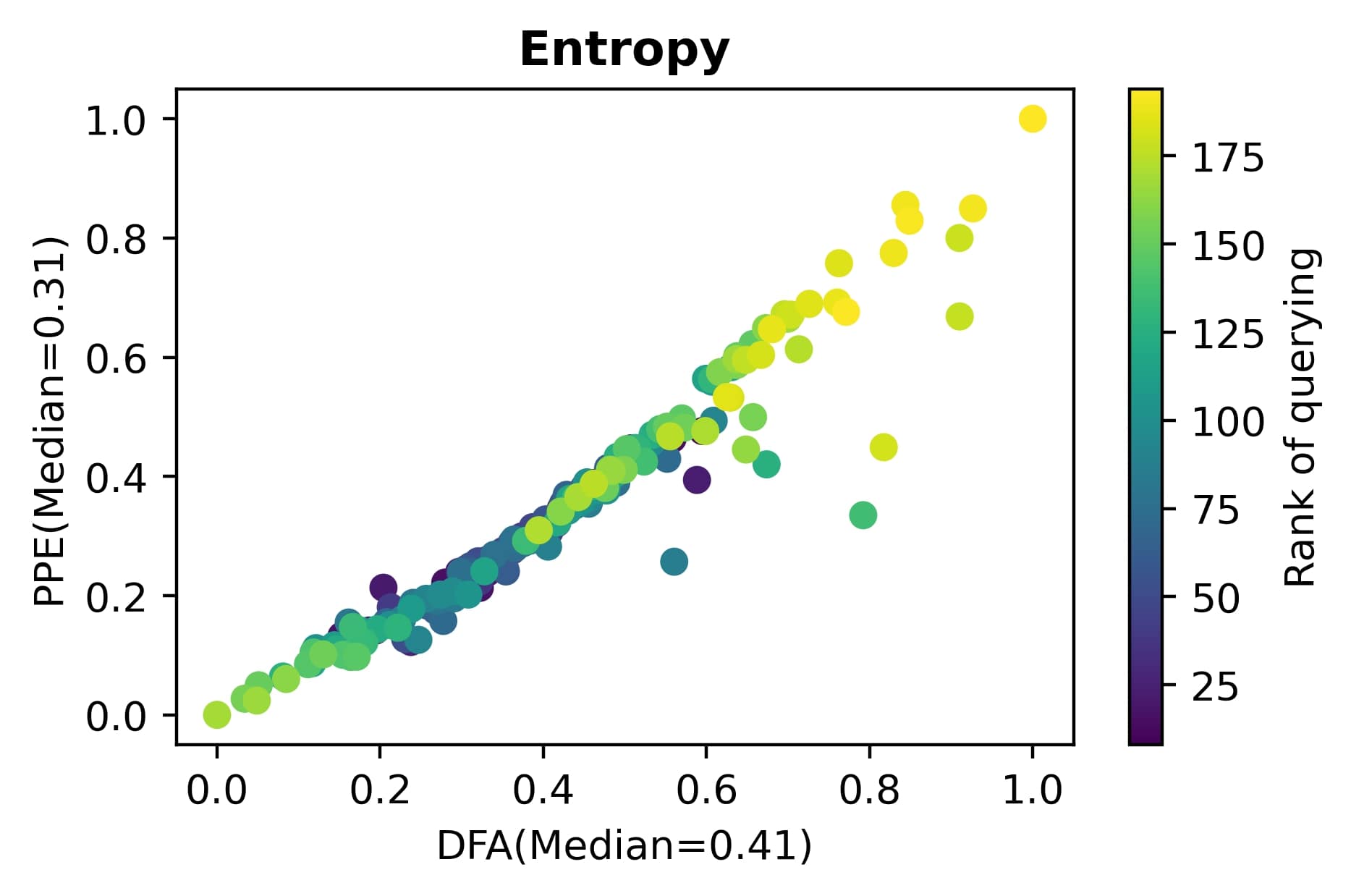

Supplement: Supplementary file 1 [file Data_Sheet_1.zip › Figures in Supplimentary Material/Entropy_Figure_13.jpg]

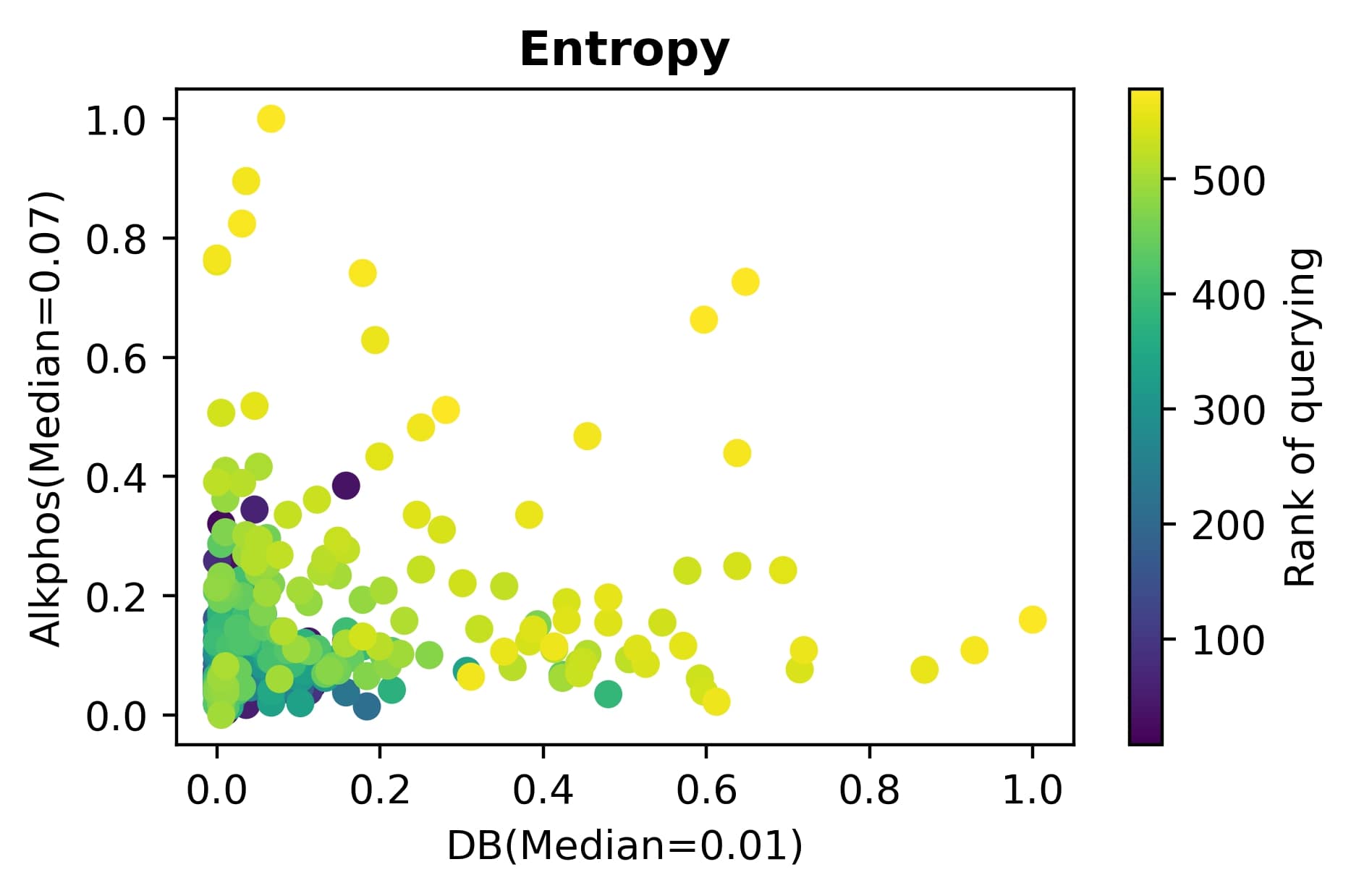

Supplement: Supplementary file 1 [file Data_Sheet_1.zip › Figures in Supplimentary Material/Entropy_Figure_14.jpg]

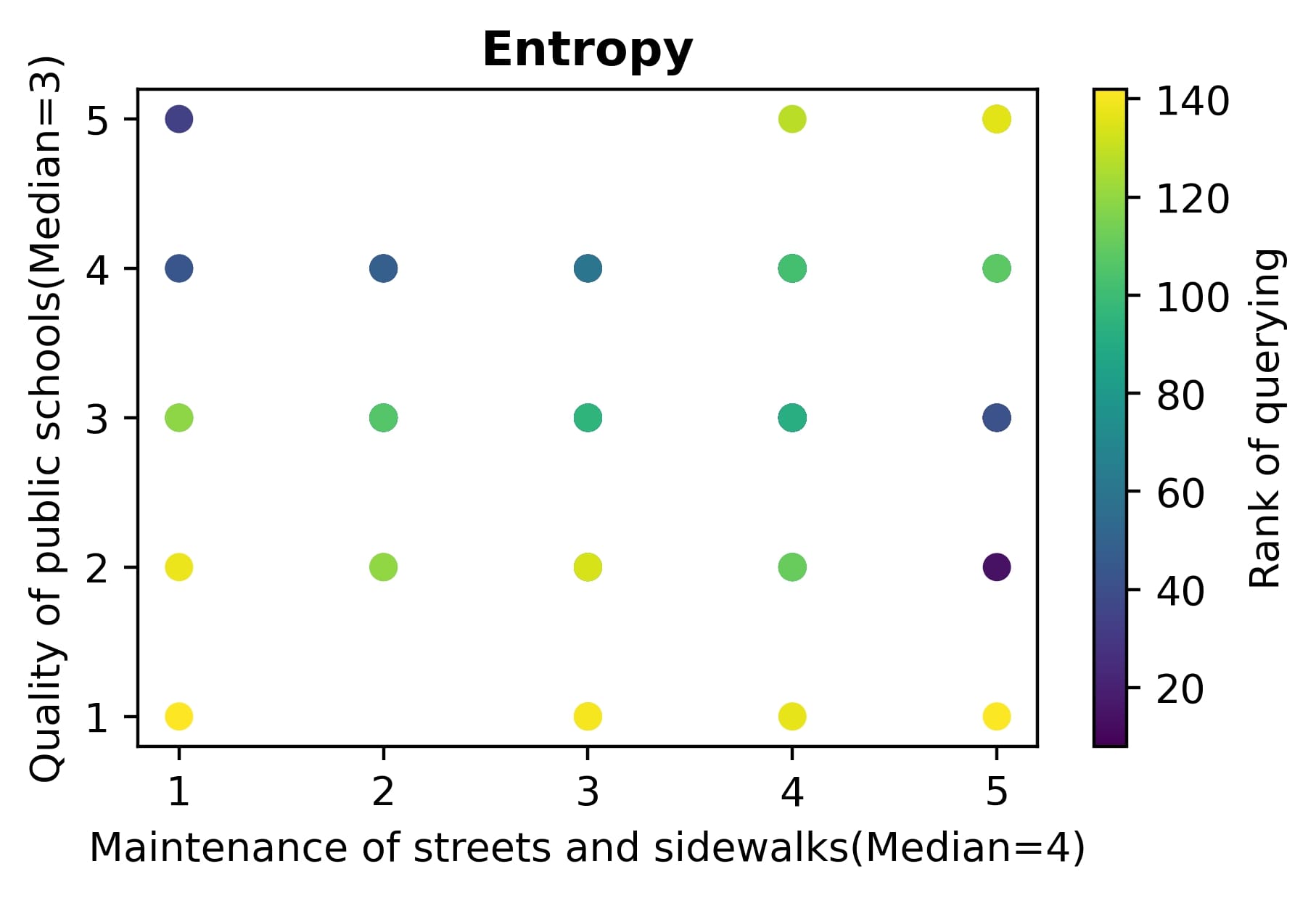

Supplement: Supplementary file 1 [file Data_Sheet_1.zip › Figures in Supplimentary Material/Entropy_Figure_15.jpg]

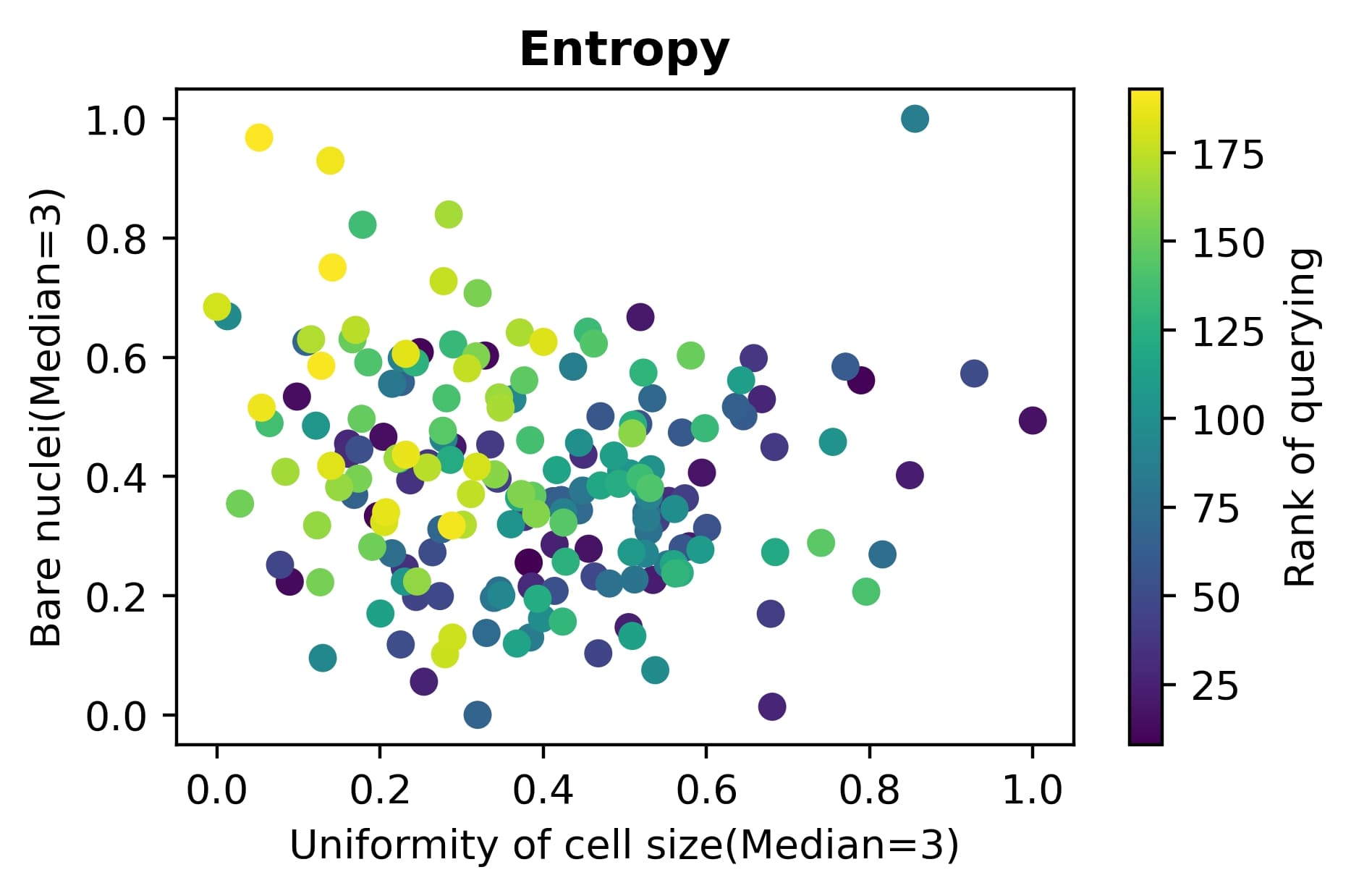

Supplement: Supplementary file 1 [file Data_Sheet_1.zip › Figures in Supplimentary Material/Entropy_Figure_16.jpg]

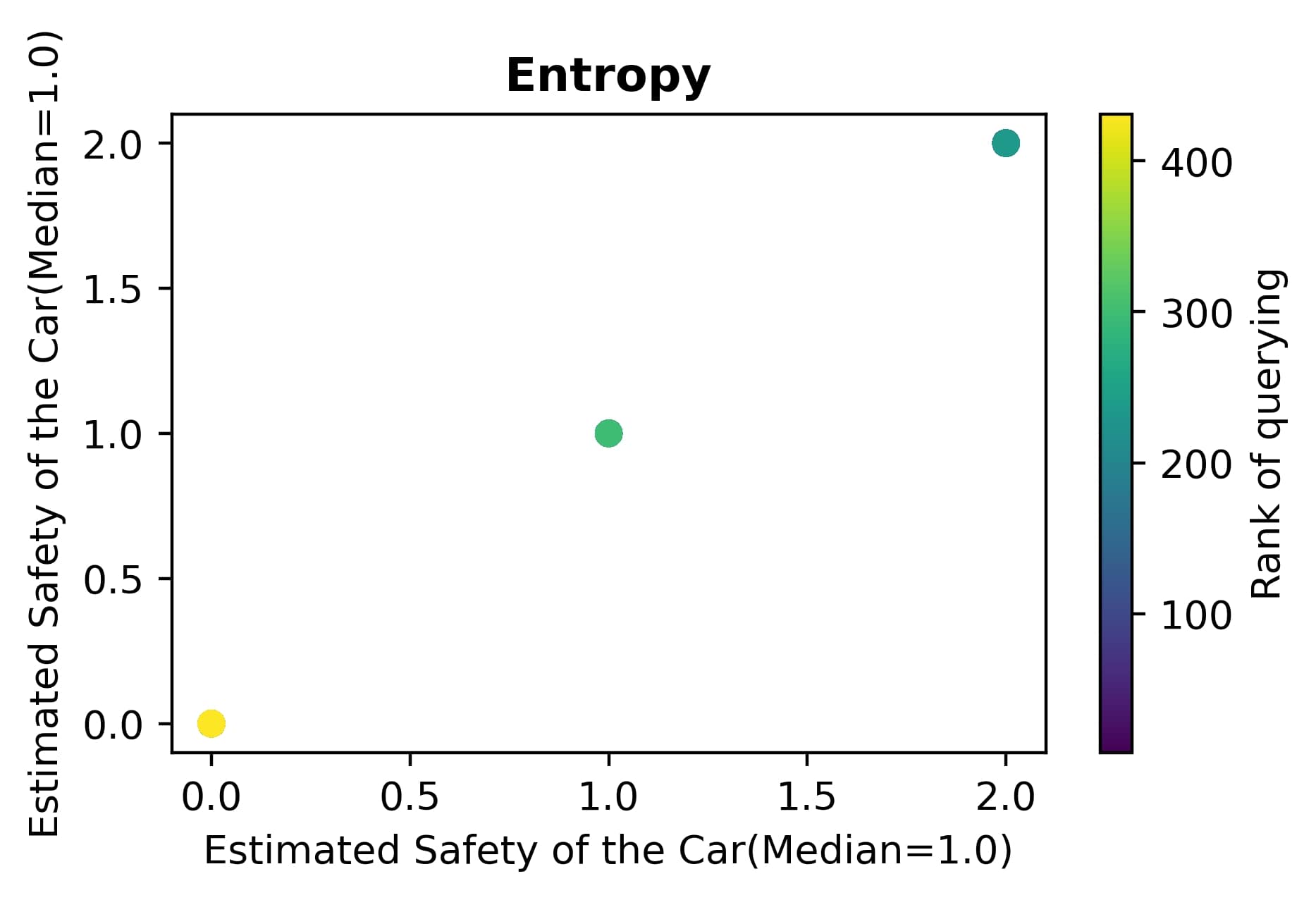

Supplement: Supplementary file 1 [file Data_Sheet_1.zip › Figures in Supplimentary Material/Entropy_Figure_3.jpg]

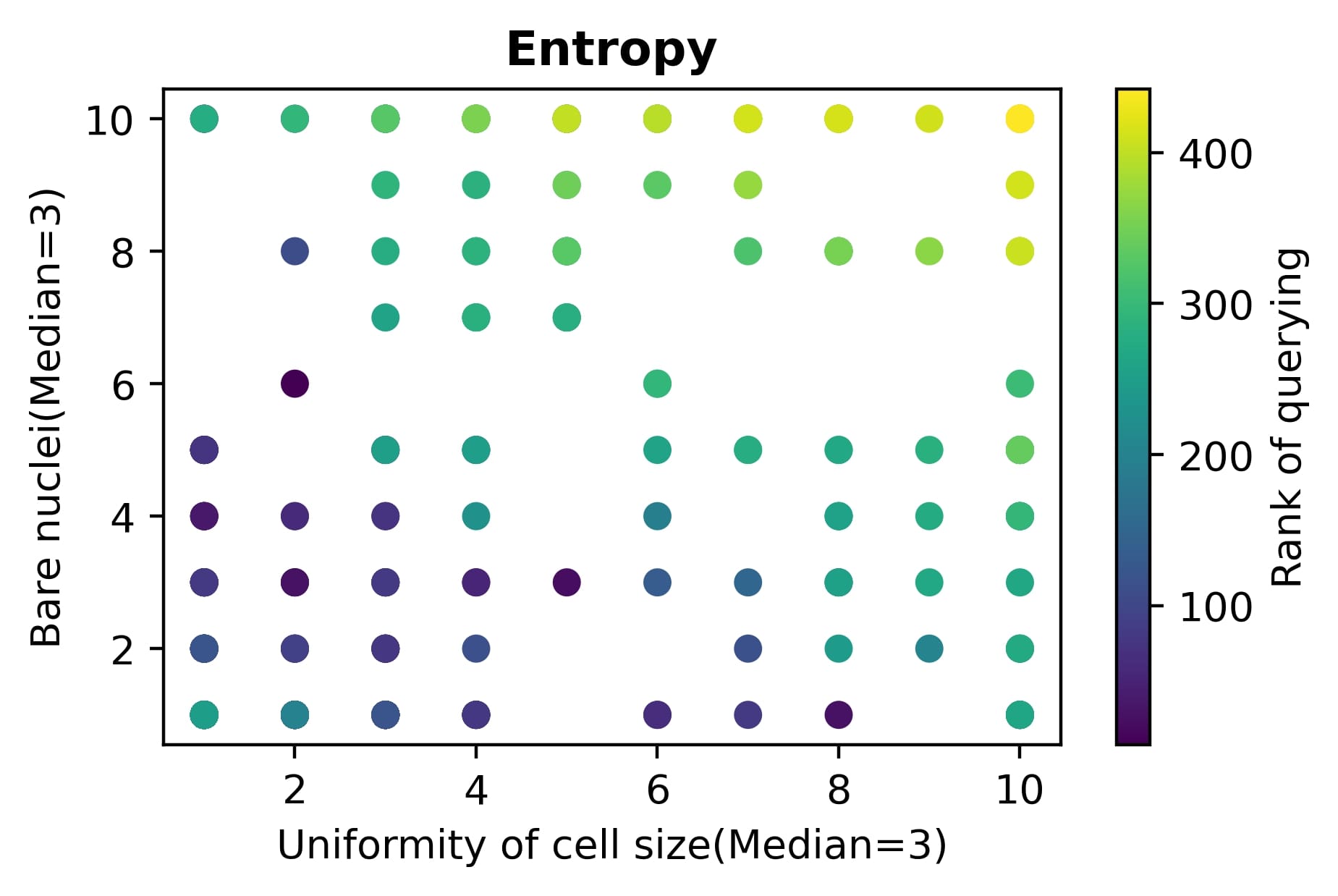

Supplement: Supplementary file 1 [file Data_Sheet_1.zip › Figures in Supplimentary Material/Entropy_Figure_4.jpg]

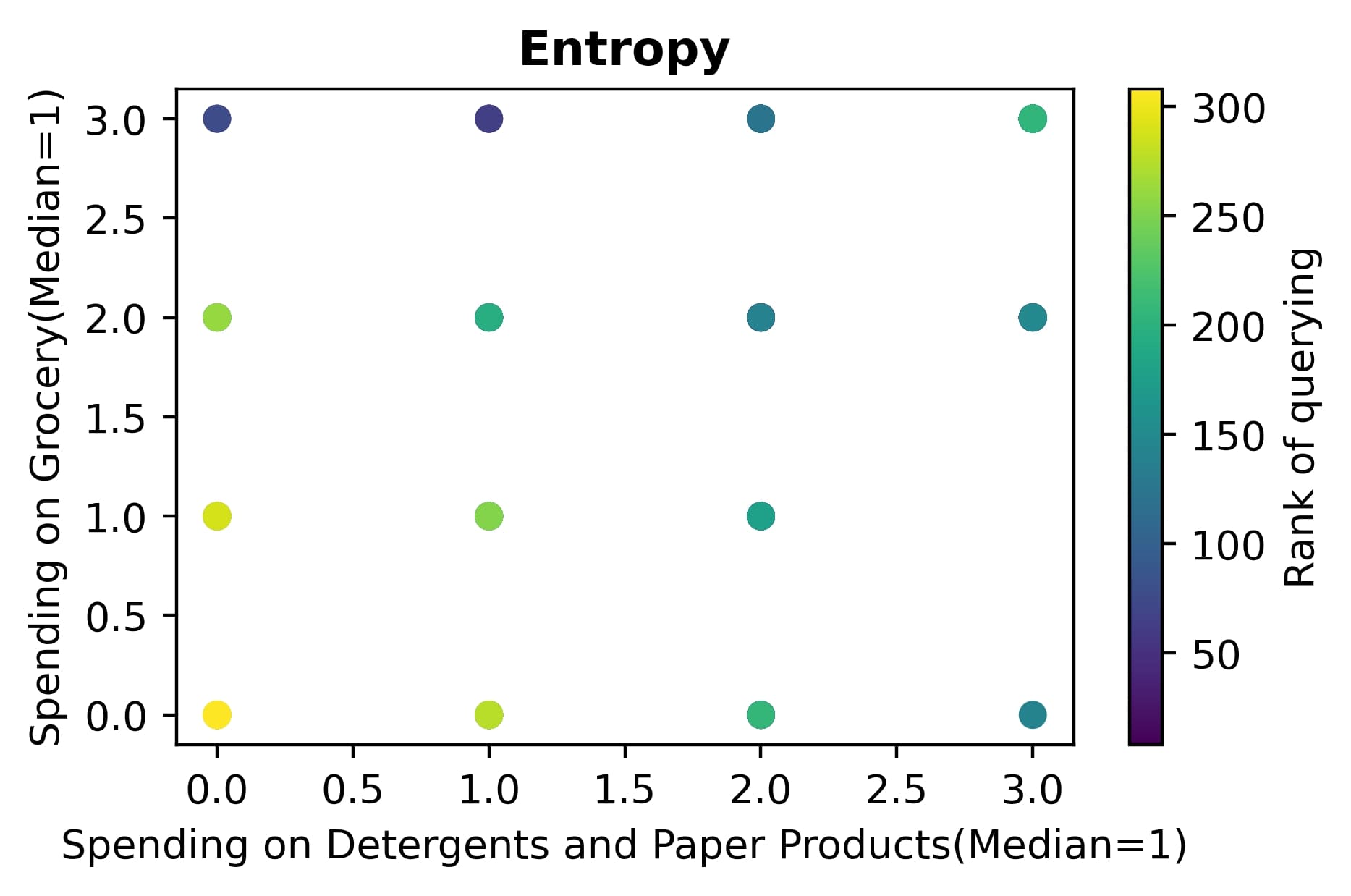

Supplement: Supplementary file 1 [file Data_Sheet_1.zip › Figures in Supplimentary Material/Entropy_Figure_5.jpg]

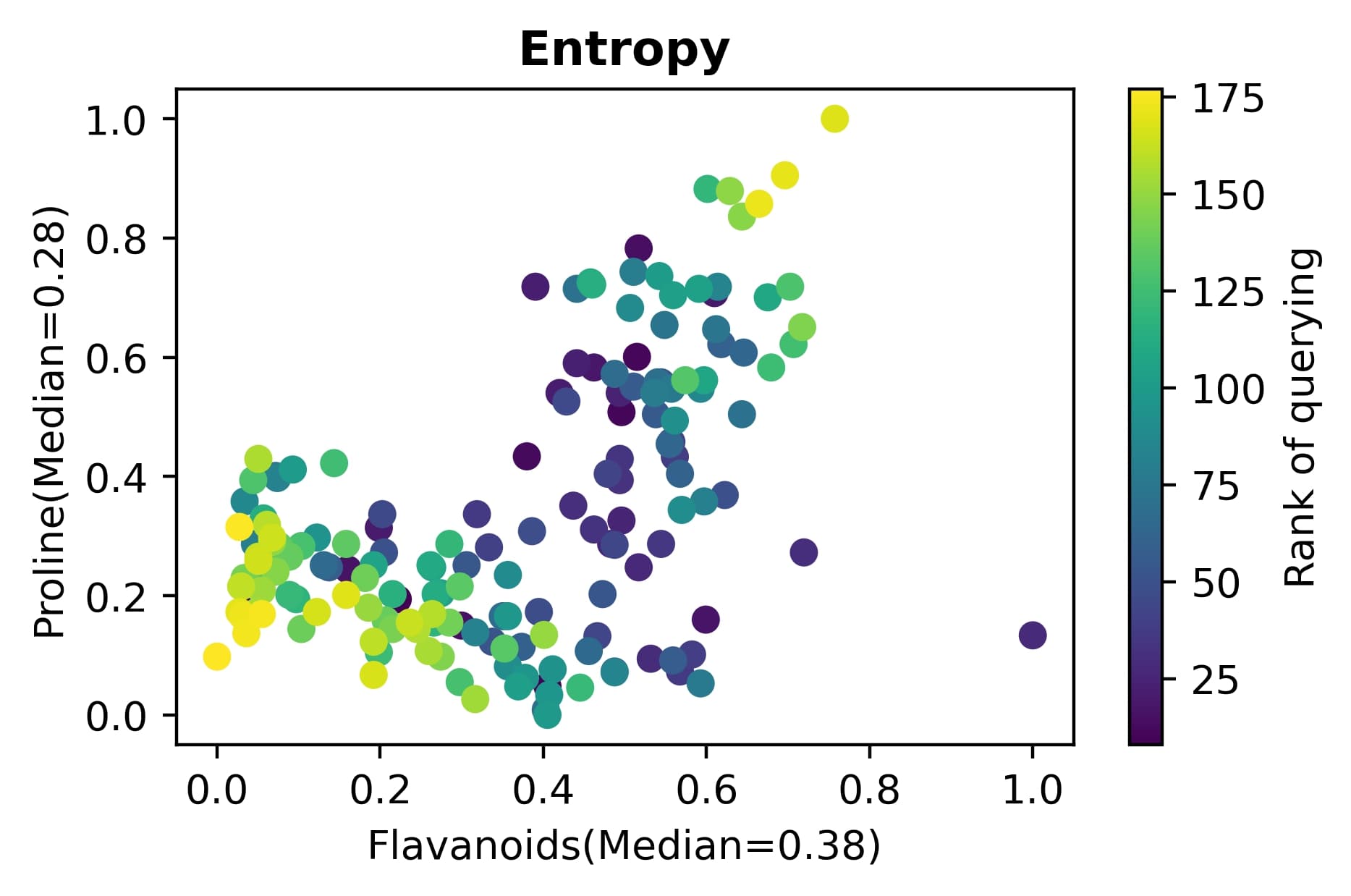

Supplement: Supplementary file 1 [file Data_Sheet_1.zip › Figures in Supplimentary Material/Entropy_Figure_6.jpg]

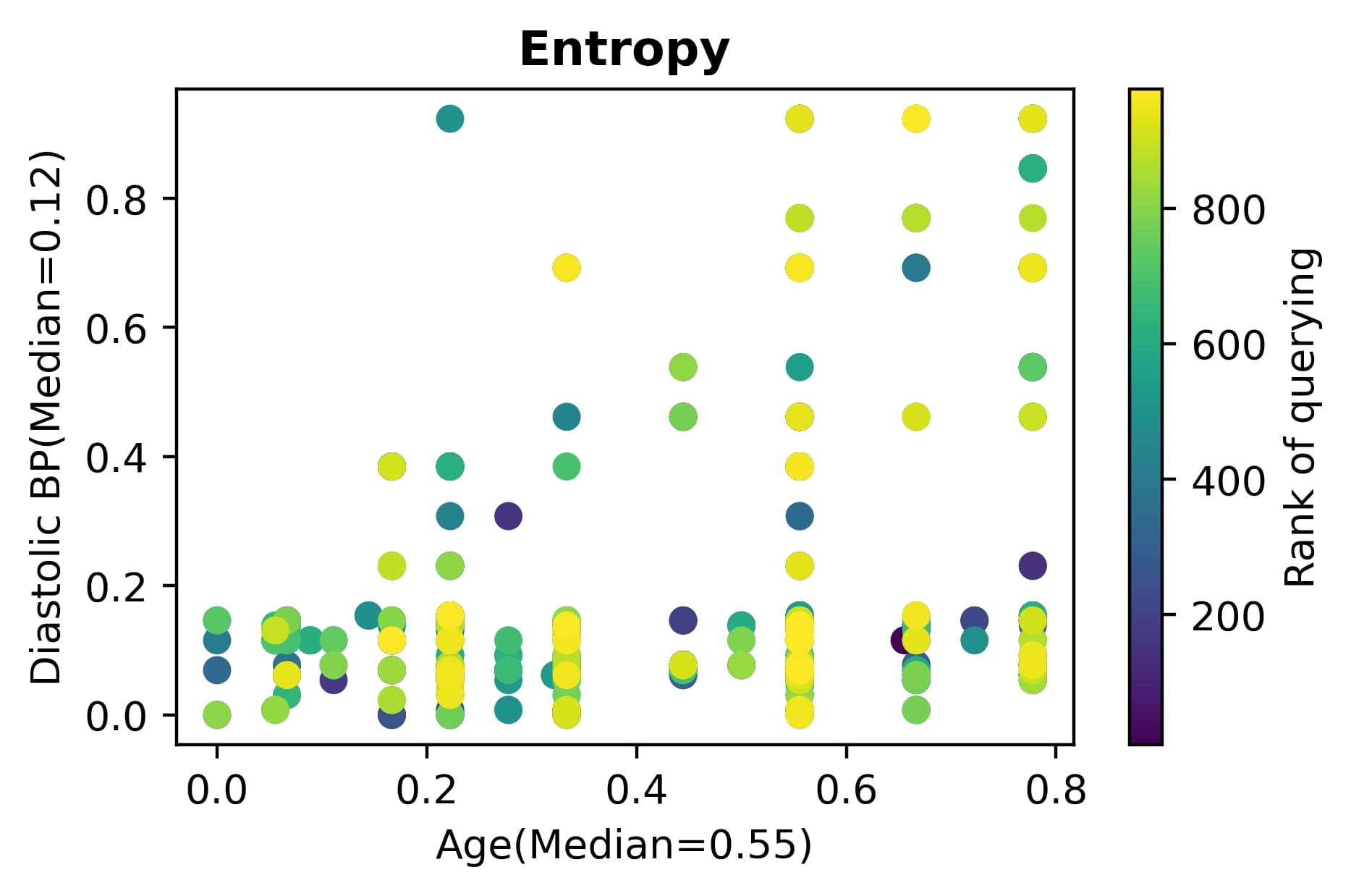

Supplement: Supplementary file 1 [file Data_Sheet_1.zip › Figures in Supplimentary Material/Entropy_Figure_7.jpg]

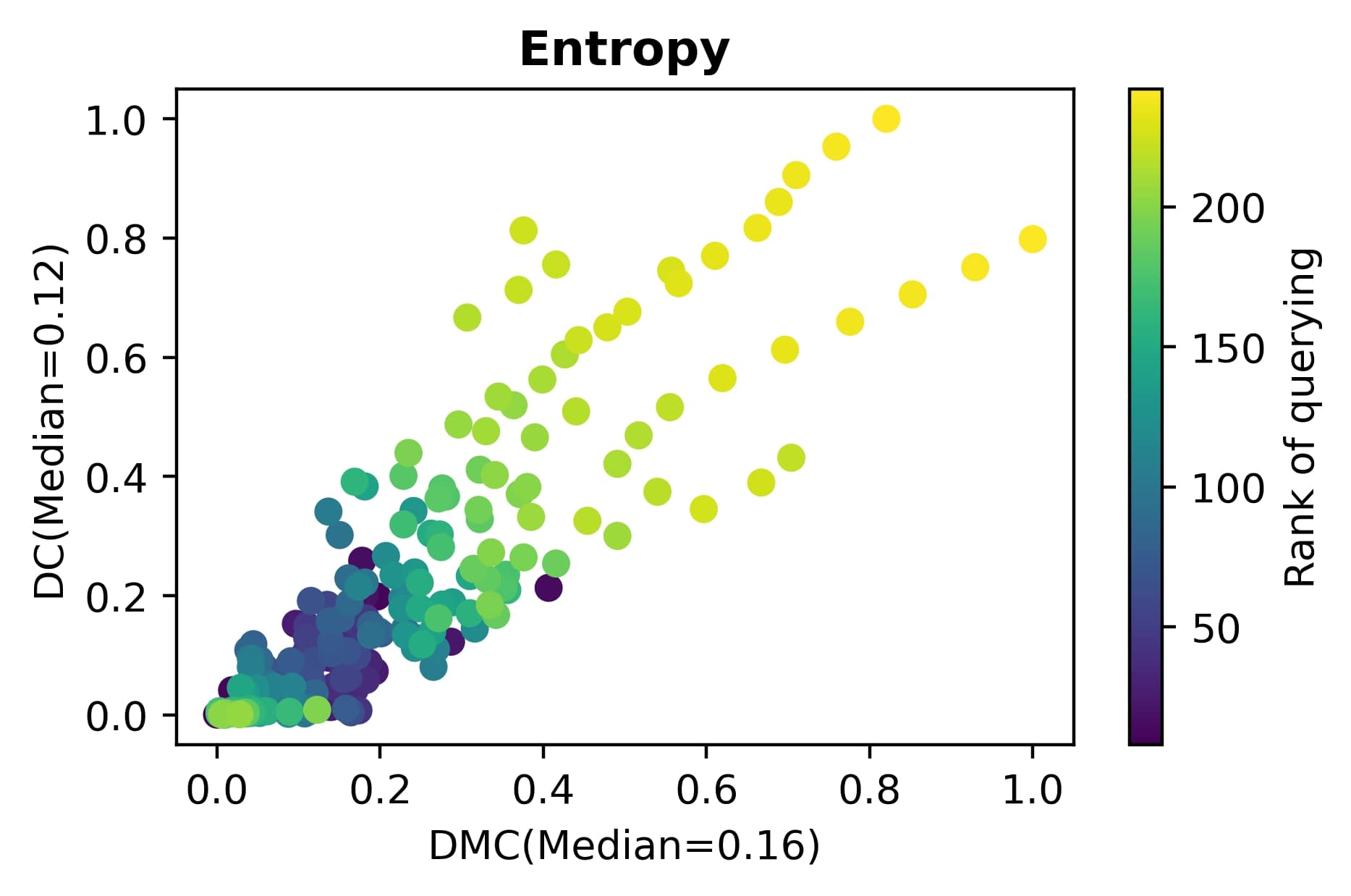

Supplement: Supplementary file 1 [file Data_Sheet_1.zip › Figures in Supplimentary Material/Entropy_Figure_8.jpg]

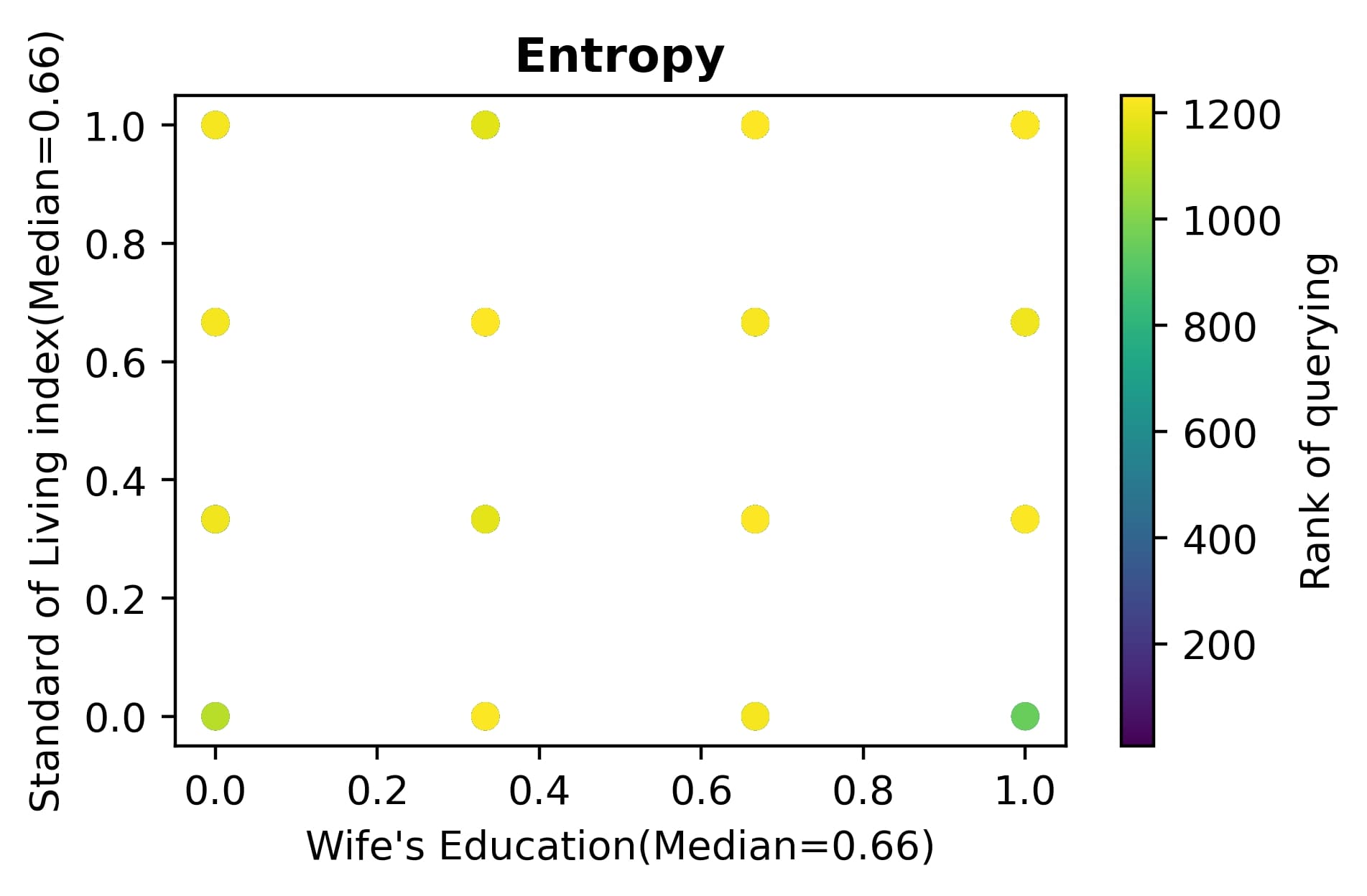

Supplement: Supplementary file 1 [file Data_Sheet_1.zip › Figures in Supplimentary Material/Entropy_Figure_9.jpg]

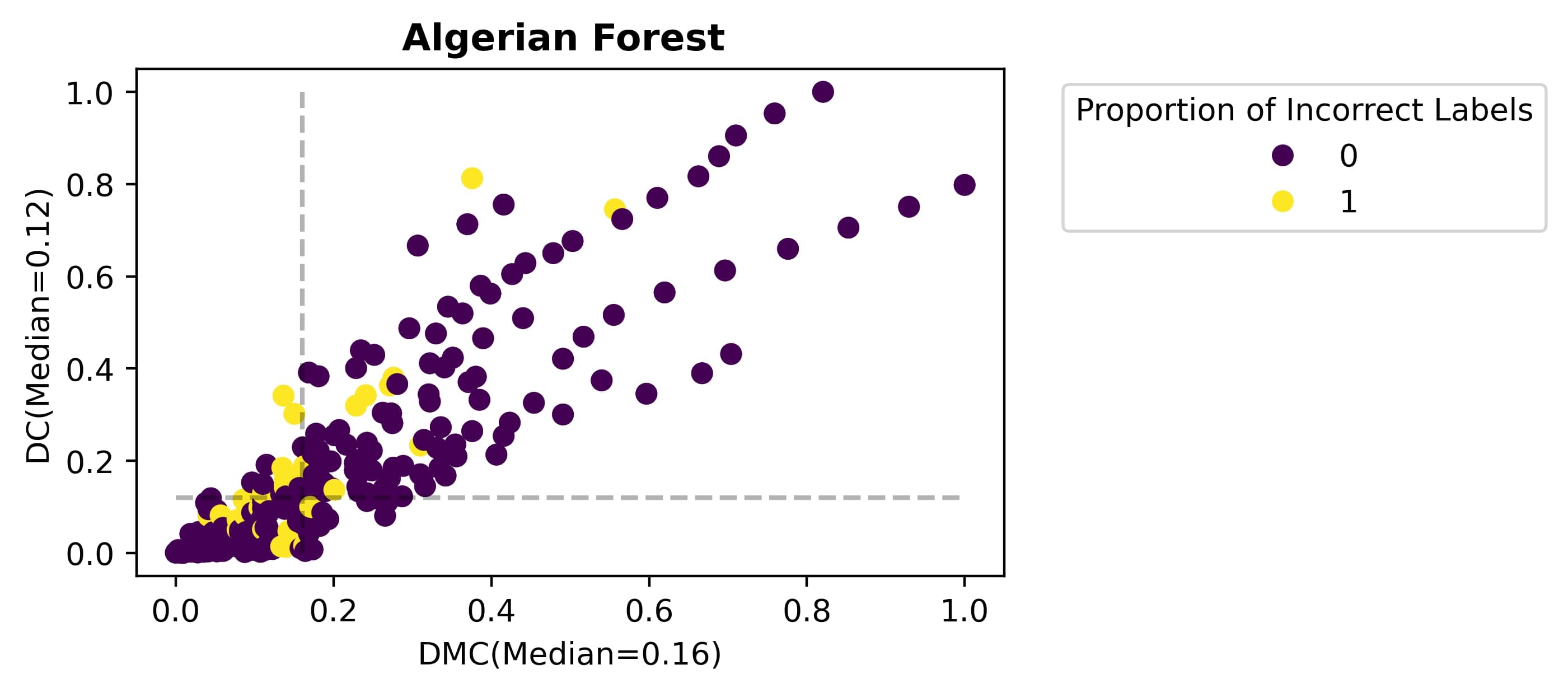

Supplement: Supplementary file 1 [file Data_Sheet_1.zip › Figures in Supplimentary Material/Figure 1/Algerian_Forest_Figure_1.jpg]

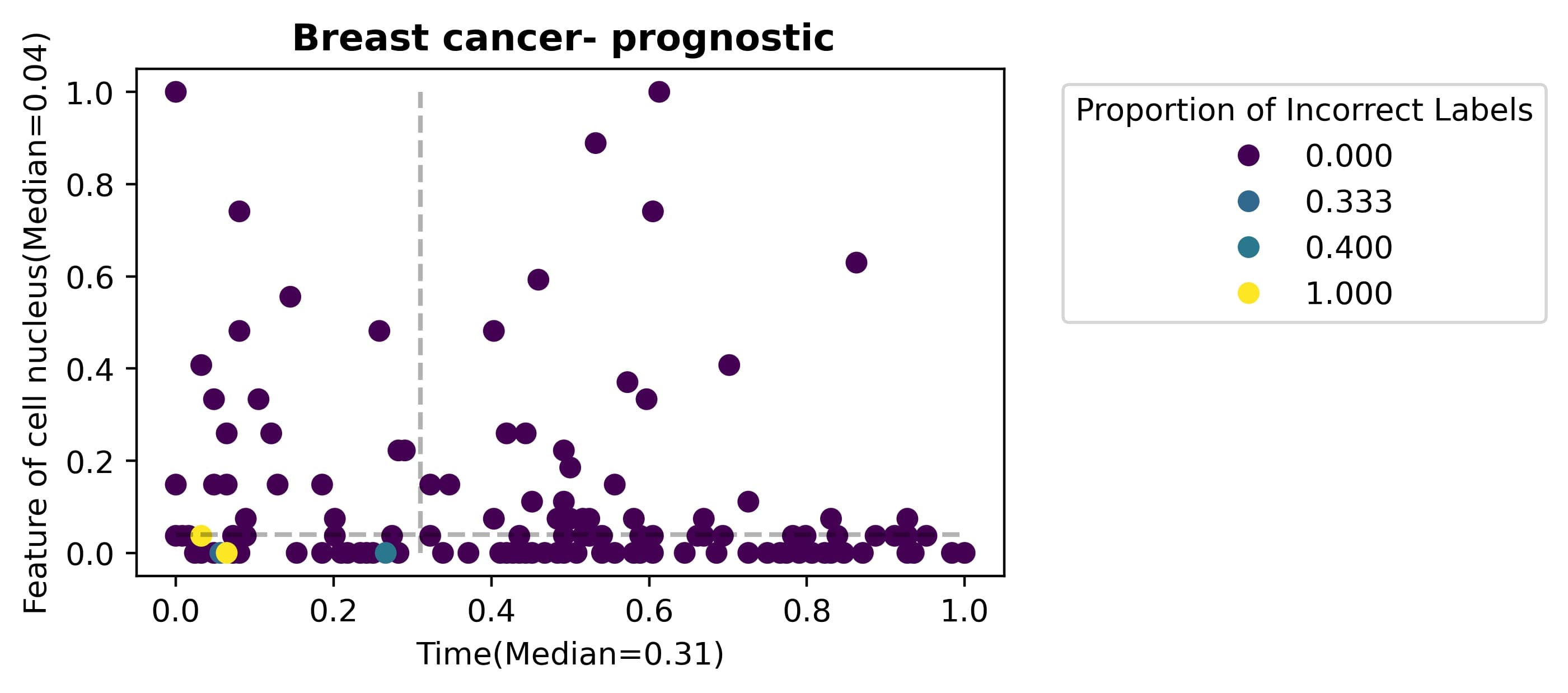

Supplement: Supplementary file 1 [file Data_Sheet_1.zip › Figures in Supplimentary Material/Figure 1/Breast_cancer_Figure_1.jpg]

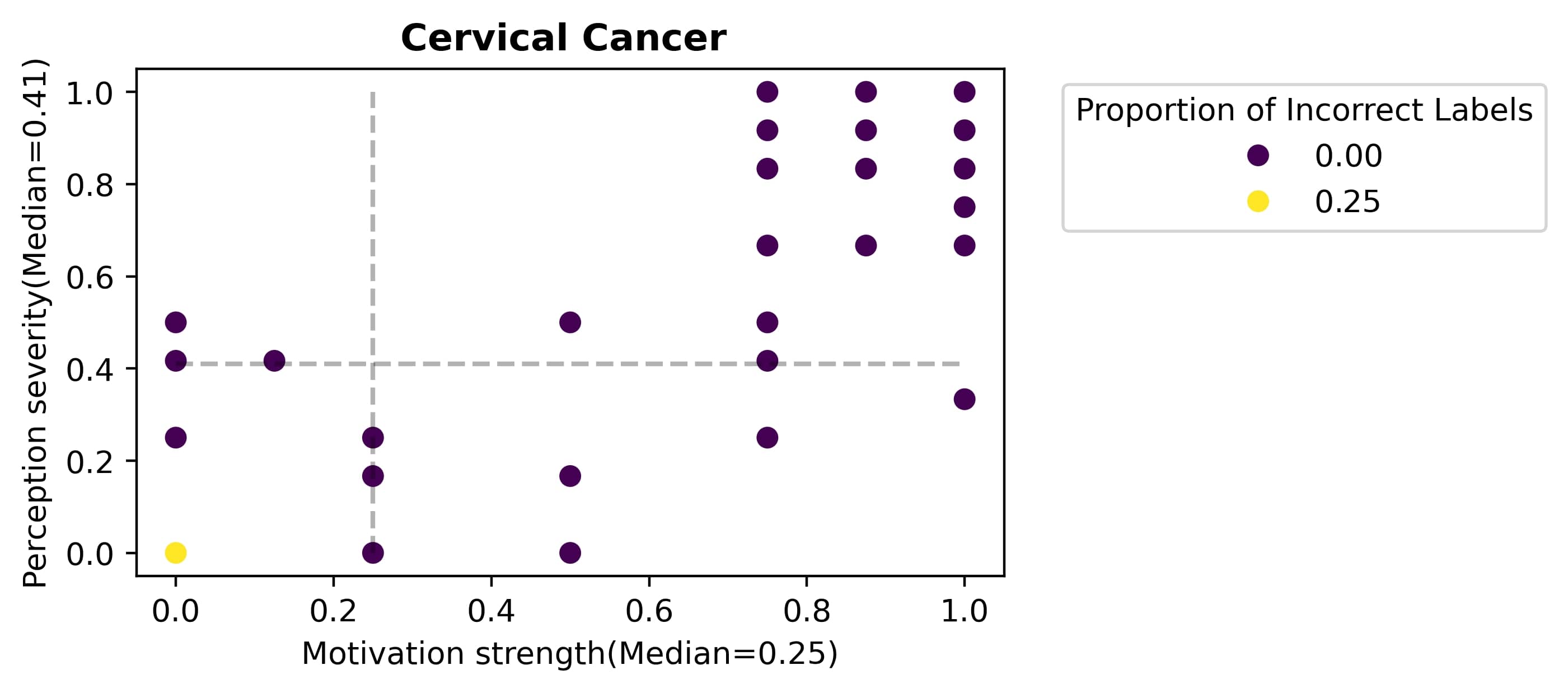

Supplement: Supplementary file 1 [file Data_Sheet_1.zip › Figures in Supplimentary Material/Figure 1/Cervical_cancer_Figure_1.jpg]

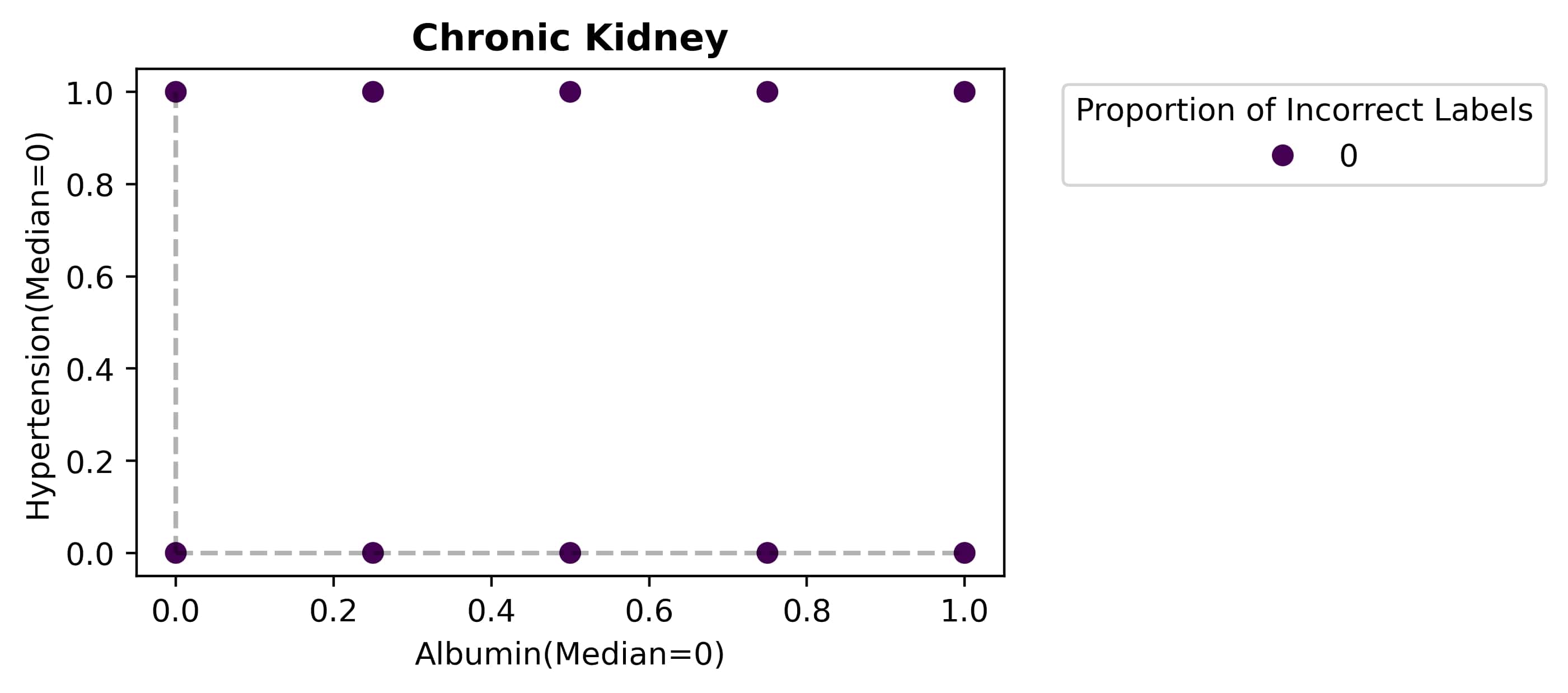

Supplement: Supplementary file 1 [file Data_Sheet_1.zip › Figures in Supplimentary Material/Figure 1/Chronic_Kidney_Figure_1.jpg]

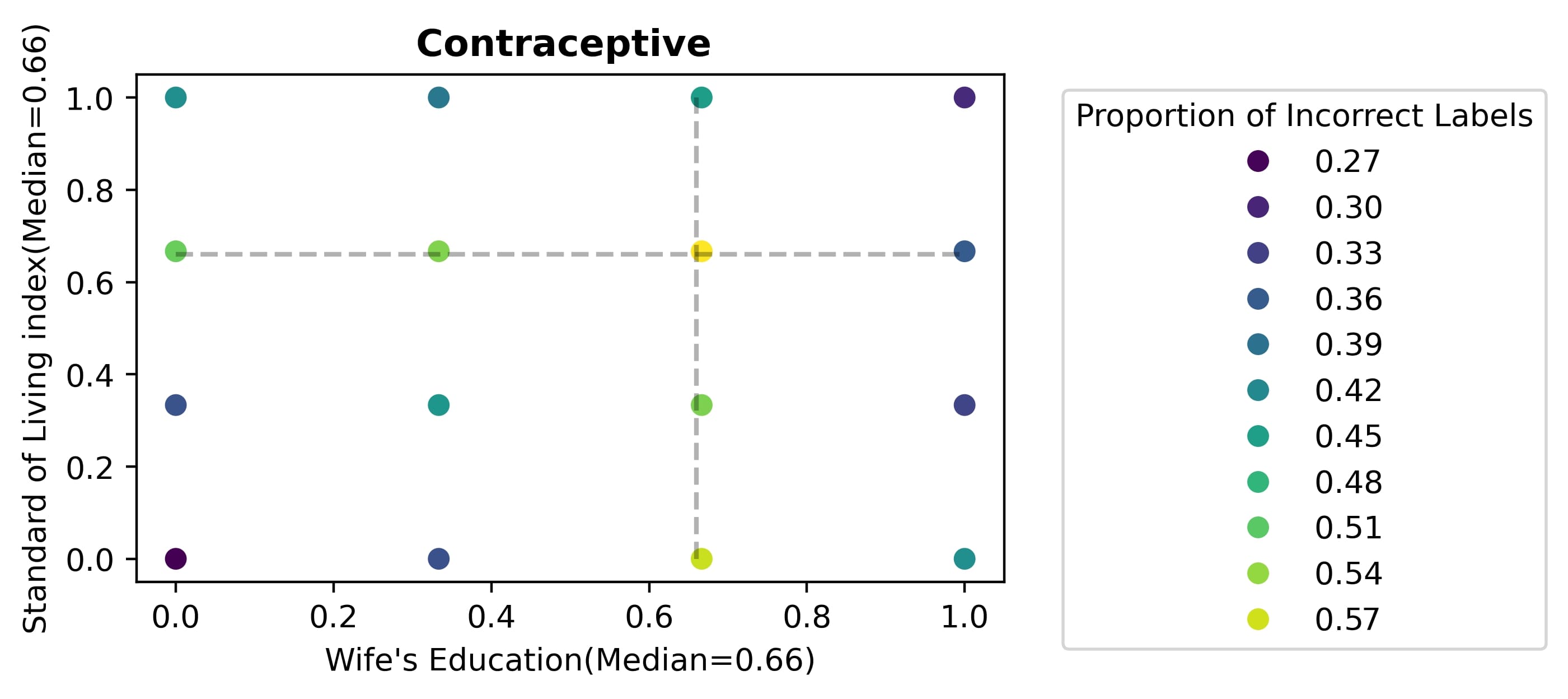

Supplement: Supplementary file 1 [file Data_Sheet_1.zip › Figures in Supplimentary Material/Figure 1/Contraceptive_Figure_1.jpg]

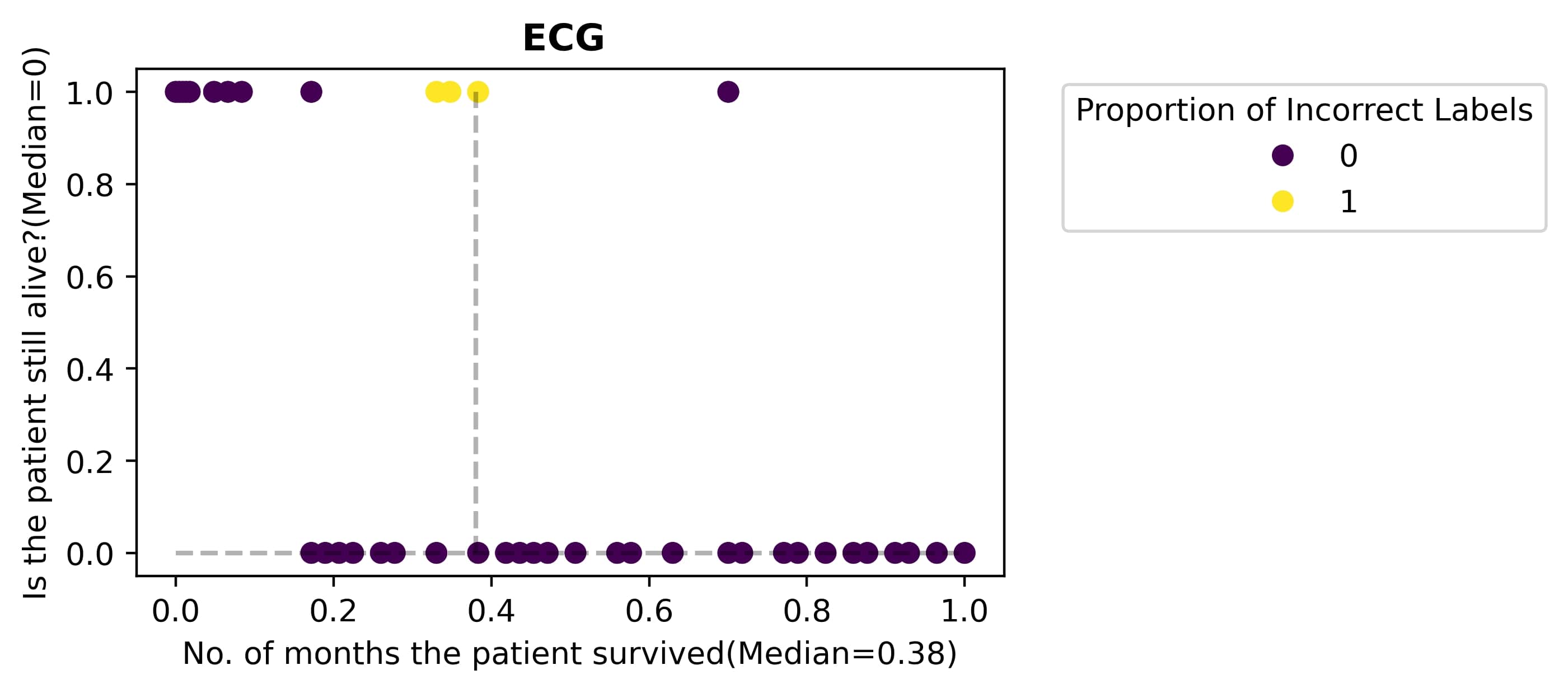

Supplement: Supplementary file 1 [file Data_Sheet_1.zip › Figures in Supplimentary Material/Figure 1/ECG_Figure_1.jpg]

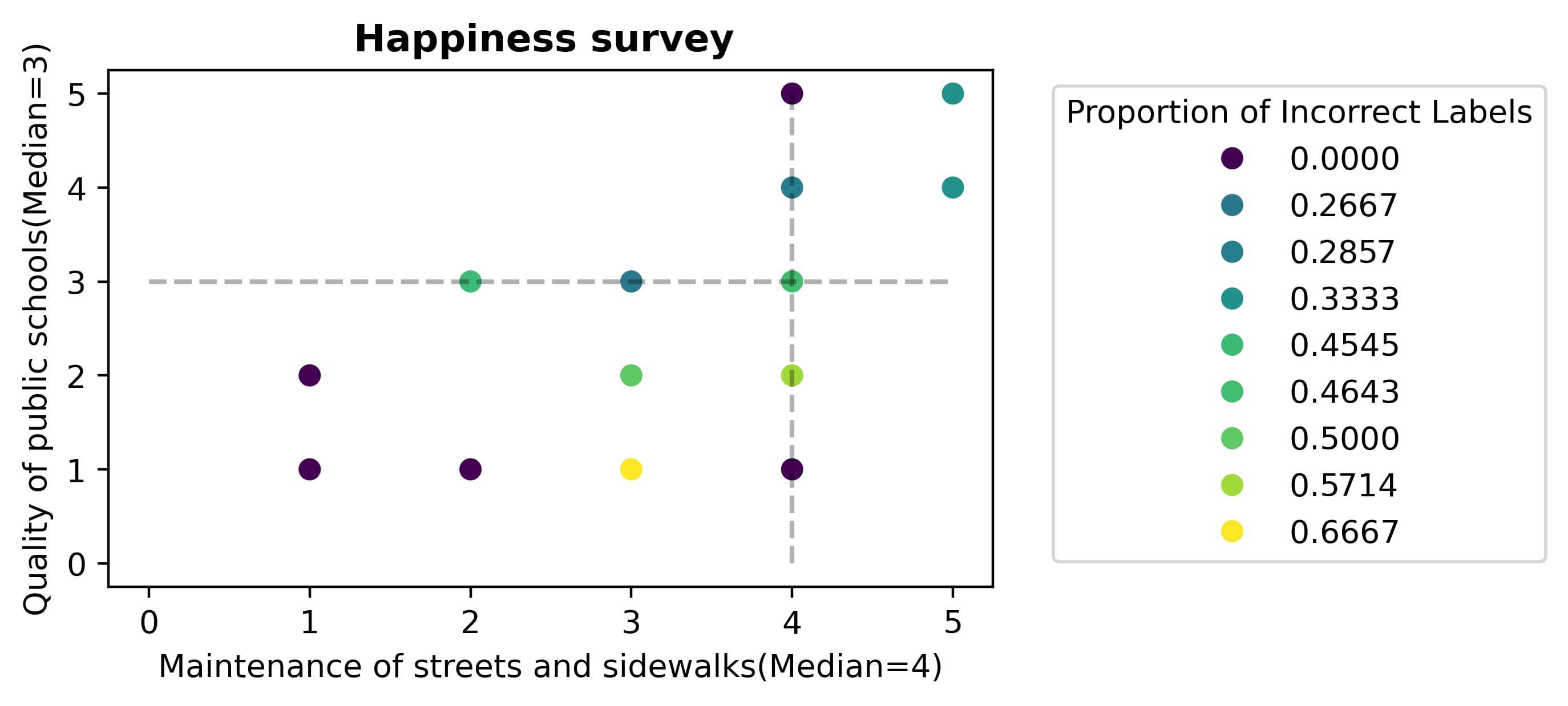

Supplement: Supplementary file 1 [file Data_Sheet_1.zip › Figures in Supplimentary Material/Figure 1/Happiness_survey_Figure_1.jpg]

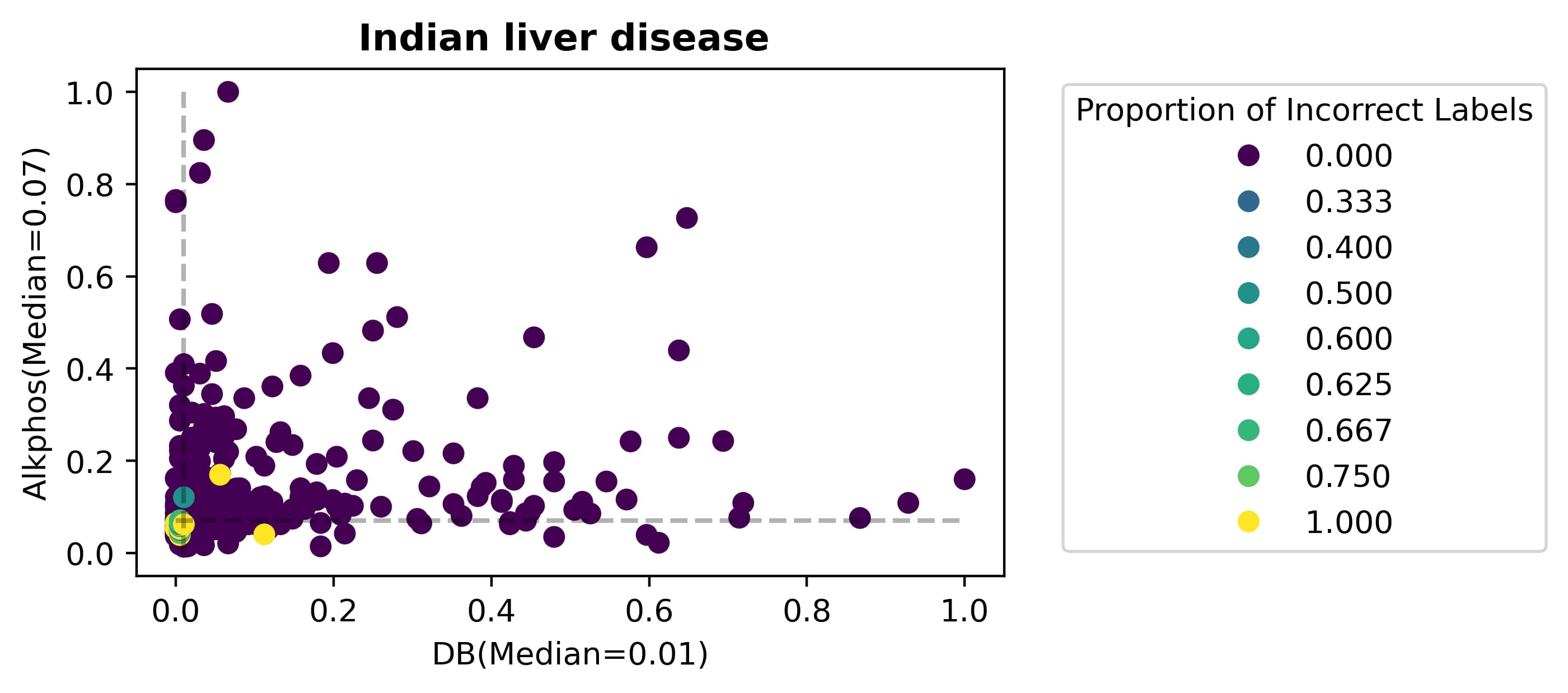

Supplement: Supplementary file 1 [file Data_Sheet_1.zip › Figures in Supplimentary Material/Figure 1/Indian_liver_Figure_1.jpg]

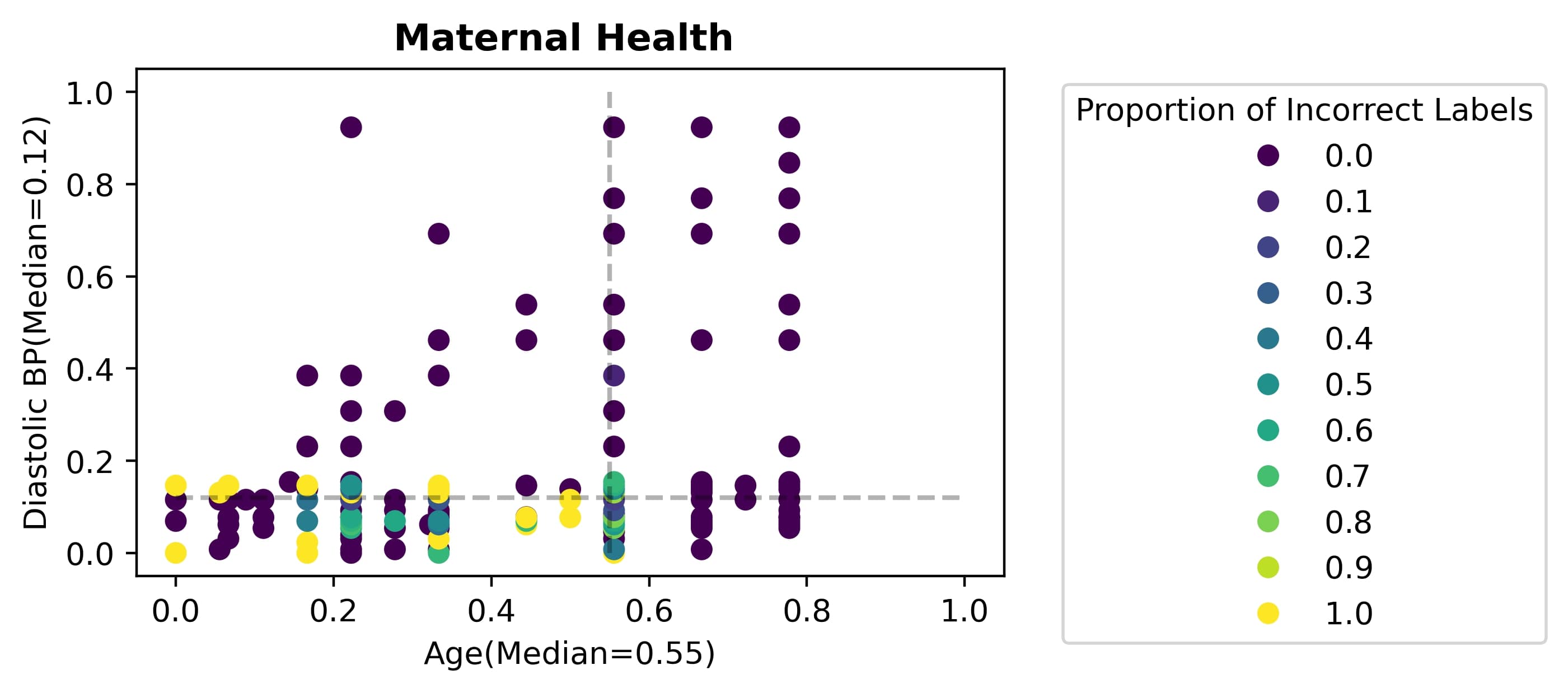

Supplement: Supplementary file 1 [file Data_Sheet_1.zip › Figures in Supplimentary Material/Figure 1/Maternal_Health_Figure_1.jpg]

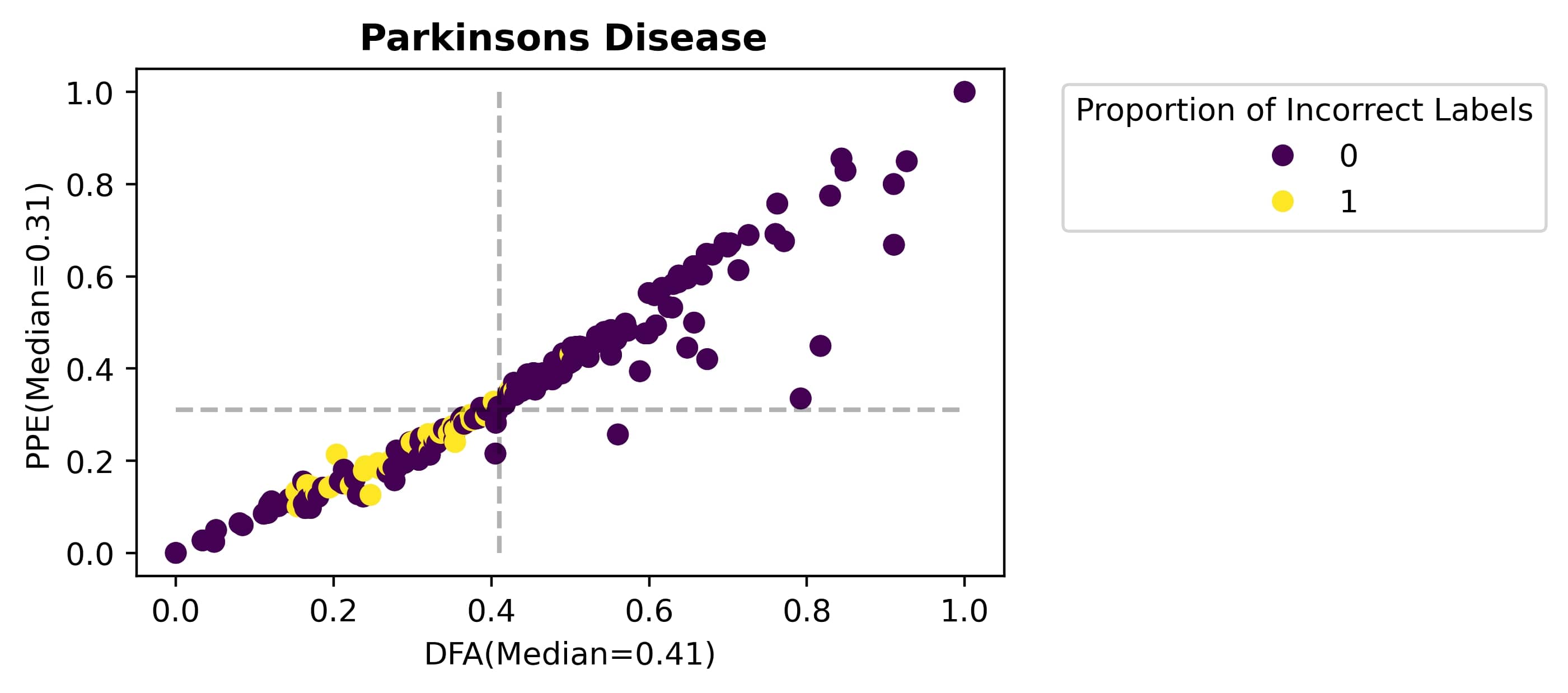

Supplement: Supplementary file 1 [file Data_Sheet_1.zip › Figures in Supplimentary Material/Figure 1/Parkinsons_Figure_1.jpg]

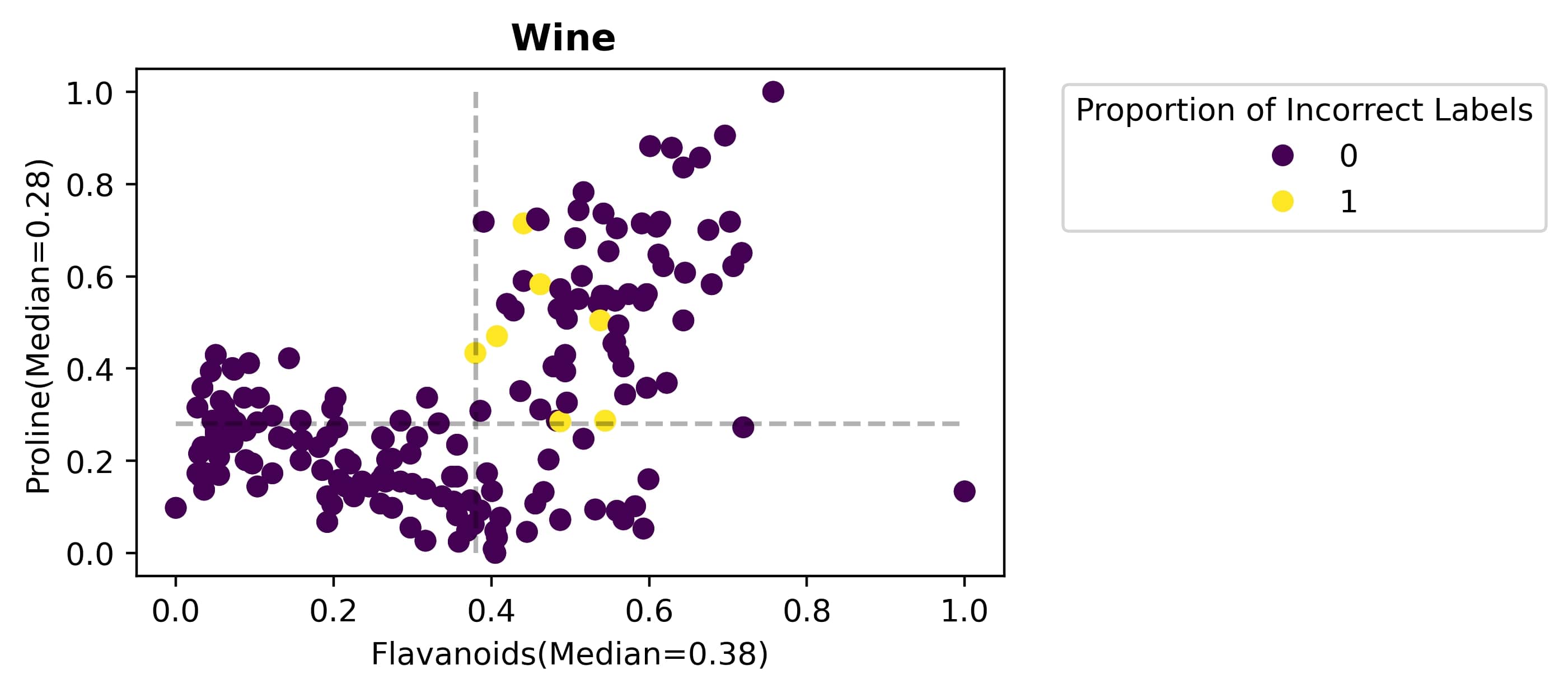

Supplement: Supplementary file 1 [file Data_Sheet_1.zip › Figures in Supplimentary Material/Figure 1/Wine_Figure_1.jpg]

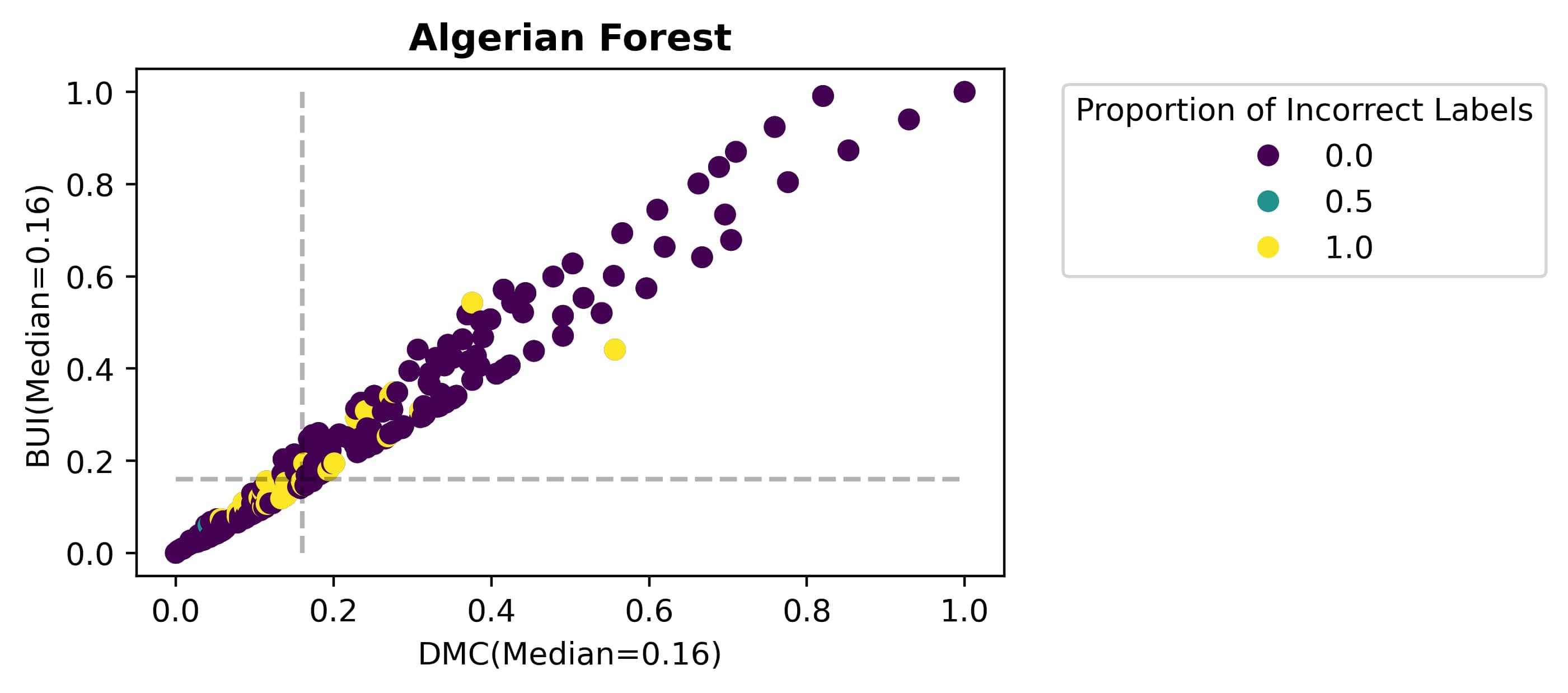

Supplement: Supplementary file 1 [file Data_Sheet_1.zip › Figures in Supplimentary Material/Figure 2/Algerian_Forest_Figure_2.jpg]

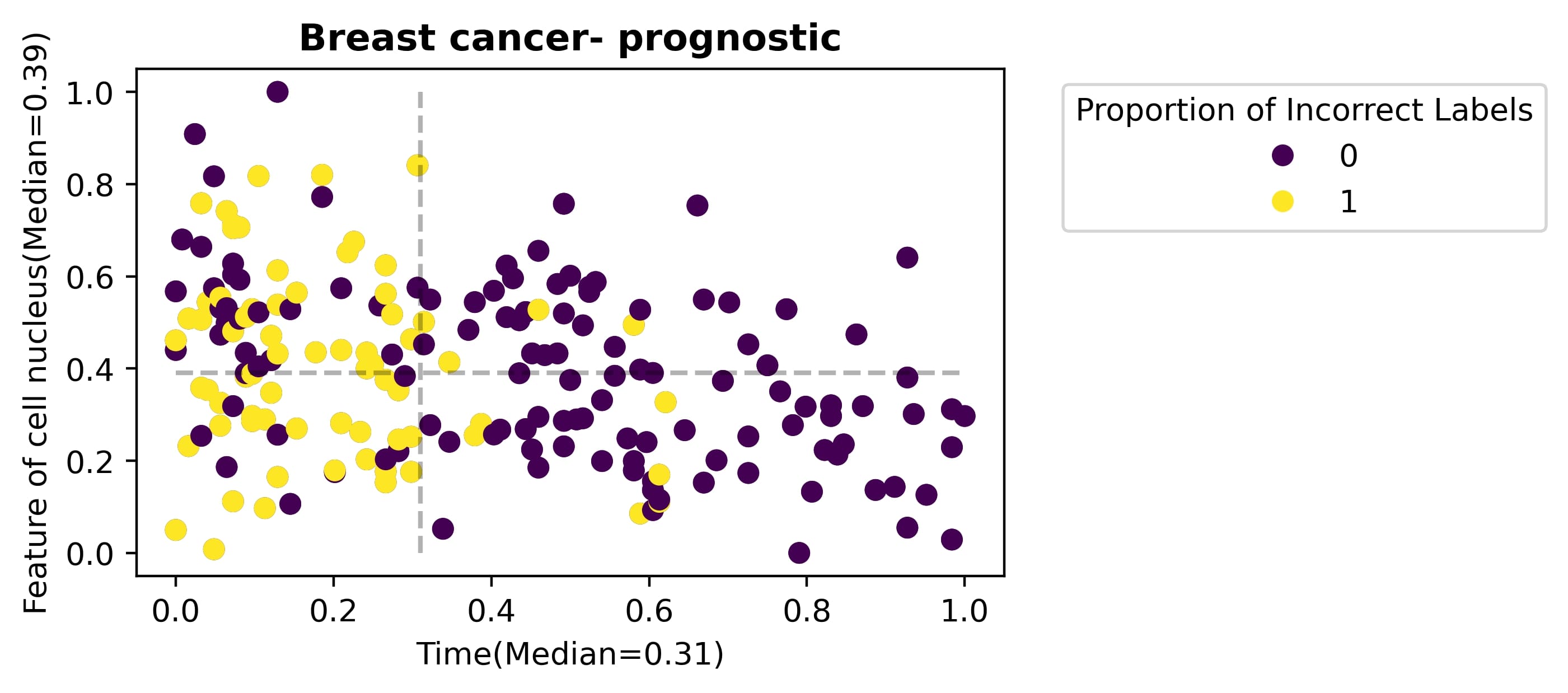

Supplement: Supplementary file 1 [file Data_Sheet_1.zip › Figures in Supplimentary Material/Figure 2/Breast_Cancer_Figure_2.jpg]

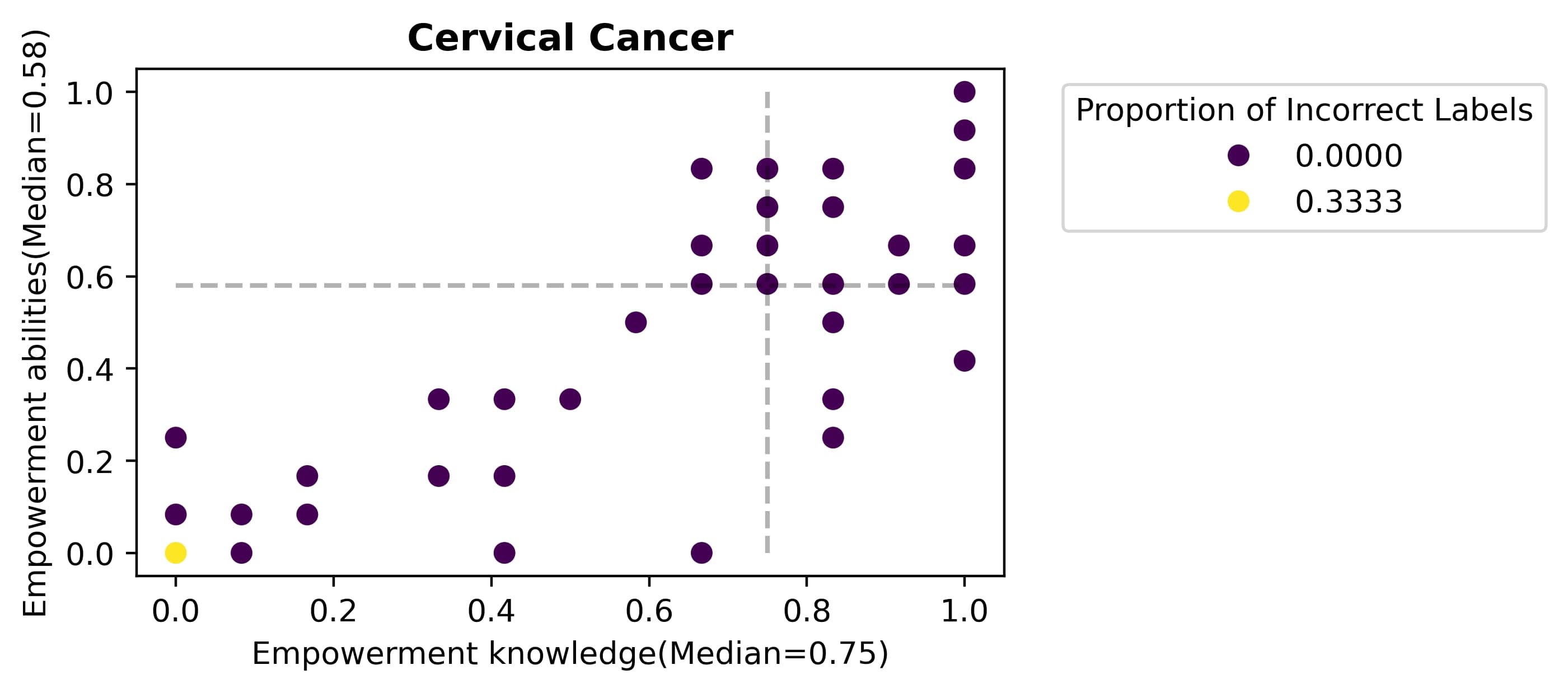

Supplement: Supplementary file 1 [file Data_Sheet_1.zip › Figures in Supplimentary Material/Figure 2/Cervical_Cancer_Figure_2.jpg]

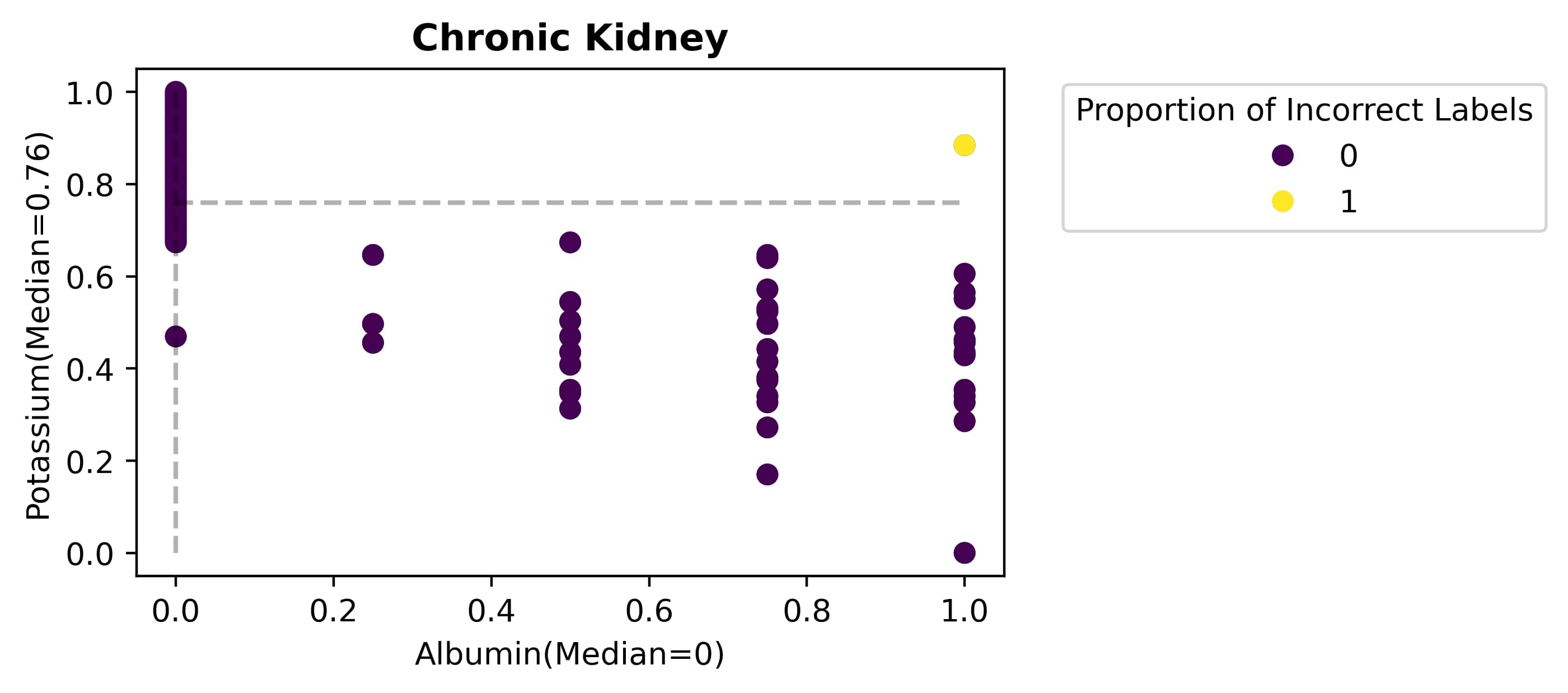

Supplement: Supplementary file 1 [file Data_Sheet_1.zip › Figures in Supplimentary Material/Figure 2/Chronic Kidney_Figure_2.jpg]

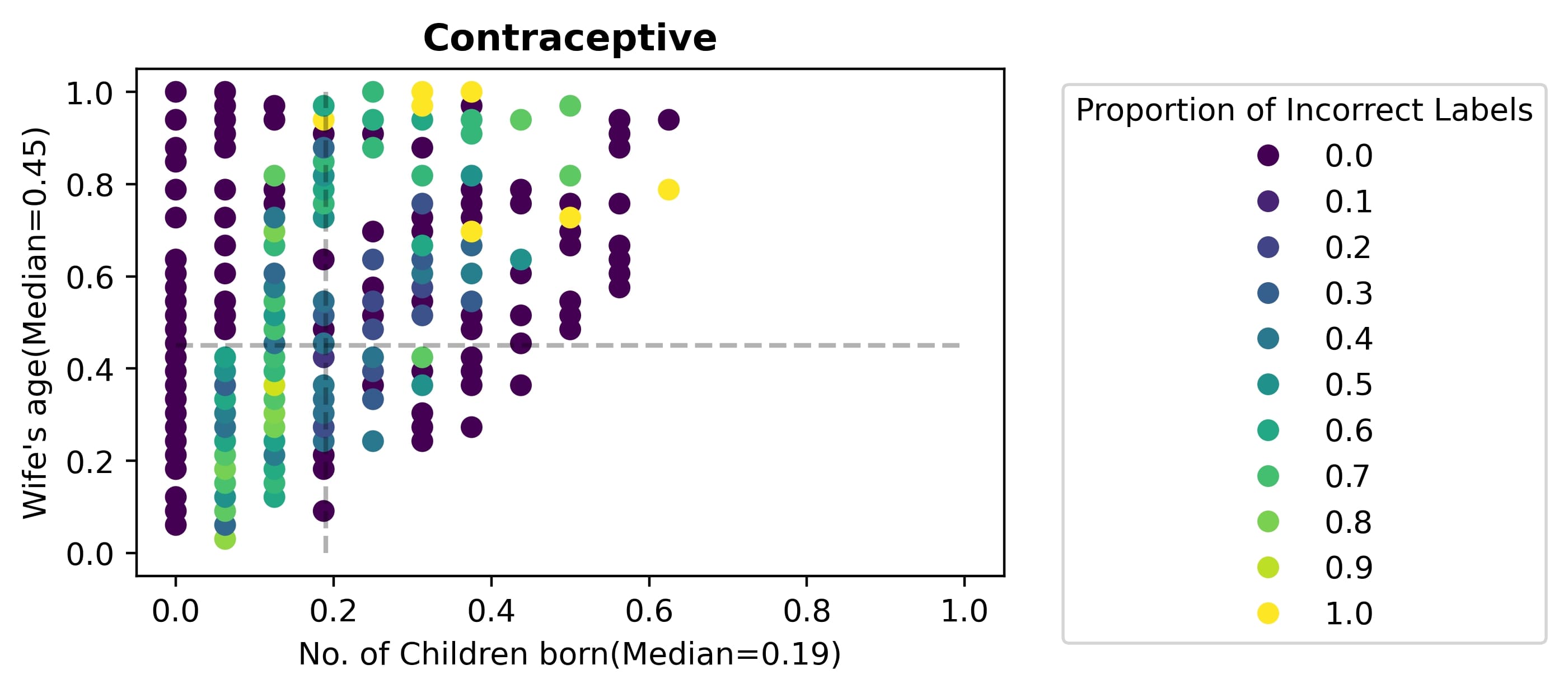

Supplement: Supplementary file 1 [file Data_Sheet_1.zip › Figures in Supplimentary Material/Figure 2/Contraceptive_Figure_2.jpg]

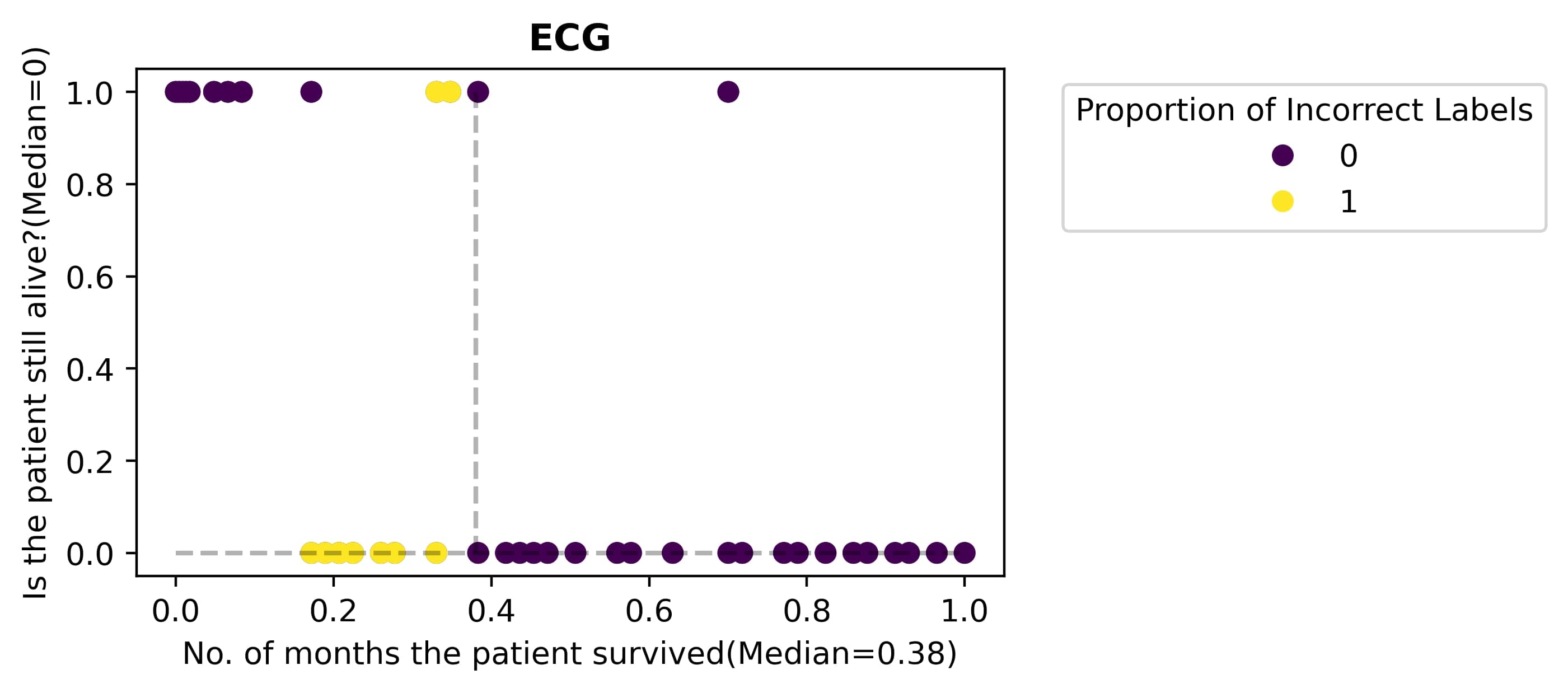

Supplement: Supplementary file 1 [file Data_Sheet_1.zip › Figures in Supplimentary Material/Figure 2/ECG_Figure_2.jpg]

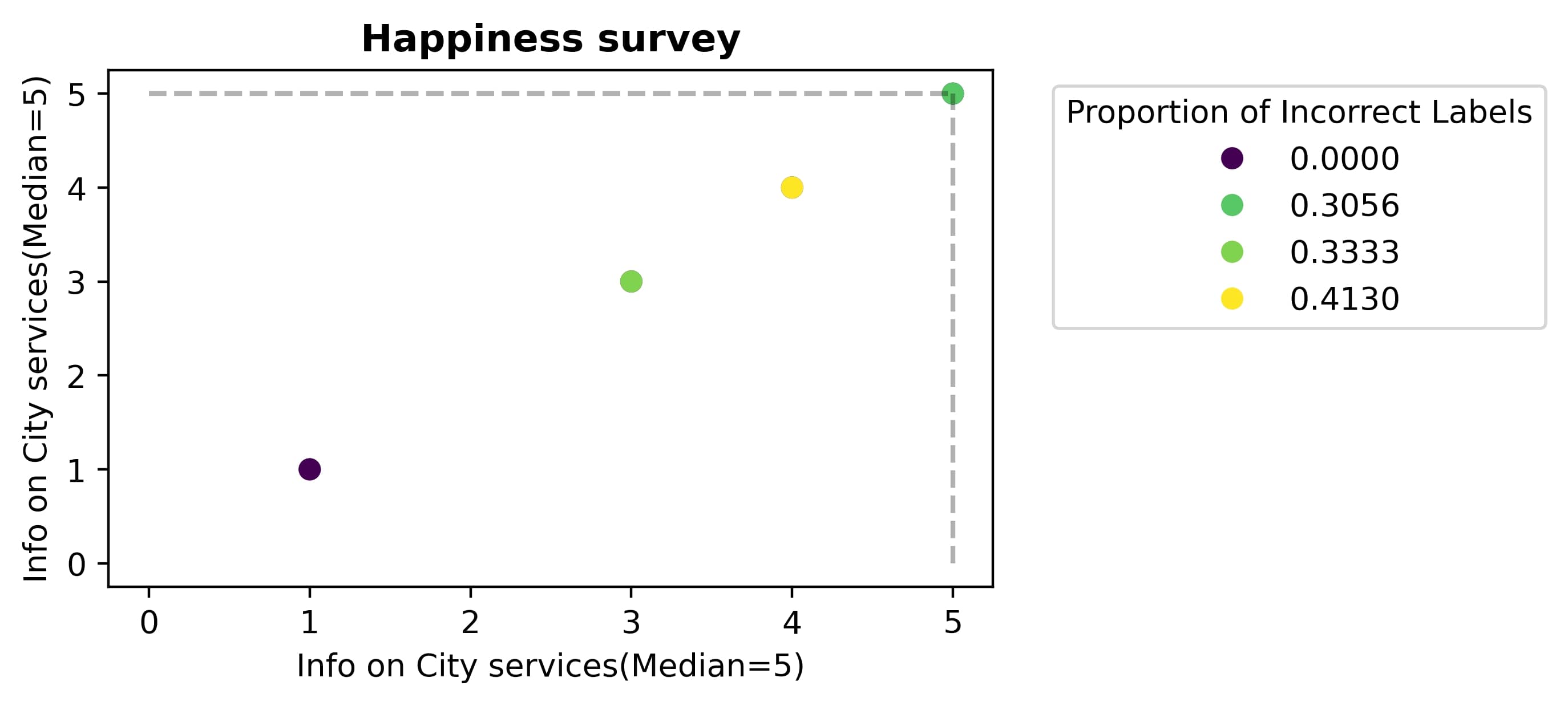

Supplement: Supplementary file 1 [file Data_Sheet_1.zip › Figures in Supplimentary Material/Figure 2/Happiness_Survey_Figure_2.jpg]

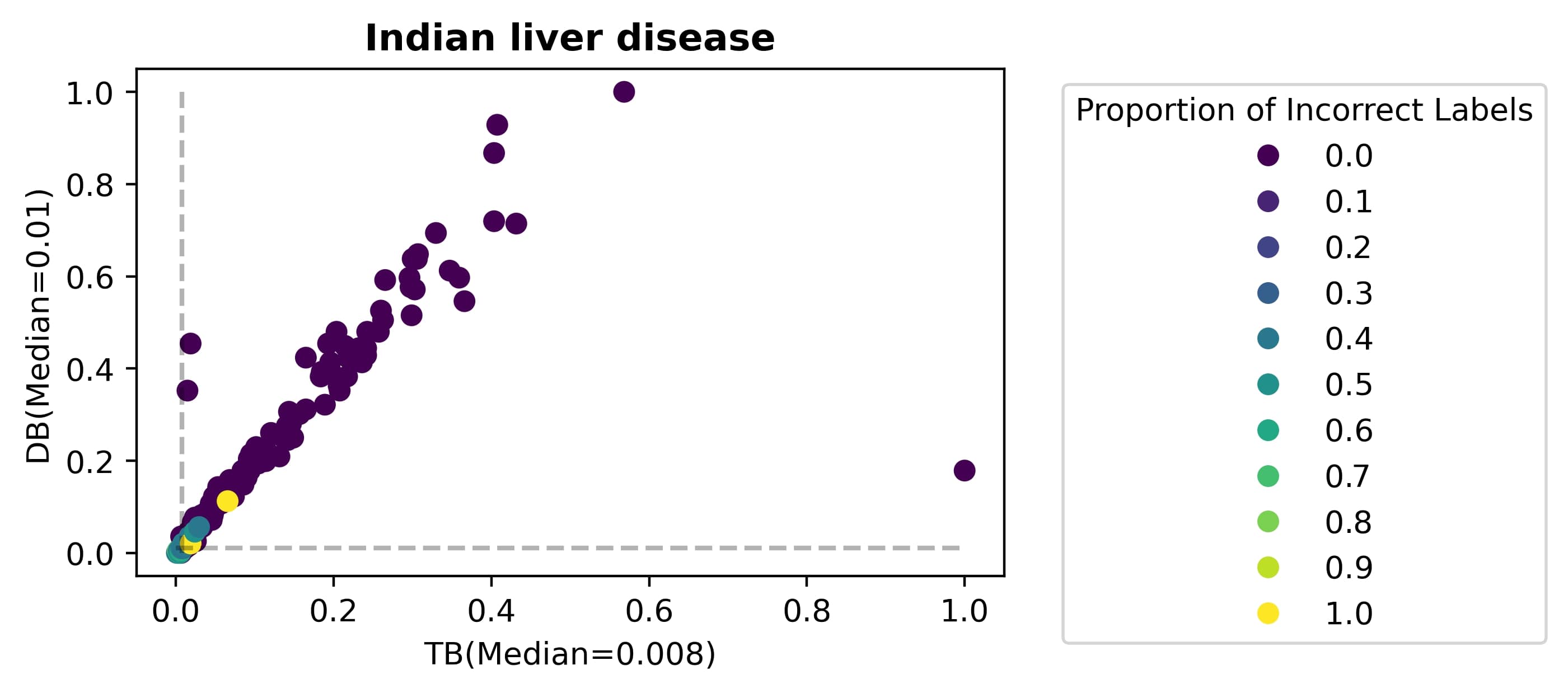

Supplement: Supplementary file 1 [file Data_Sheet_1.zip › Figures in Supplimentary Material/Figure 2/Indian_liver_Figure_2.jpg]

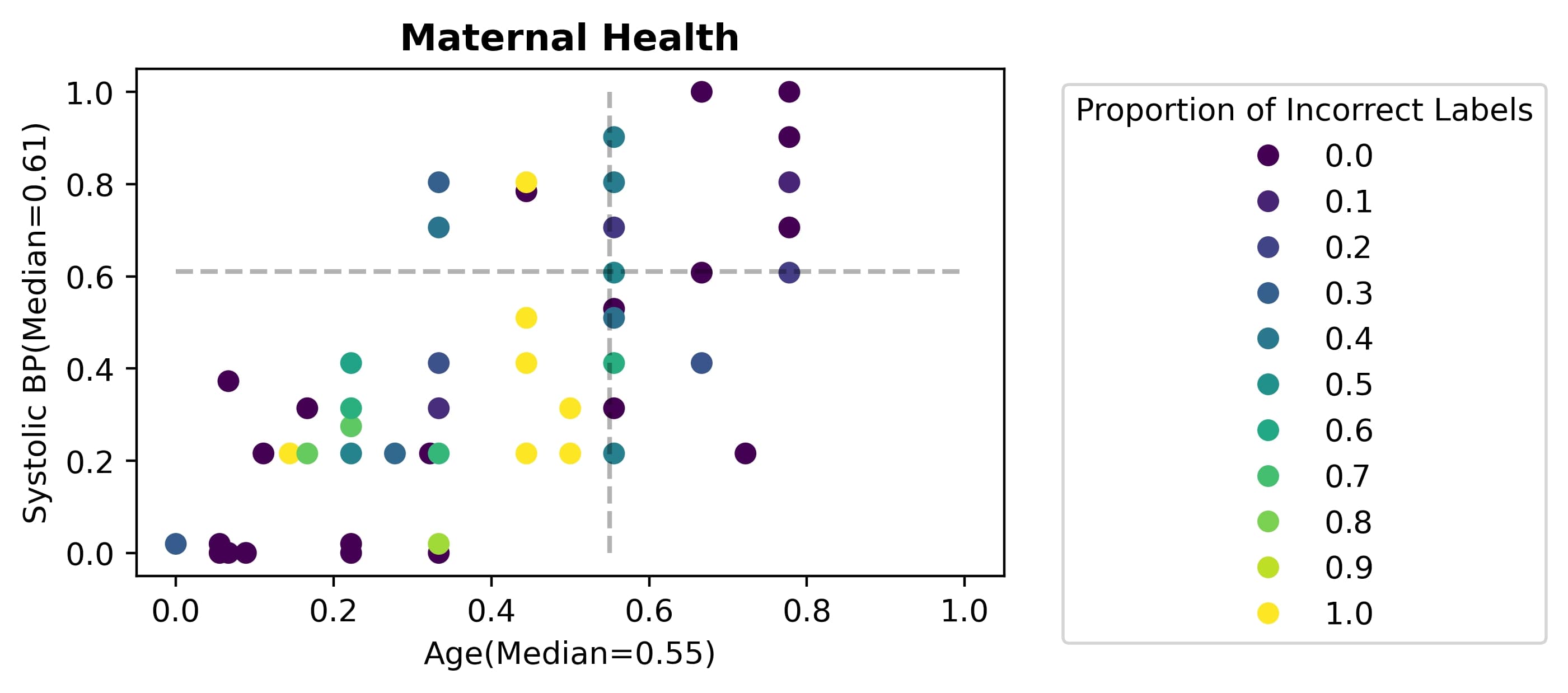

Supplement: Supplementary file 1 [file Data_Sheet_1.zip › Figures in Supplimentary Material/Figure 2/Maternal_health_Figure_2.jpg]

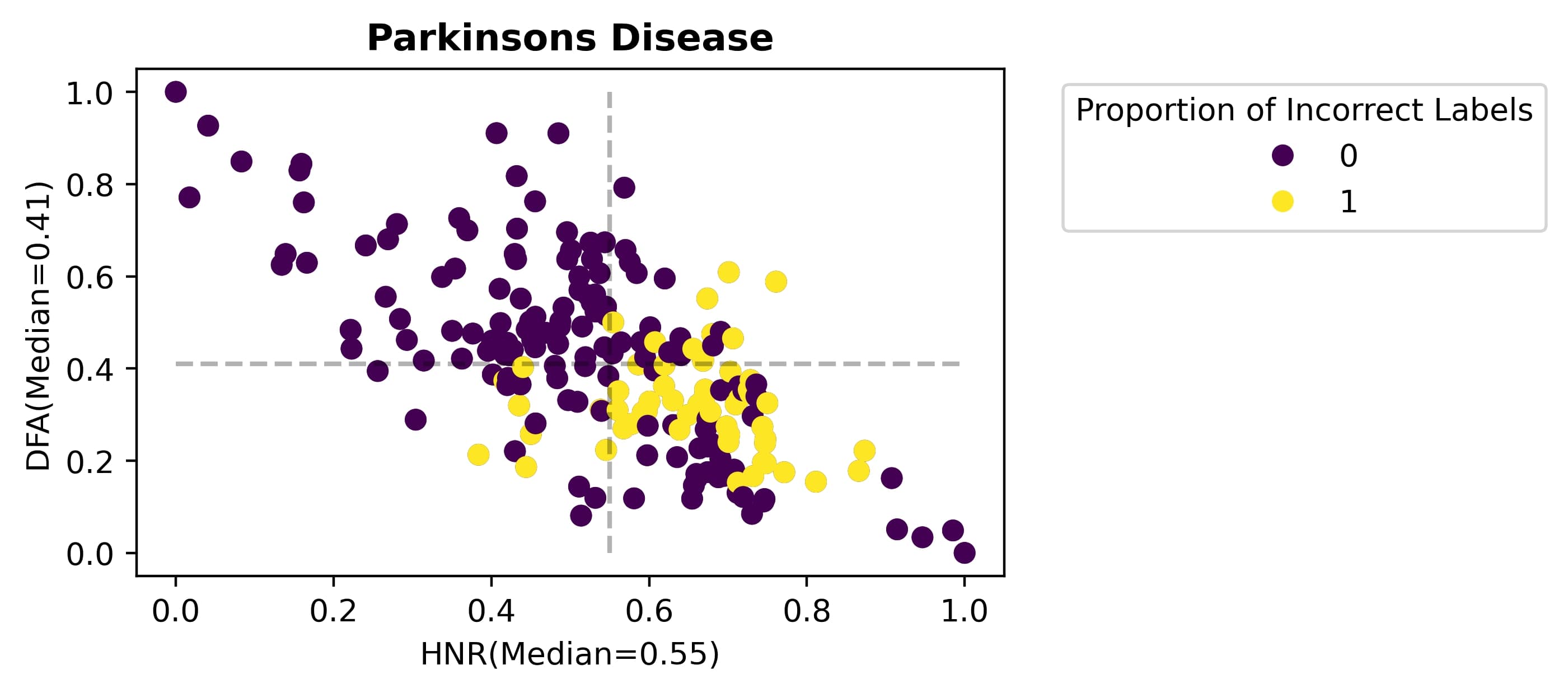

Supplement: Supplementary file 1 [file Data_Sheet_1.zip › Figures in Supplimentary Material/Figure 2/Parkinsons_Disease_Figure_2.jpg]

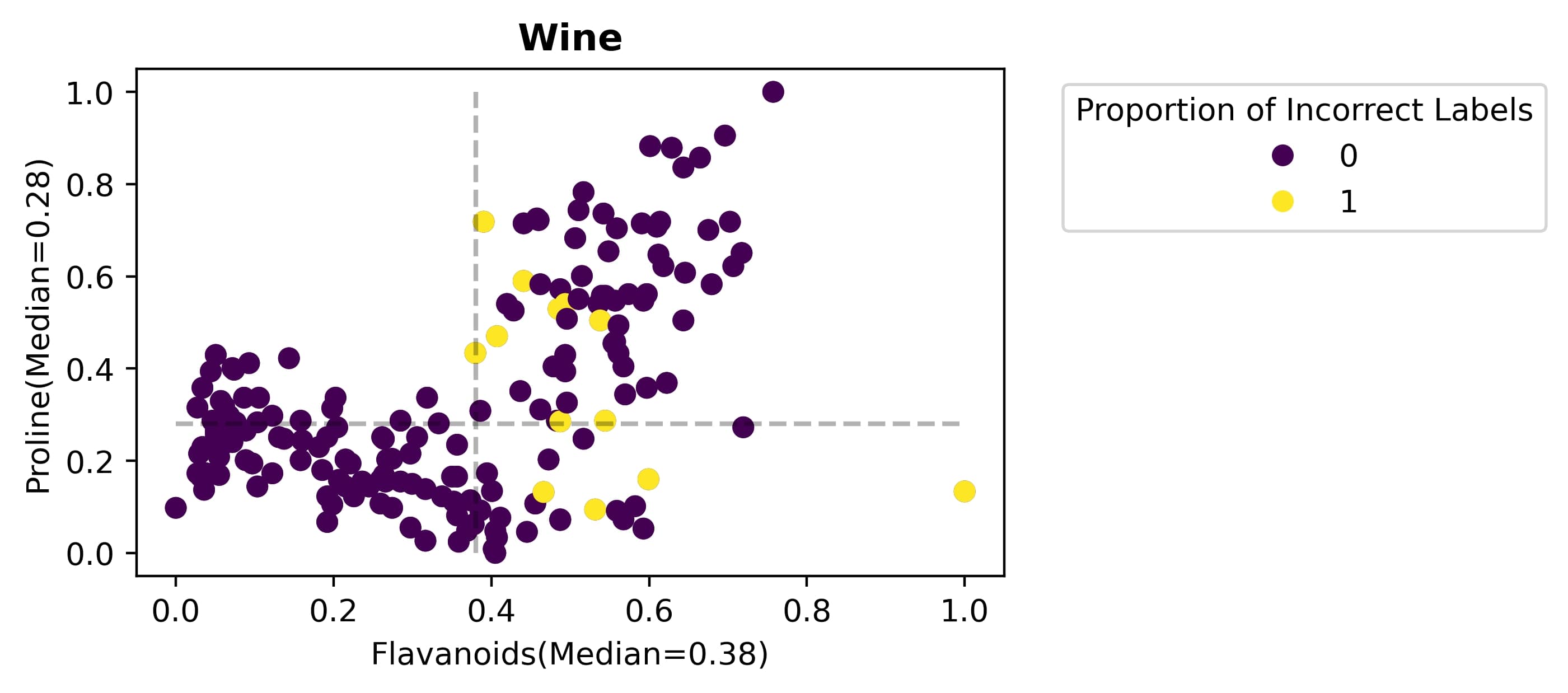

Supplement: Supplementary file 1 [file Data_Sheet_1.zip › Figures in Supplimentary Material/Figure 2/Wine_Figure_2.jpg]

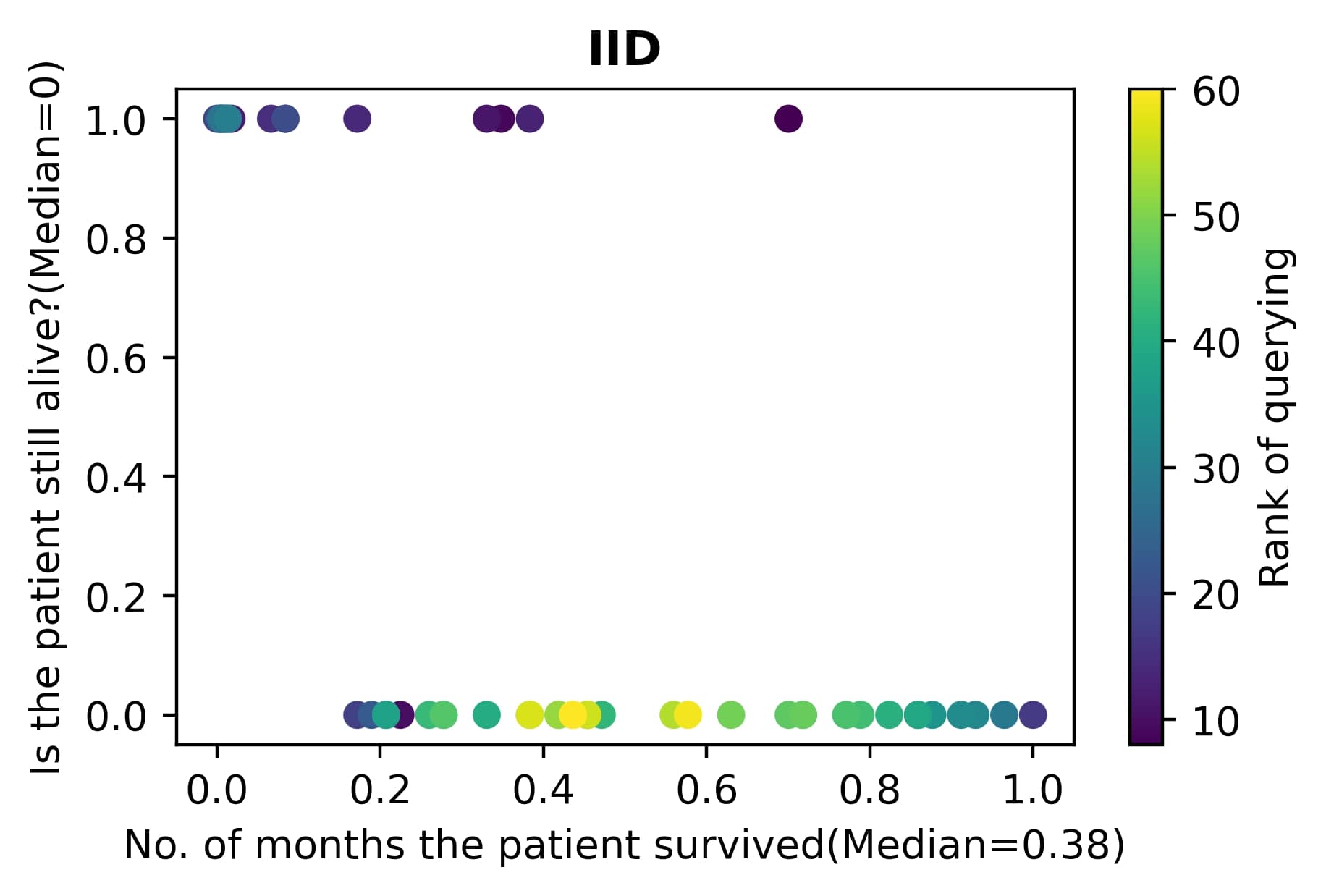

Supplement: Supplementary file 1 [file Data_Sheet_1.zip › Figures in Supplimentary Material/IID_Figure_10.jpg]

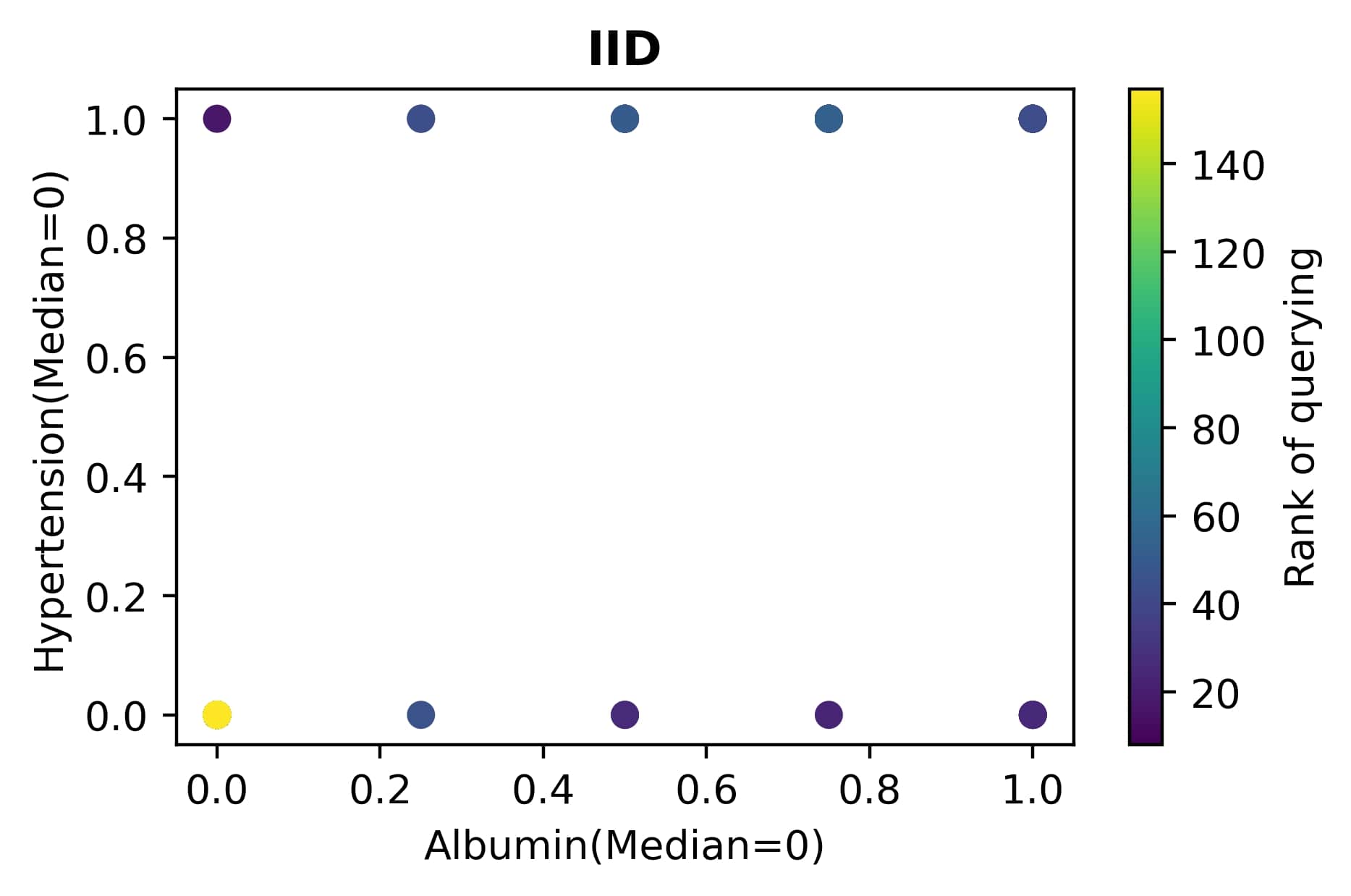

Supplement: Supplementary file 1 [file Data_Sheet_1.zip › Figures in Supplimentary Material/IID_Figure_11.jpg]

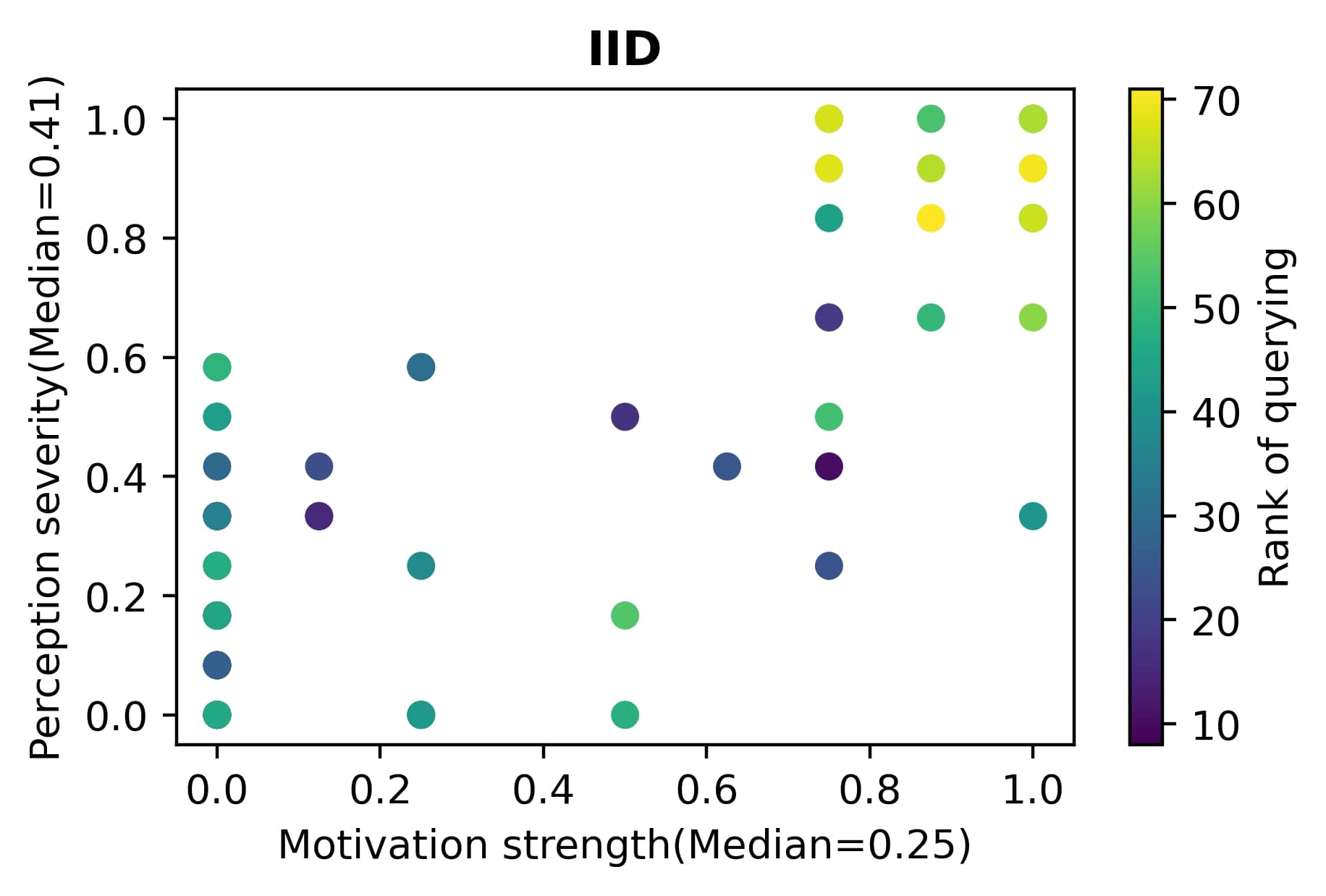

Supplement: Supplementary file 1 [file Data_Sheet_1.zip › Figures in Supplimentary Material/IID_Figure_12.jpg]

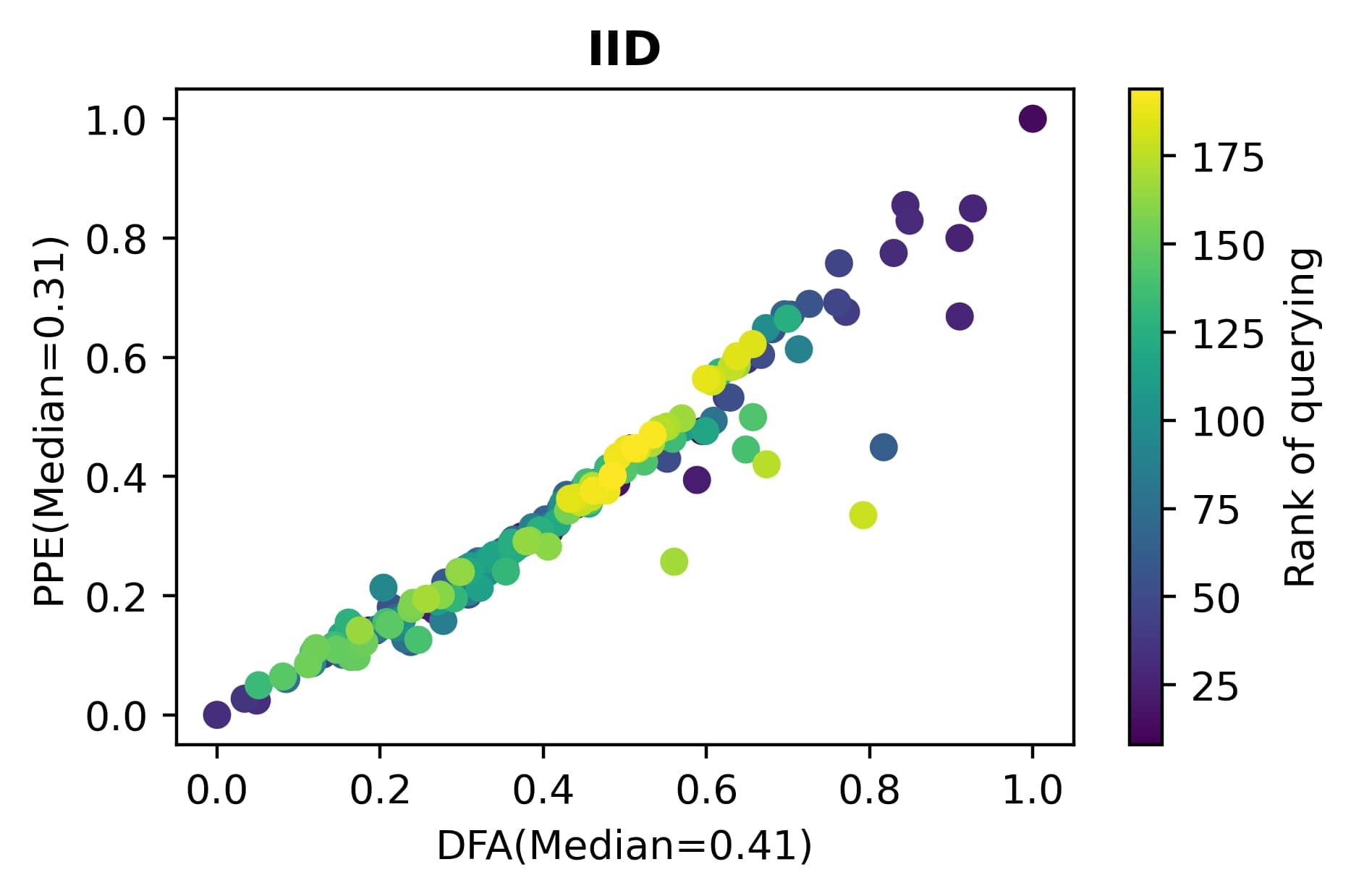

Supplement: Supplementary file 1 [file Data_Sheet_1.zip › Figures in Supplimentary Material/IID_Figure_13.jpg]

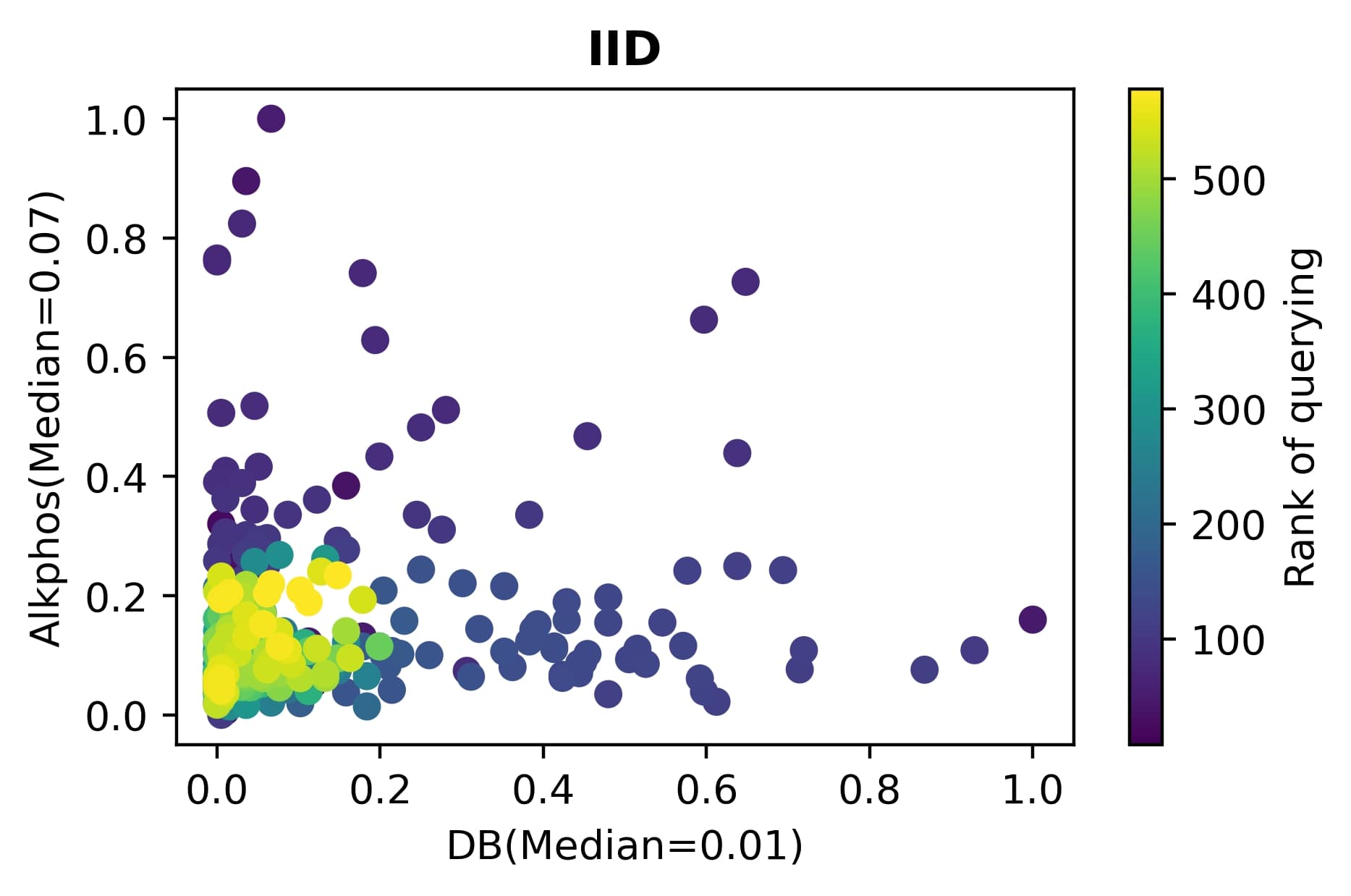

Supplement: Supplementary file 1 [file Data_Sheet_1.zip › Figures in Supplimentary Material/IID_Figure_14.jpg]

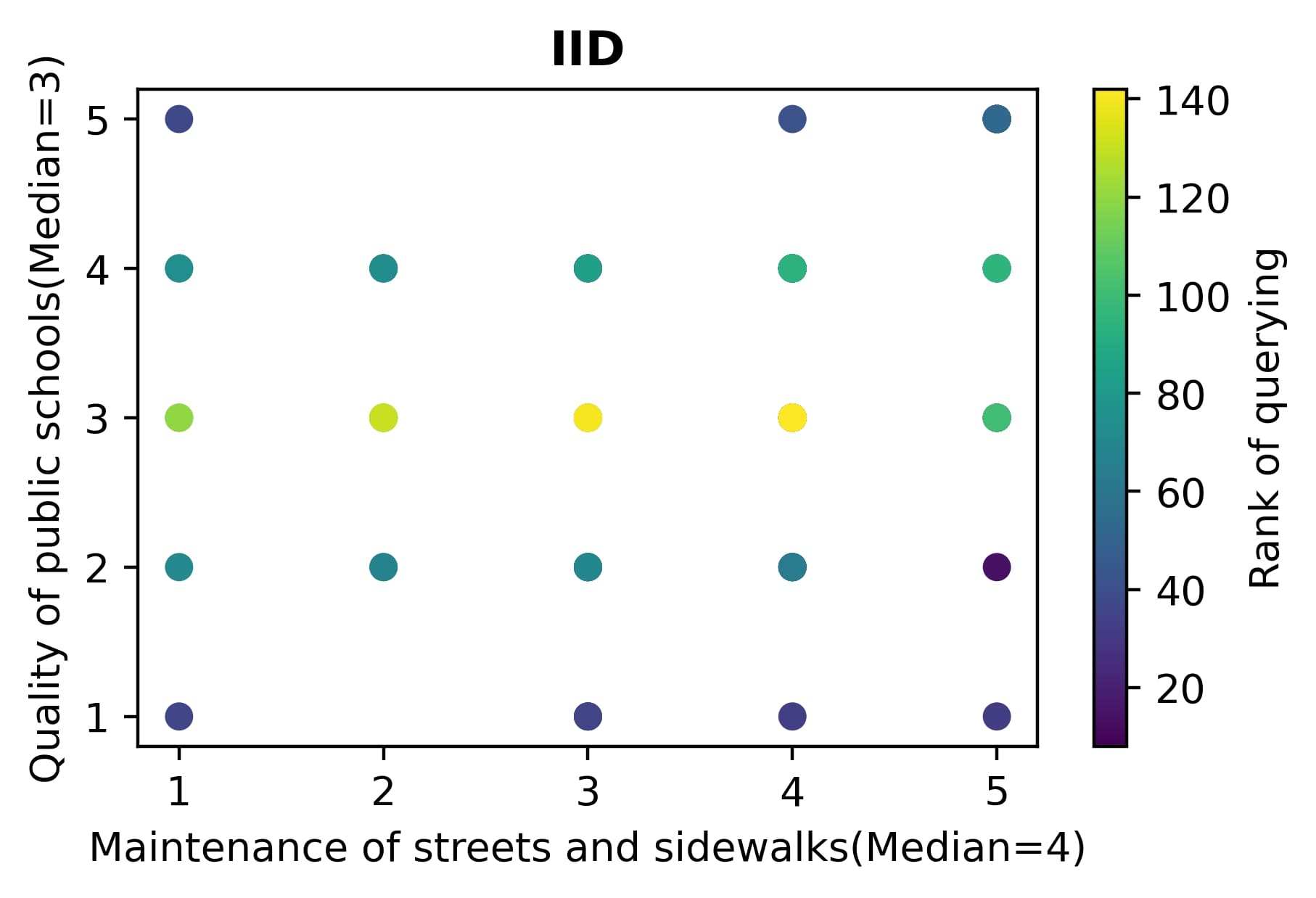

Supplement: Supplementary file 1 [file Data_Sheet_1.zip › Figures in Supplimentary Material/IID_Figure_15.jpg]

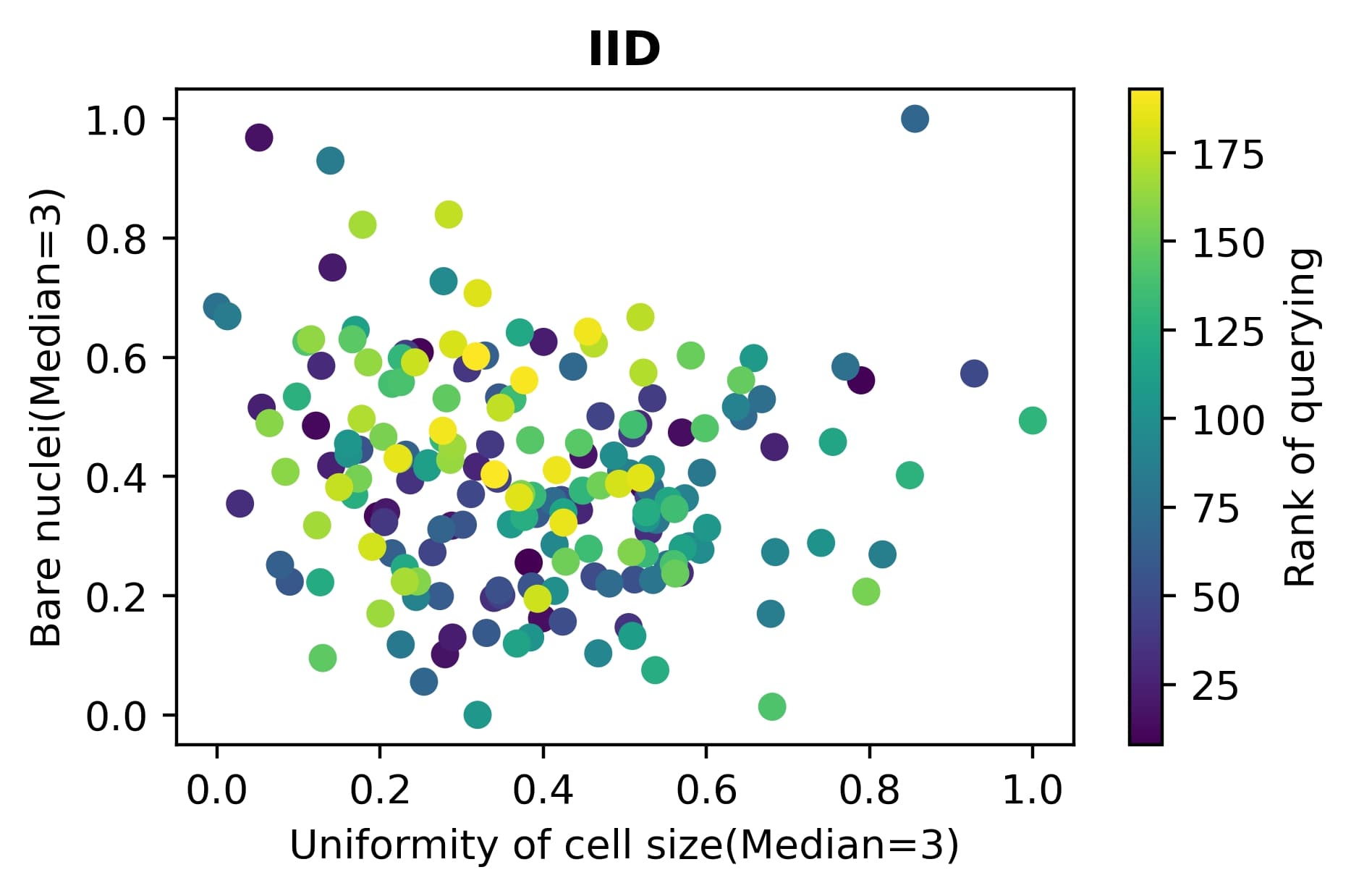

Supplement: Supplementary file 1 [file Data_Sheet_1.zip › Figures in Supplimentary Material/IID_Figure_16.jpg]

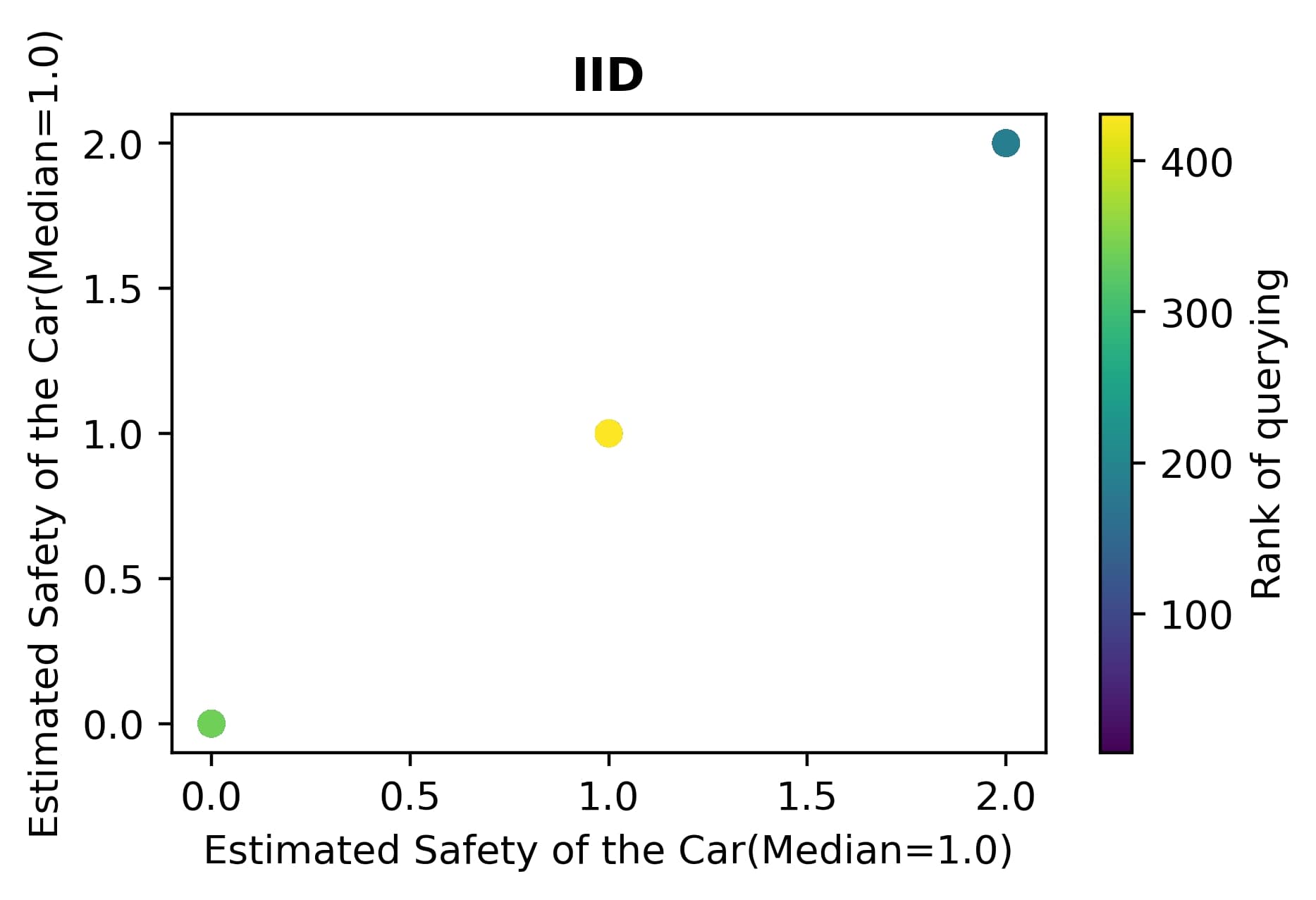

Supplement: Supplementary file 1 [file Data_Sheet_1.zip › Figures in Supplimentary Material/IID_Figure_3.jpg]

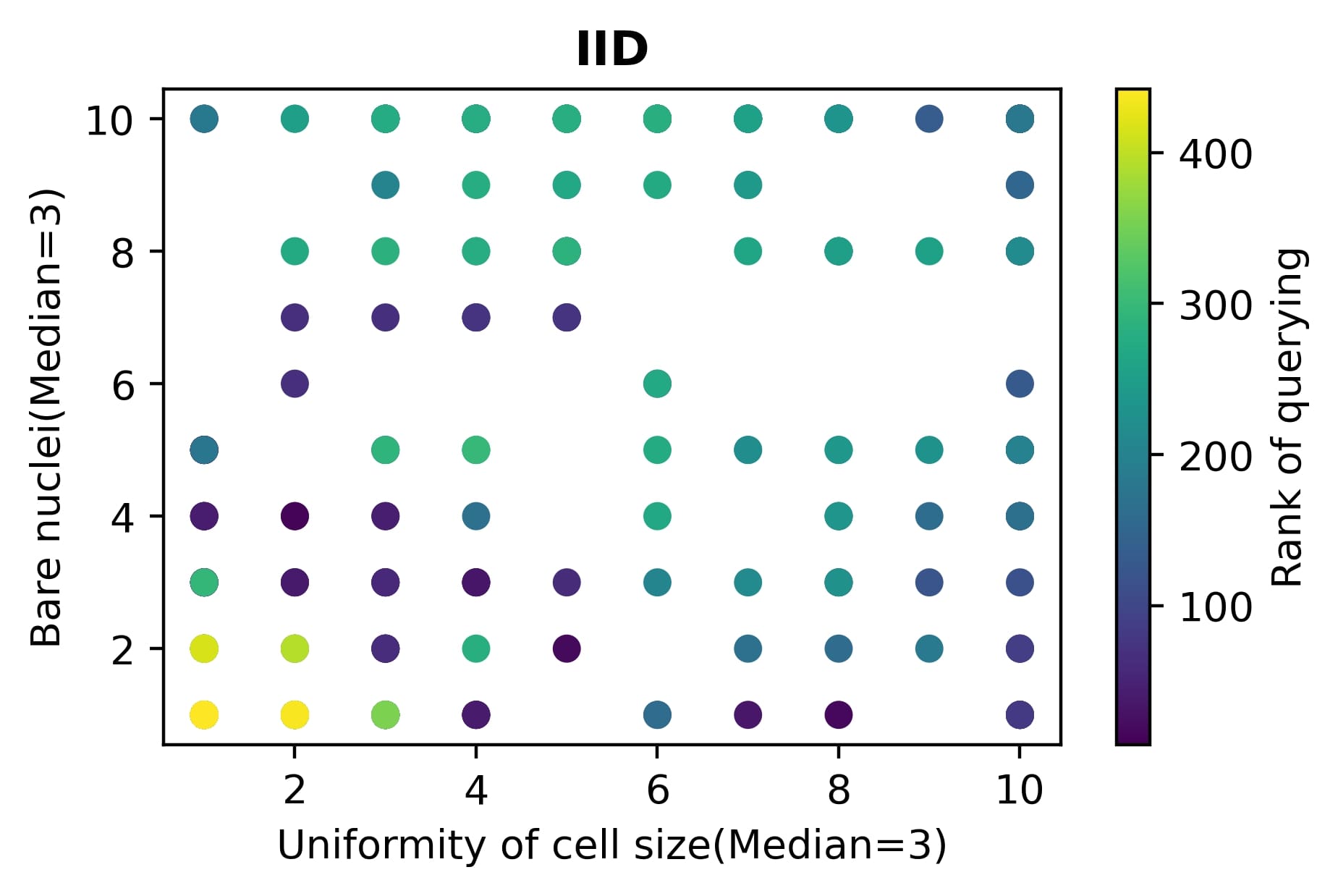

Supplement: Supplementary file 1 [file Data_Sheet_1.zip › Figures in Supplimentary Material/IID_Figure_4.jpg]

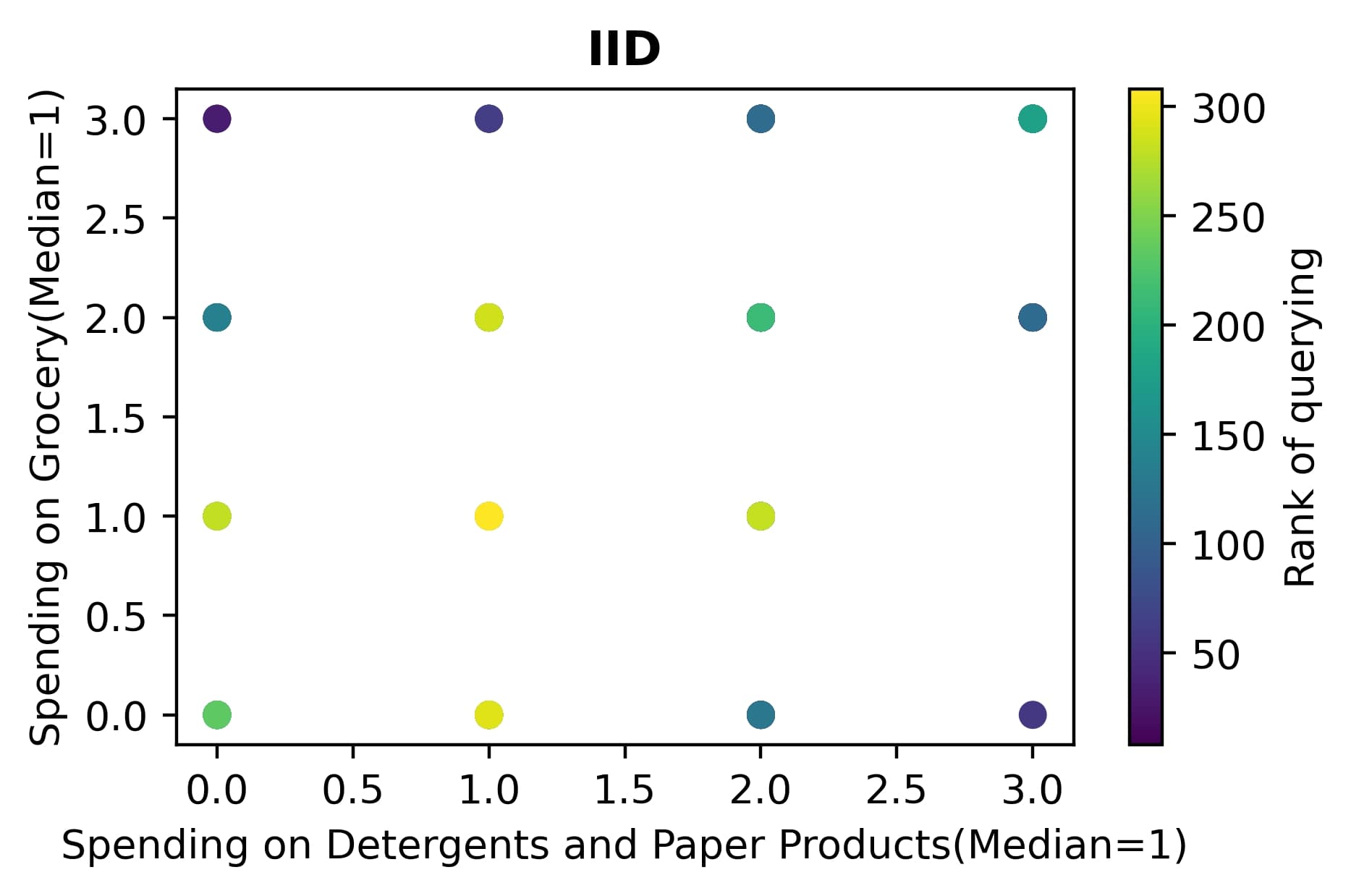

Supplement: Supplementary file 1 [file Data_Sheet_1.zip › Figures in Supplimentary Material/IID_Figure_5.jpg]

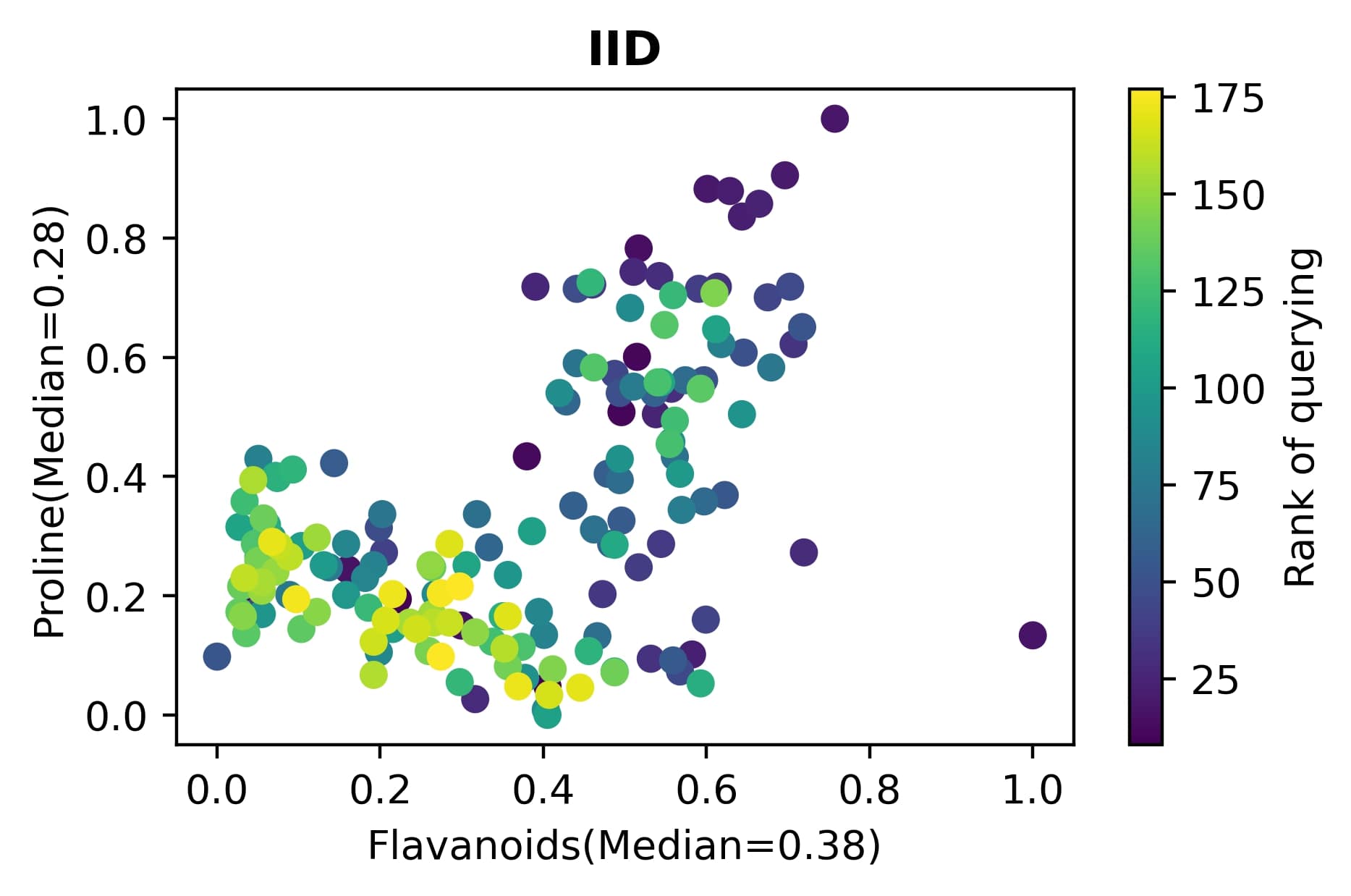

Supplement: Supplementary file 1 [file Data_Sheet_1.zip › Figures in Supplimentary Material/IID_Figure_6.jpg]

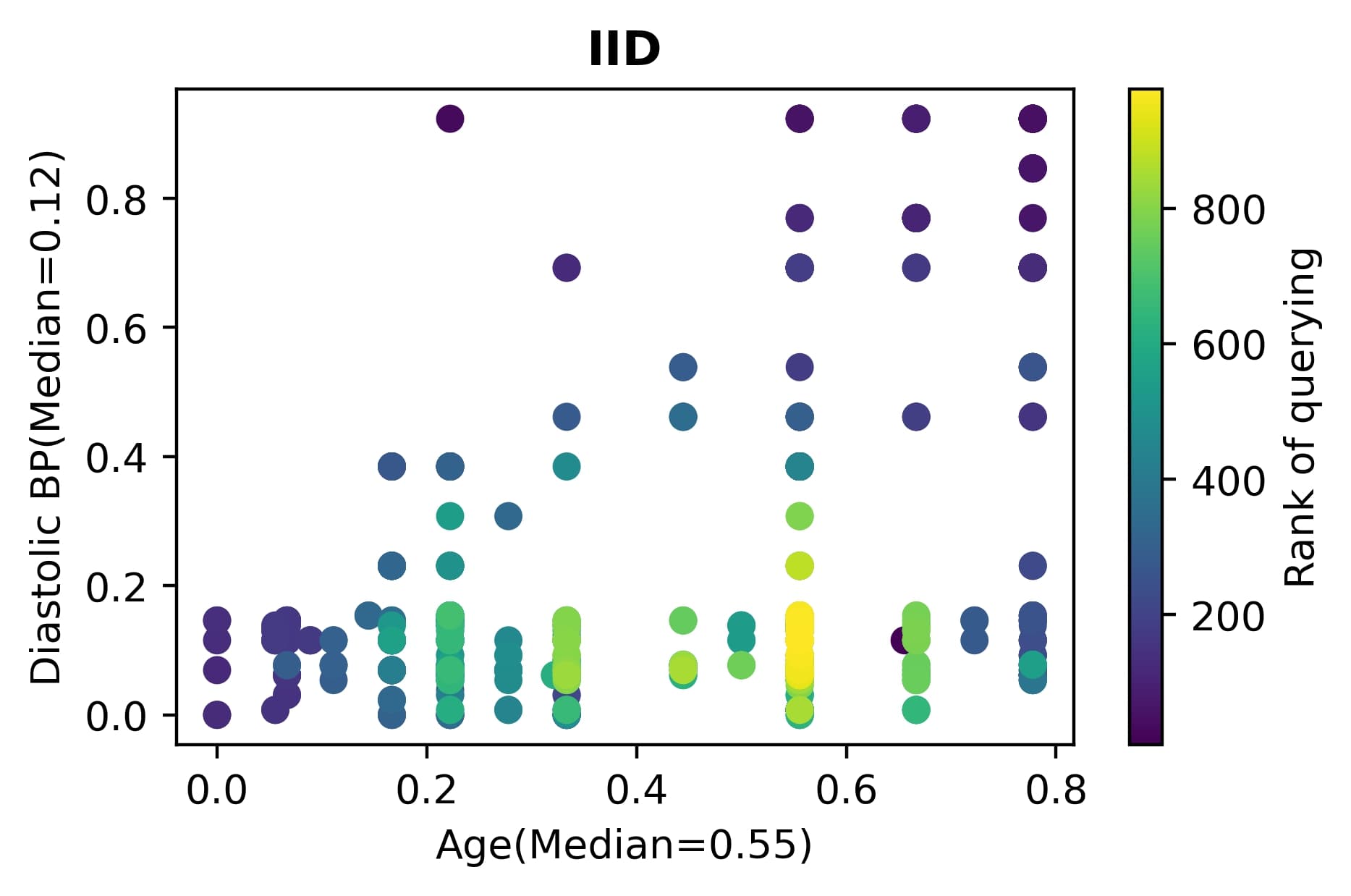

Supplement: Supplementary file 1 [file Data_Sheet_1.zip › Figures in Supplimentary Material/IID_Figure_7.jpg]

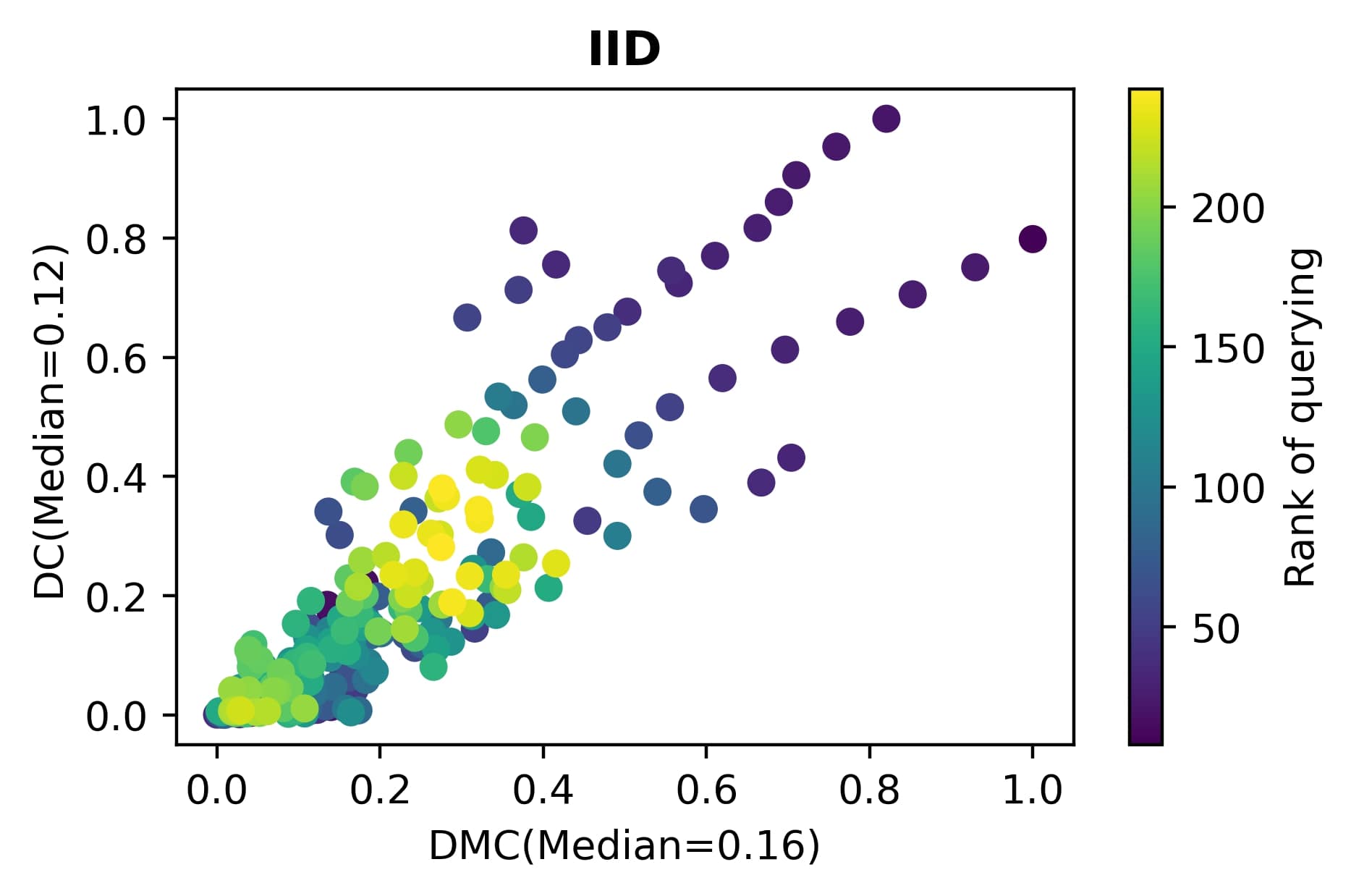

Supplement: Supplementary file 1 [file Data_Sheet_1.zip › Figures in Supplimentary Material/IID_Figure_8.jpg]

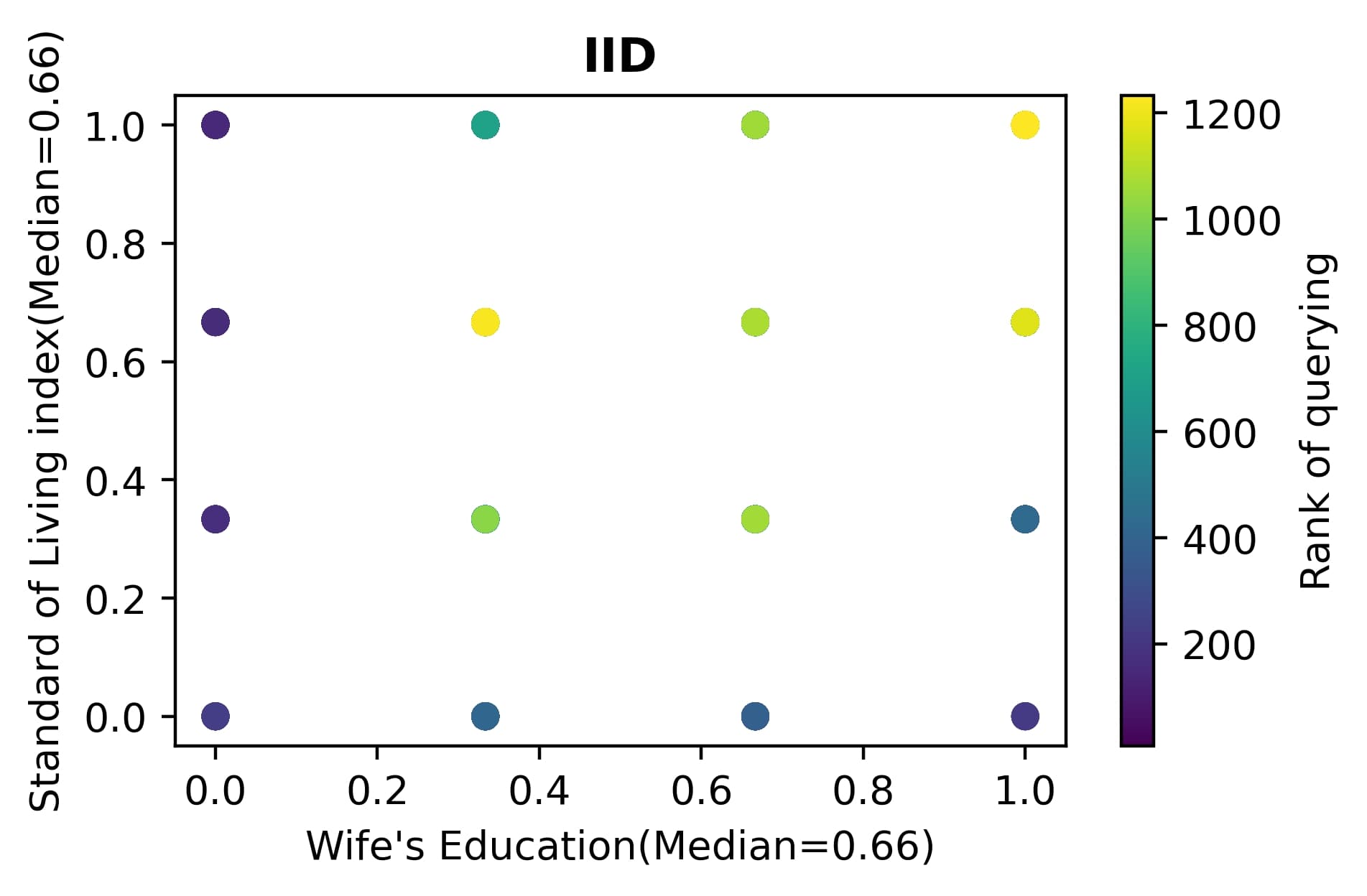

Supplement: Supplementary file 1 [file Data_Sheet_1.zip › Figures in Supplimentary Material/IID_Figure_9.jpg]

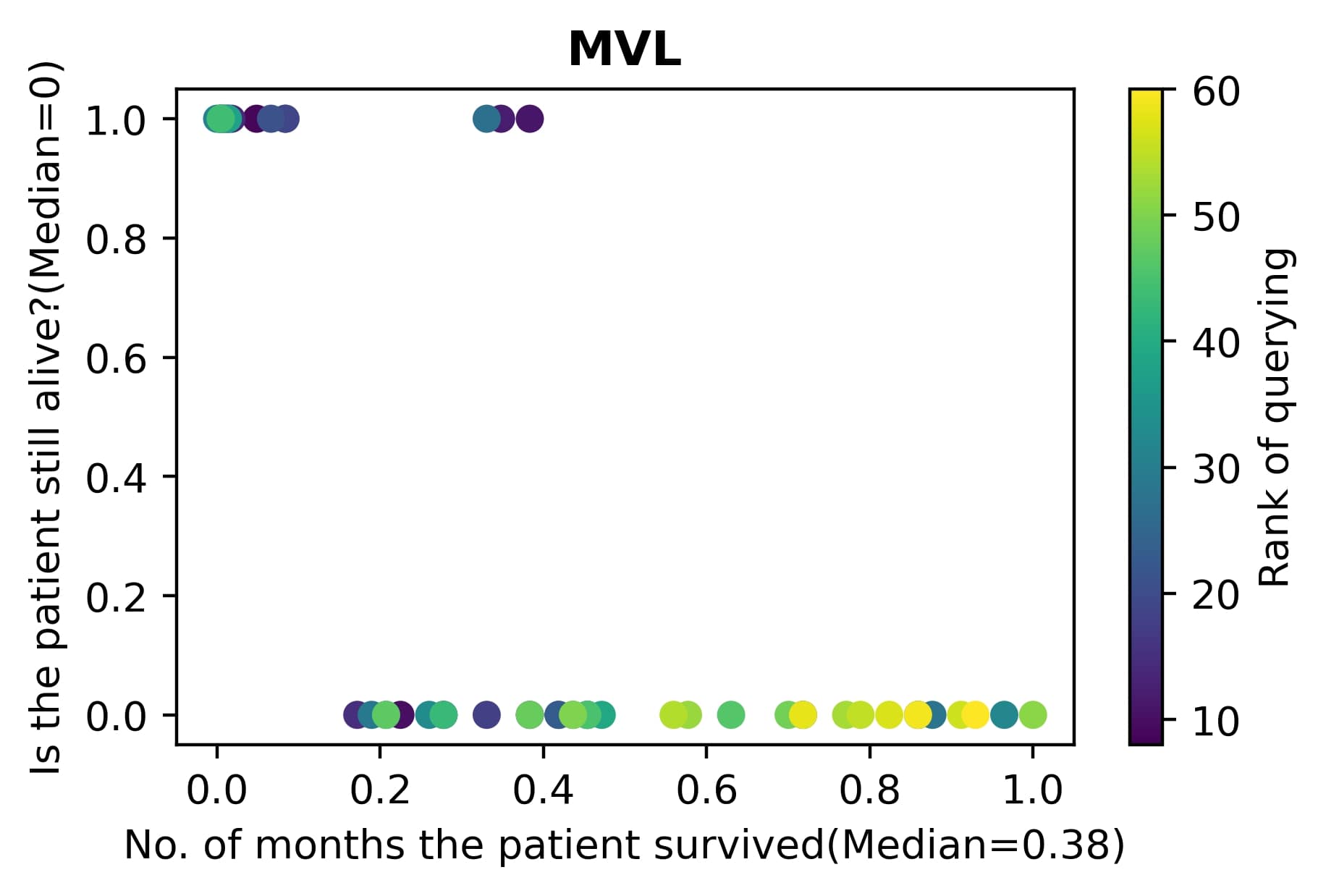

Supplement: Supplementary file 1 [file Data_Sheet_1.zip › Figures in Supplimentary Material/MVL_Figure_10.jpg]

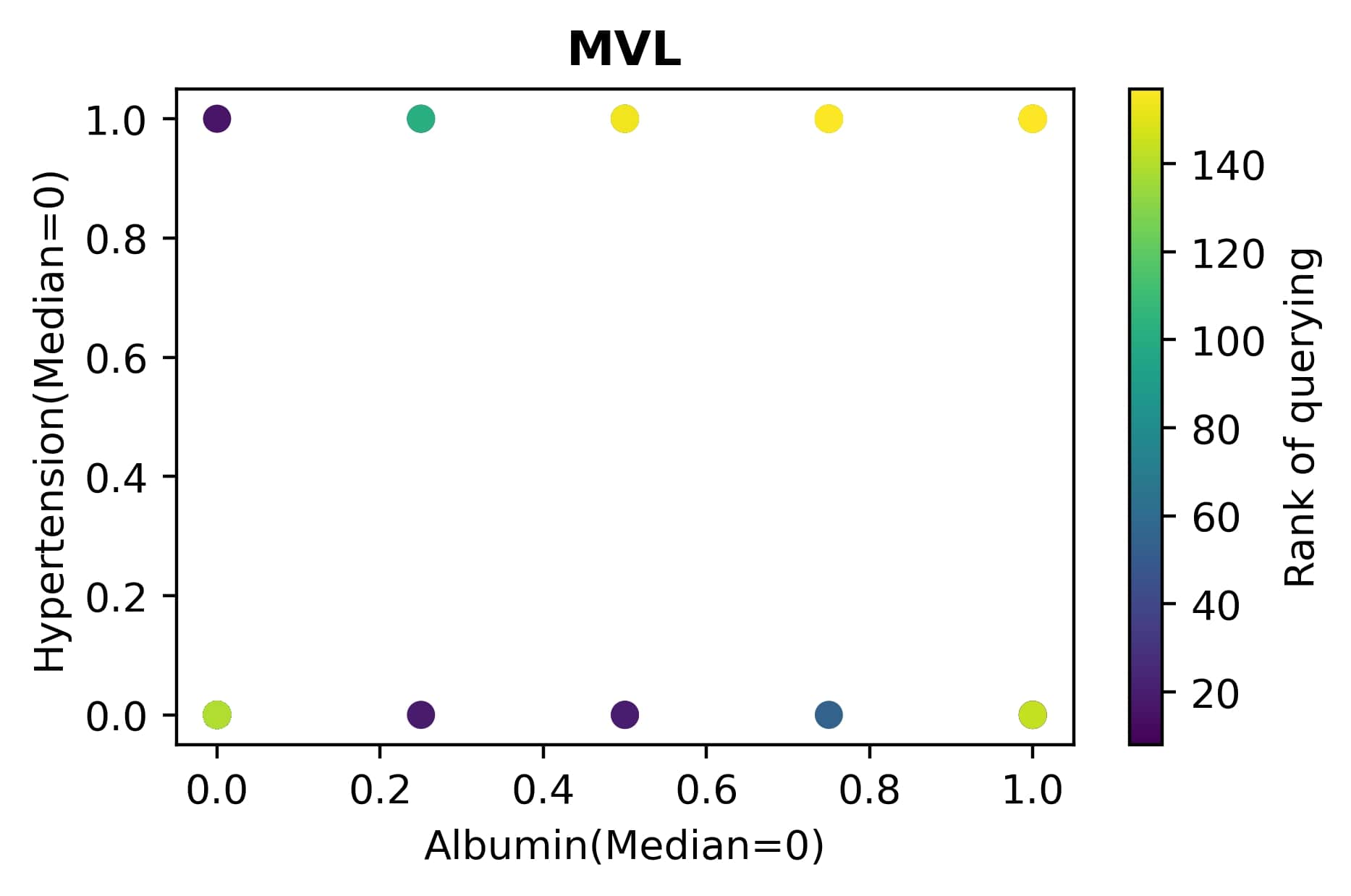

Supplement: Supplementary file 1 [file Data_Sheet_1.zip › Figures in Supplimentary Material/MVL_Figure_11.jpg]

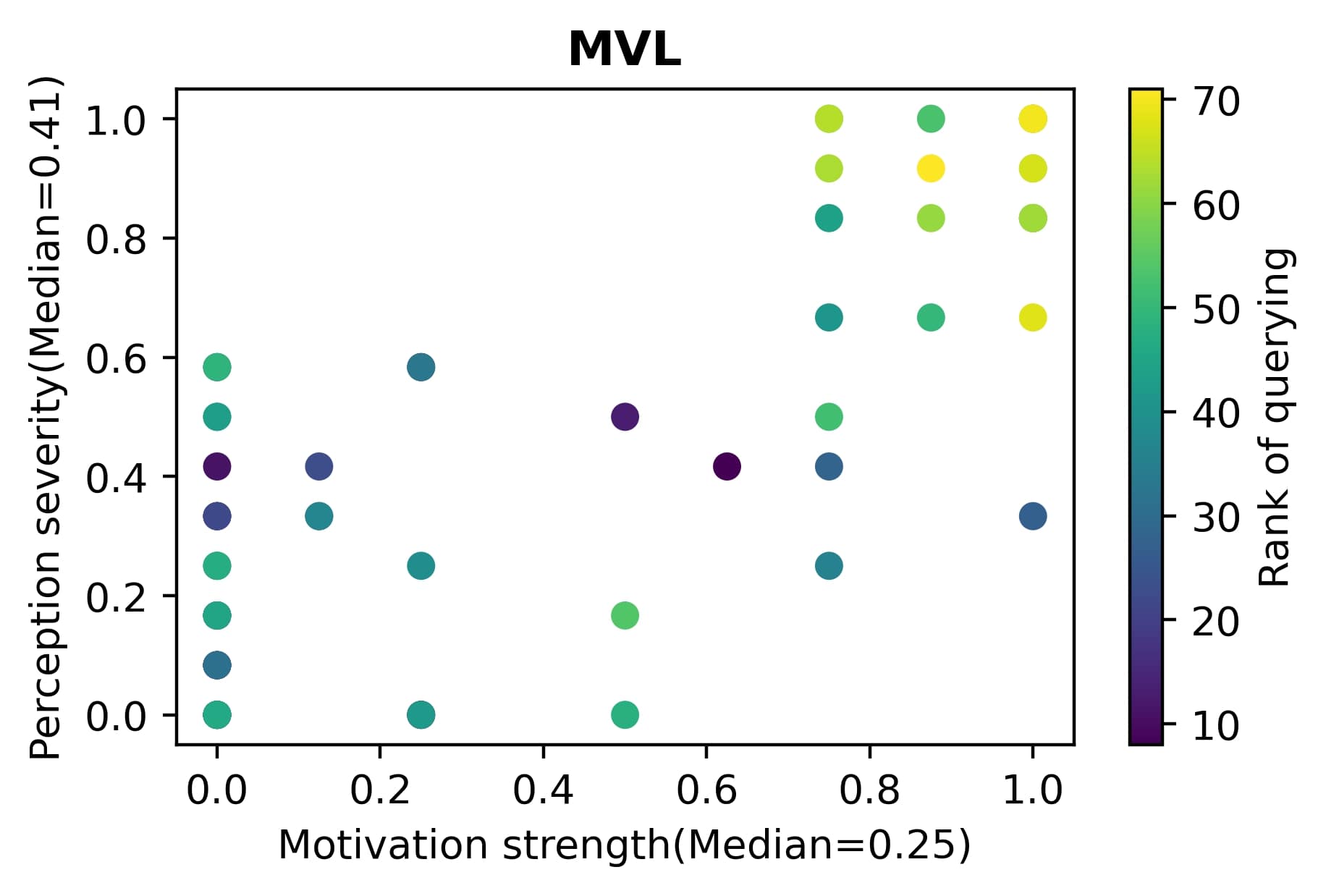

Supplement: Supplementary file 1 [file Data_Sheet_1.zip › Figures in Supplimentary Material/MVL_Figure_12.jpg]

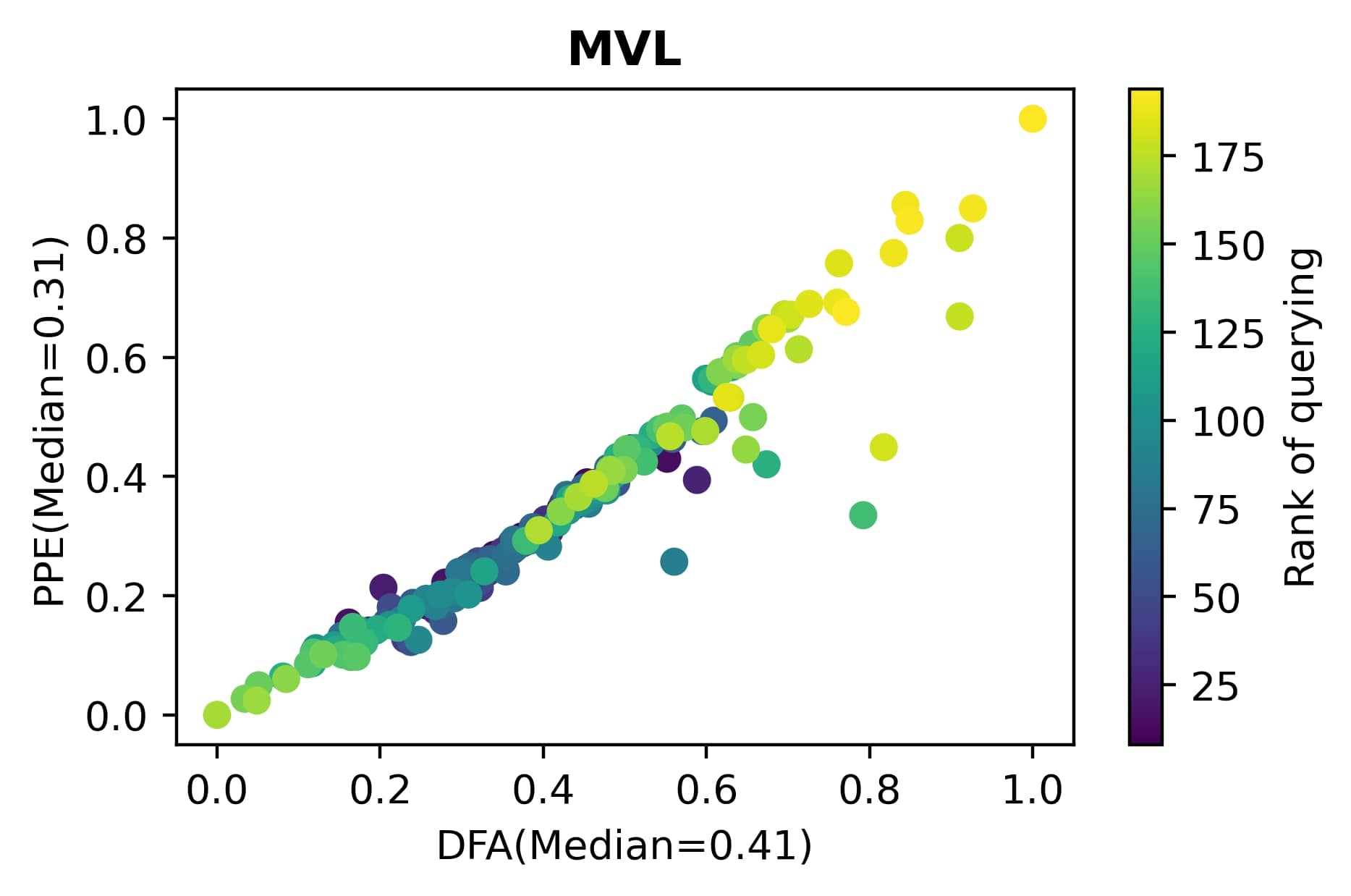

Supplement: Supplementary file 1 [file Data_Sheet_1.zip › Figures in Supplimentary Material/MVL_Figure_13.jpg]

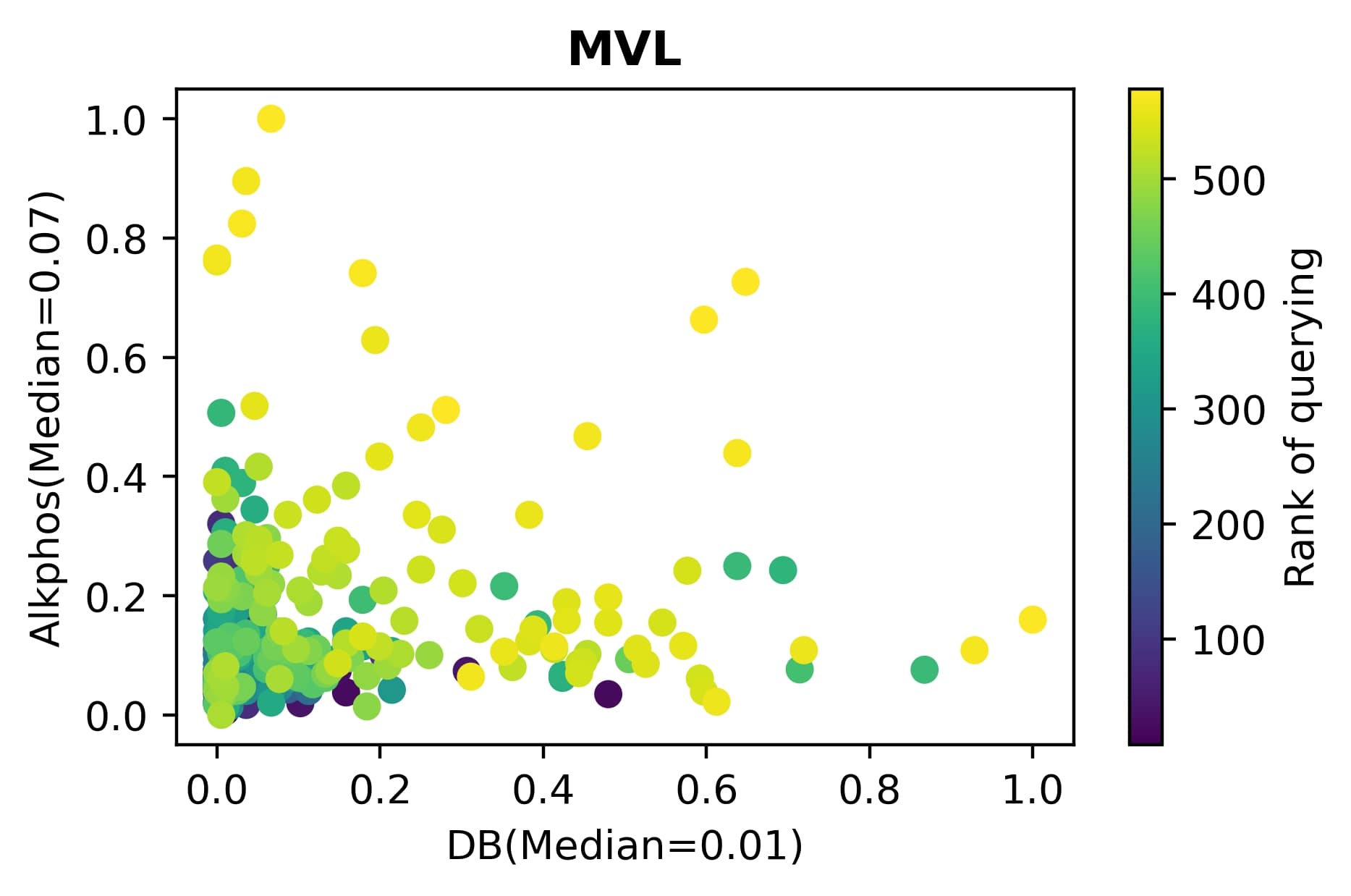

Supplement: Supplementary file 1 [file Data_Sheet_1.zip › Figures in Supplimentary Material/MVL_Figure_14.jpg]

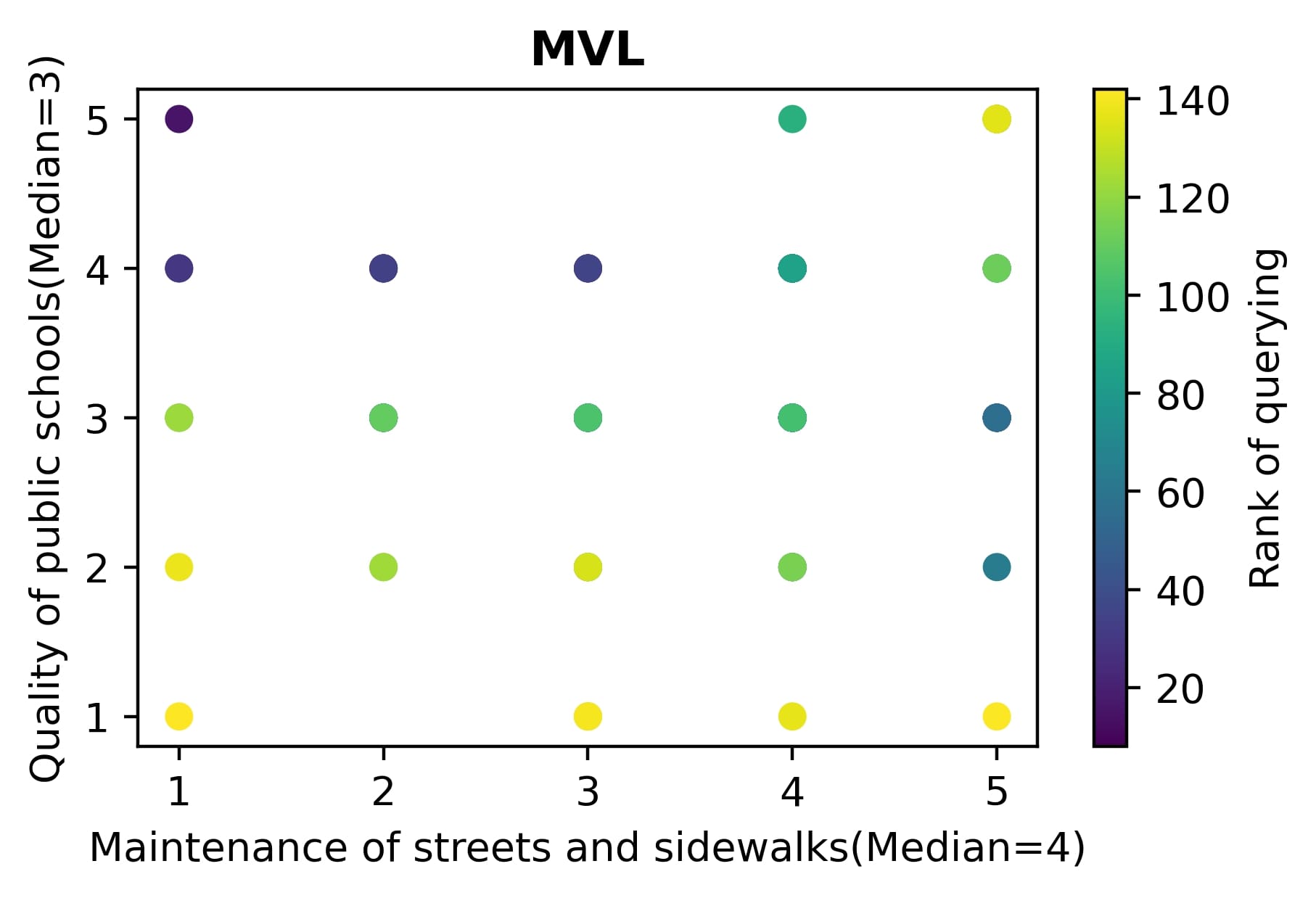

Supplement: Supplementary file 1 [file Data_Sheet_1.zip › Figures in Supplimentary Material/MVL_Figure_15.jpg]

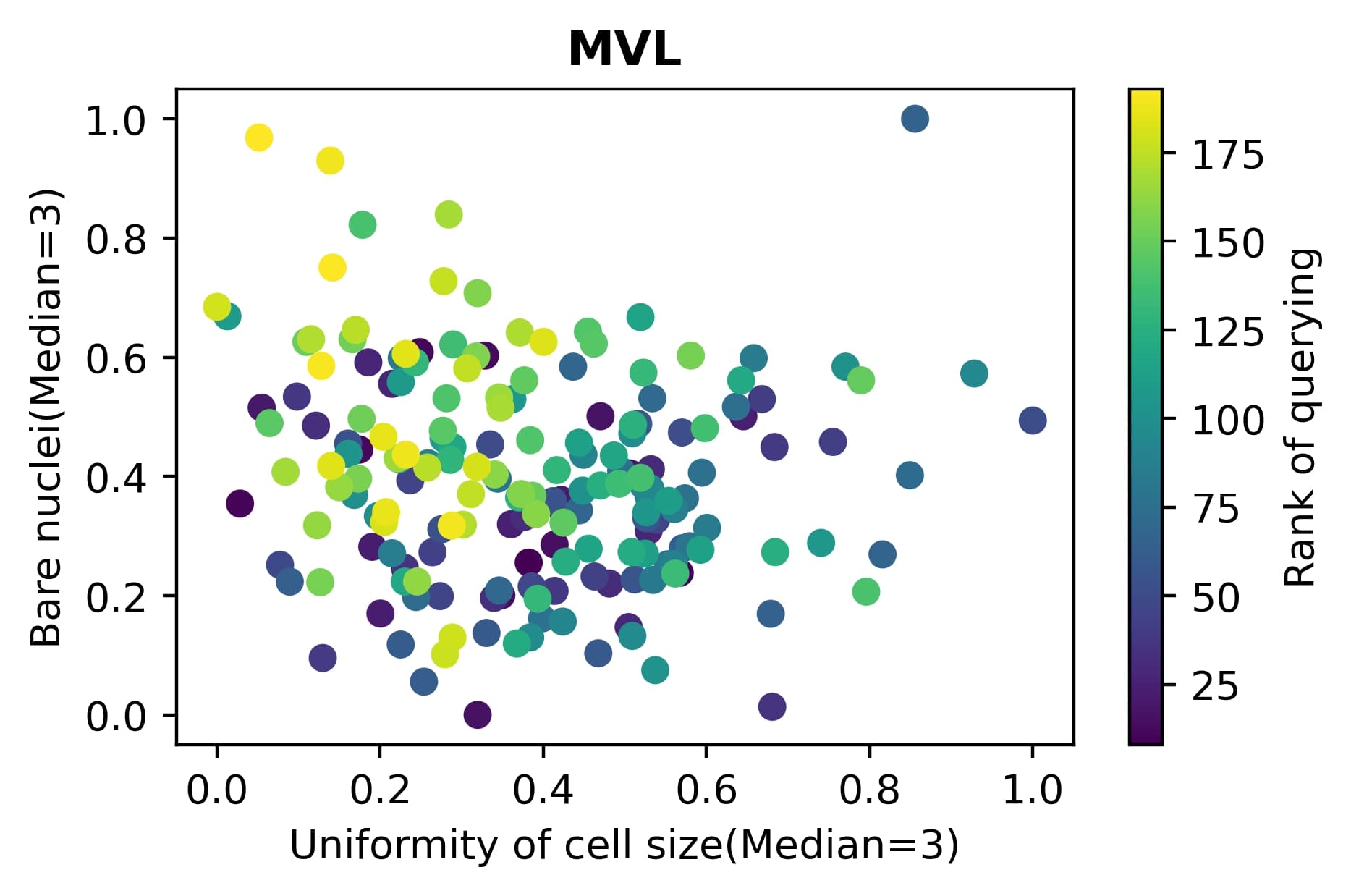

Supplement: Supplementary file 1 [file Data_Sheet_1.zip › Figures in Supplimentary Material/MVL_Figure_16.jpg]

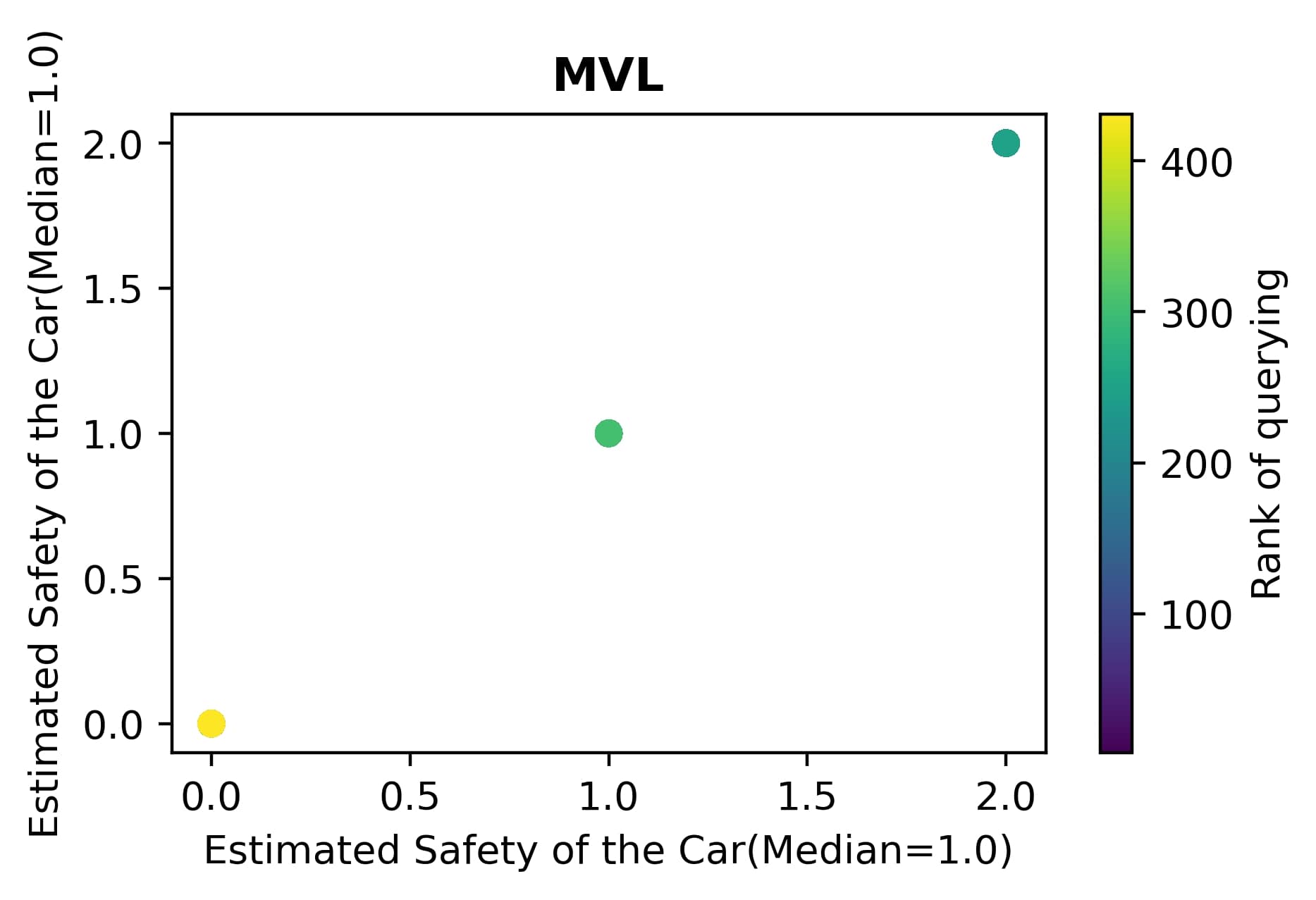

Supplement: Supplementary file 1 [file Data_Sheet_1.zip › Figures in Supplimentary Material/MVL_Figure_3.jpg]

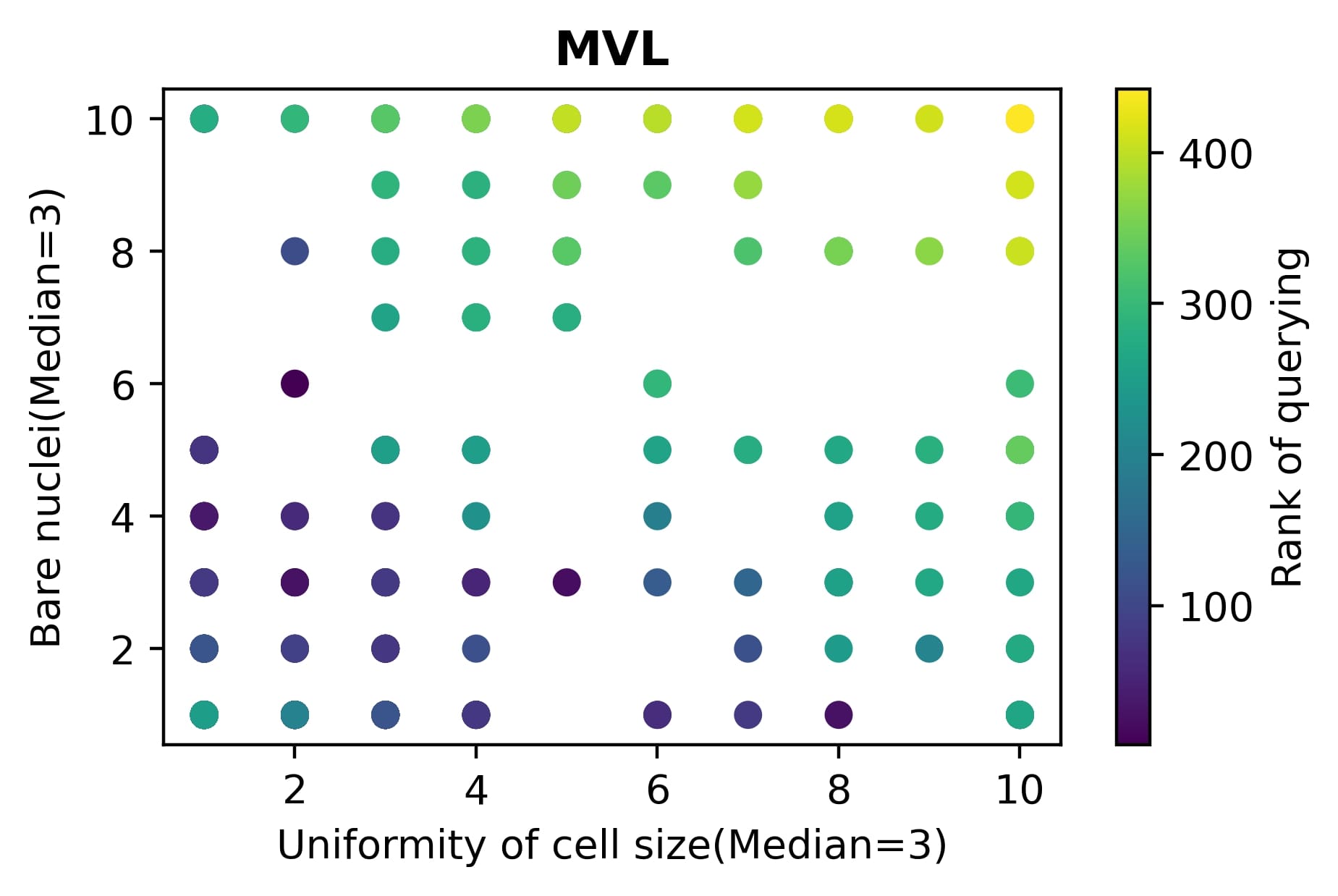

Supplement: Supplementary file 1 [file Data_Sheet_1.zip › Figures in Supplimentary Material/MVL_Figure_4.jpg]

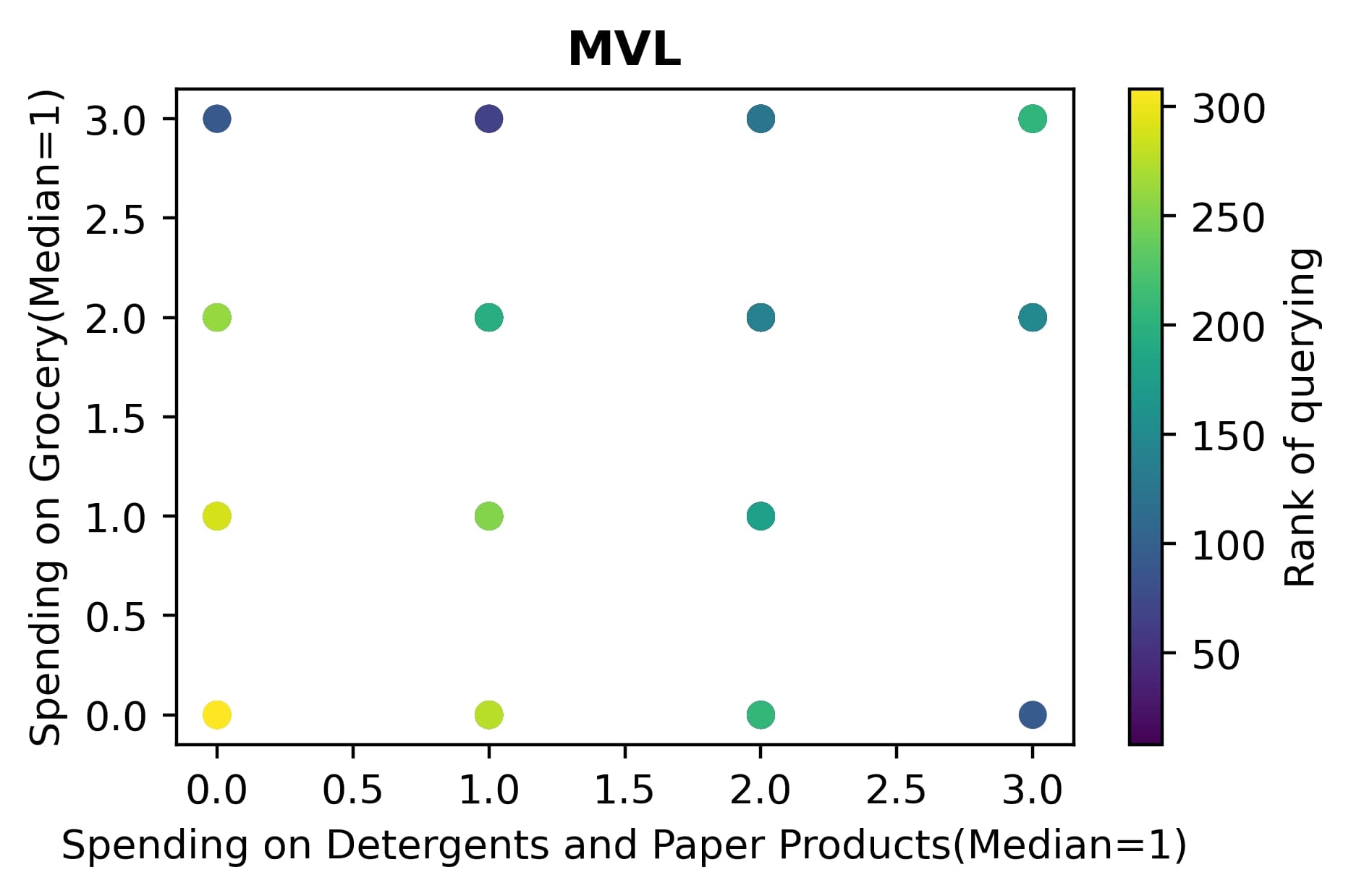

Supplement: Supplementary file 1 [file Data_Sheet_1.zip › Figures in Supplimentary Material/MVL_Figure_5.jpg]

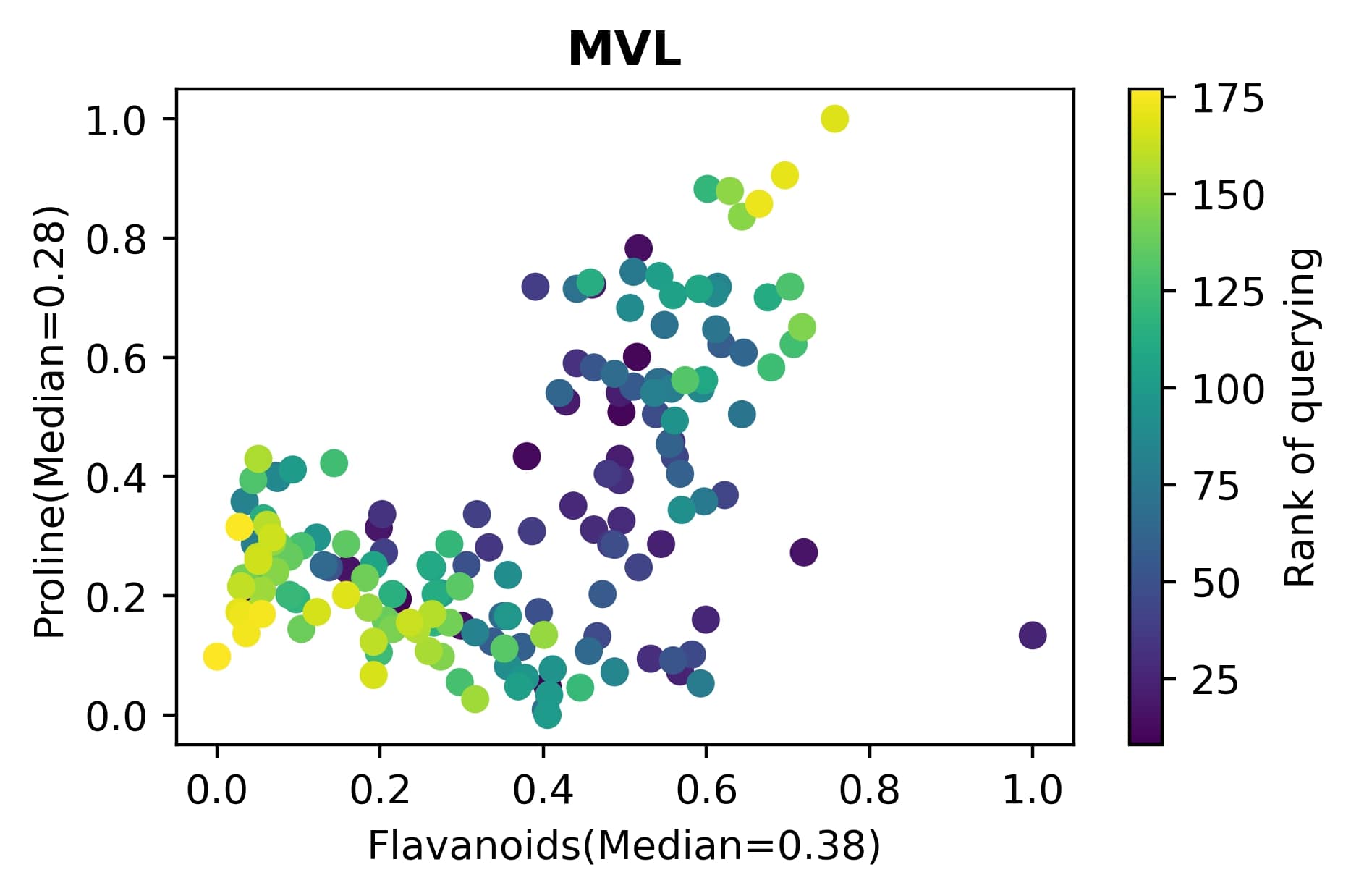

Supplement: Supplementary file 1 [file Data_Sheet_1.zip › Figures in Supplimentary Material/MVL_Figure_6.jpg]

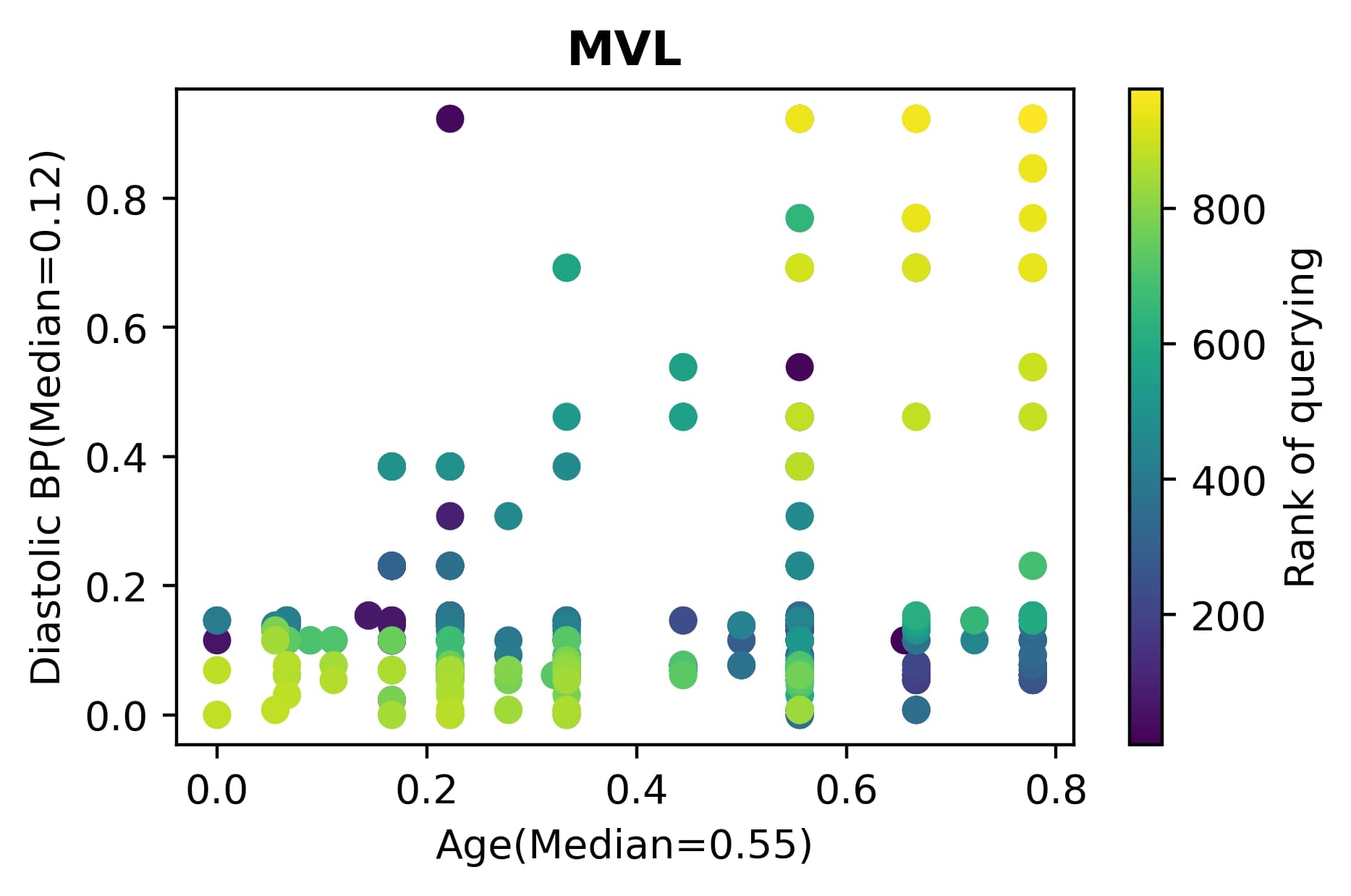

Supplement: Supplementary file 1 [file Data_Sheet_1.zip › Figures in Supplimentary Material/MVL_Figure_7.jpg]

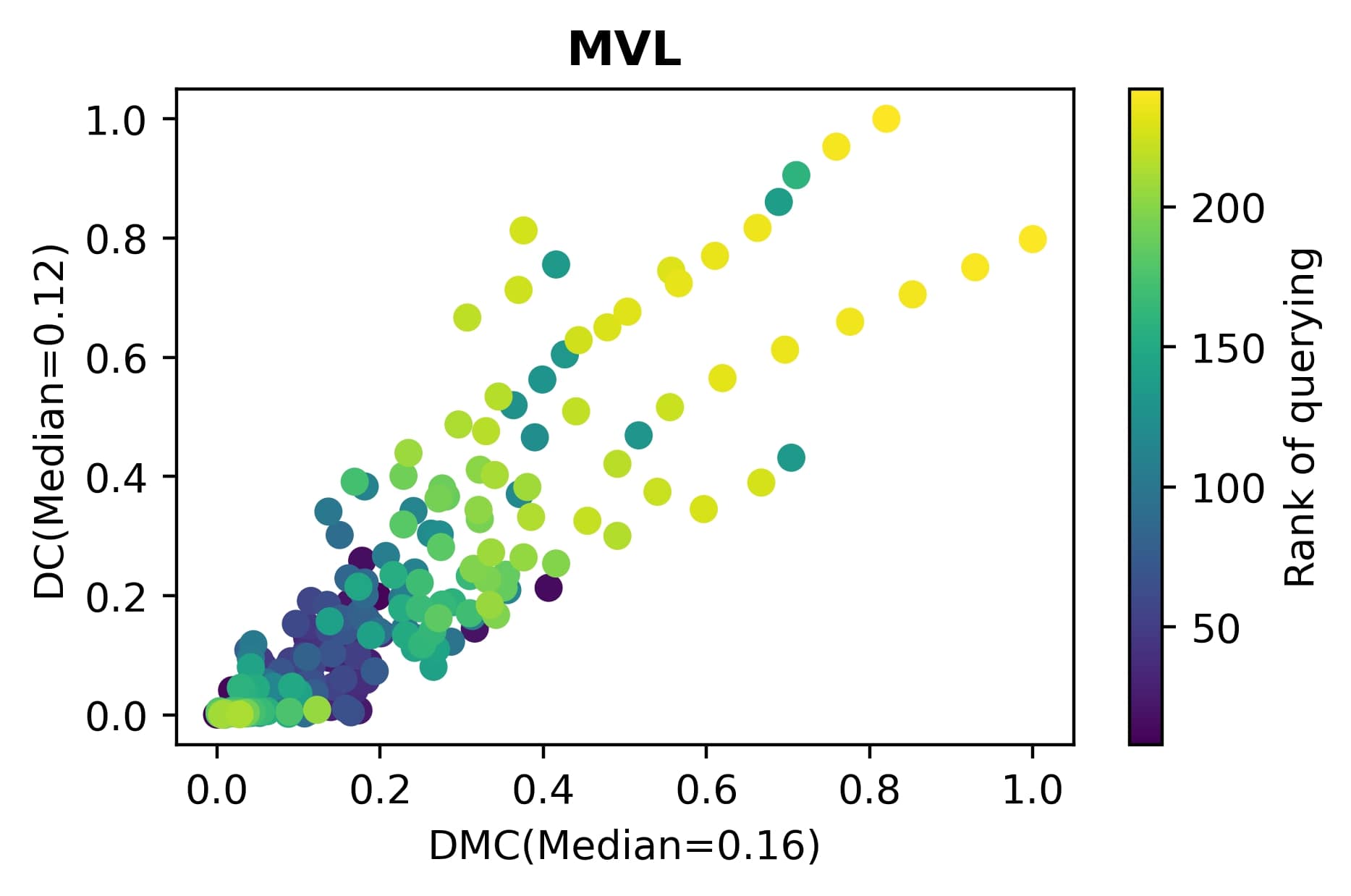

Supplement: Supplementary file 1 [file Data_Sheet_1.zip › Figures in Supplimentary Material/MVL_Figure_8.jpg]

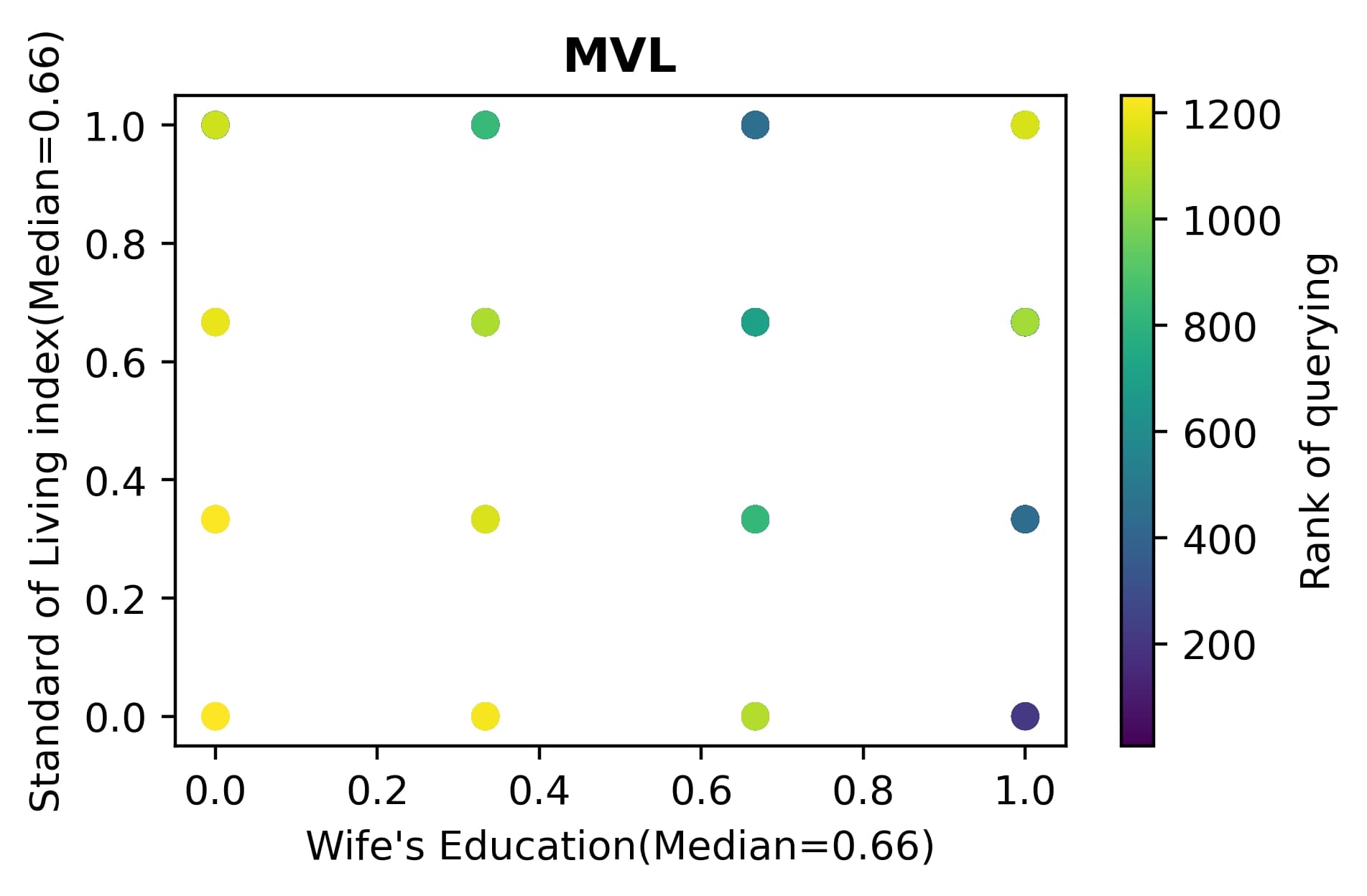

Supplement: Supplementary file 1 [file Data_Sheet_1.zip › Figures in Supplimentary Material/MVL_Figure_9.jpg]
