# Supplementary material for: Active learning with human heuristics: an algorithm robust to labeling bias
Source: Front Artif Intell. 2024 Nov 19;7:1491932. doi: 10.3389/frai.2024.1491932 (PMC11611880; doi:10.3389/frai.2024.1491932)
Supplement: Supplementary file 2 [file Data_Sheet_2.pdf]

Supplementary Material

1 SECTION A: DATASET DESCRIPTION

Sr.No.	Dataset name	No. of Attributes	Prediction task
1	Car Condition	6	Predict the condition of a Car based on its structural features, maintenance, and buying price
2	Breast cancer	9	Predict the recurrence of tumor based on relevant health information
3	Wholesale customer	7	Predict customer purchase channel based on annual spending on various products
4	Raisin	7	Predict type of raisin (Kecimen or Besni) based on their morphological features
5	Wine	12	Predict the class of Wine based on the quantity of their constituents
6	Maternal health	6	Predict the risk level of maternal mortality based on Age, BP measures, etc.
7	Algerian Forest	9	Predict occurrence of forest fire weather data observations
8	Contraceptive	8	Predict contraceptive usage of a woman based on her demographic and socio-economic characteristics.
9	ECG	11	Predict survival of a patient for at least one year following heart attack based on relevant health features
10	Chronic Kidney	24	Predict the presence of Chronic kidney disease based on Age, RBC count, etc.
11	Cervical cancer	19	Predict the presence of Cervical cancer based on demographic information, habits, and medical records
12	Parkinsons	22	Predict the presence of Parkinson's disease based on biomedical voice measurements
13	Indian liver patient	10	Predict the presence of liver disease based on demographic and medical information of people
14	Happiness survey	6	Predict happiness of people based on features relevant to places they reside
15	Breast cancer - prognostic	33	Predict recurrence of Breast cancer based on relevant health information

Table S1: Description of datasets considered for the study

2 SECTION B: THEORY SUPPORTING IID

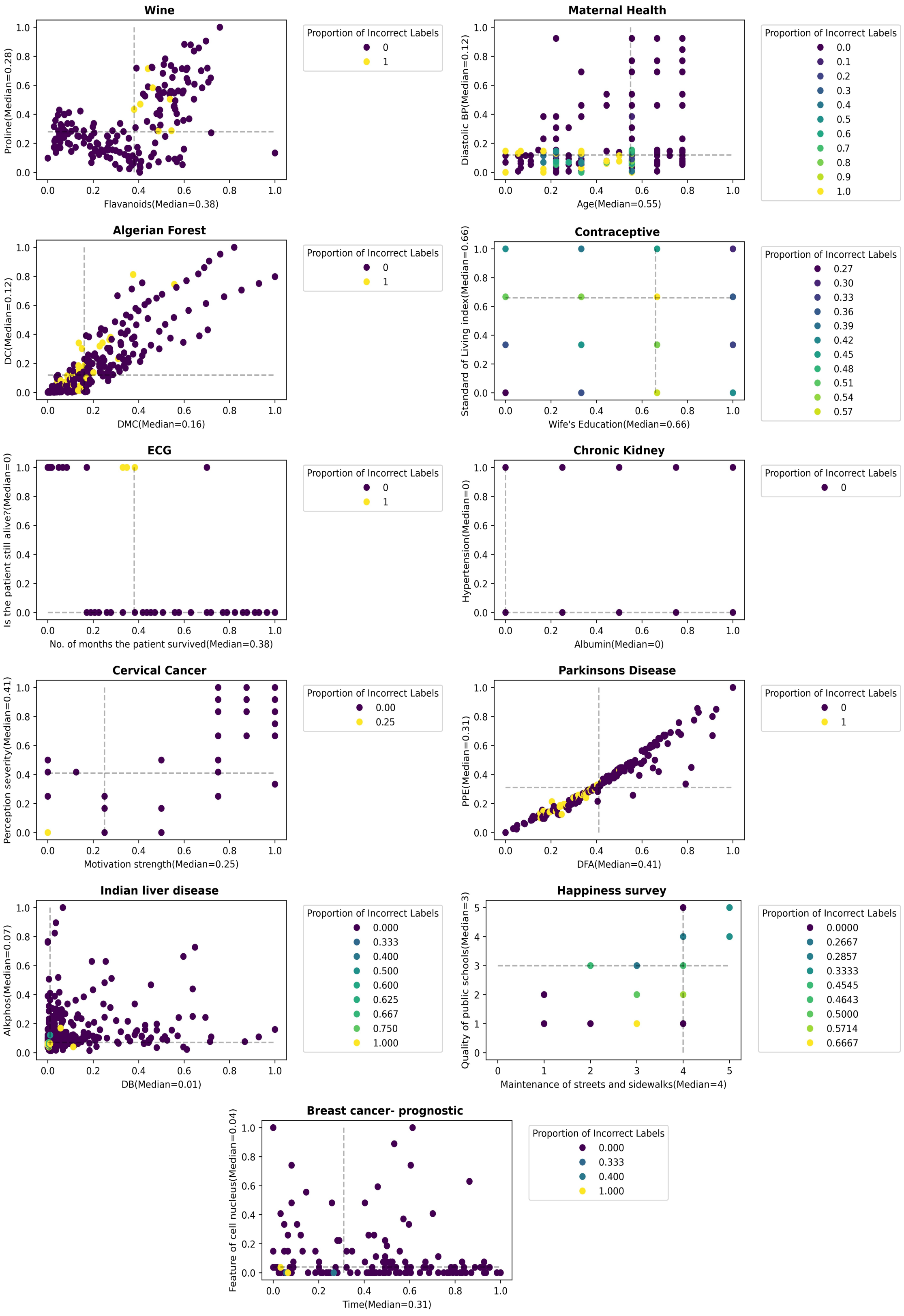

Figure S1: Analysis on predictions by the fast-and-frugal tree

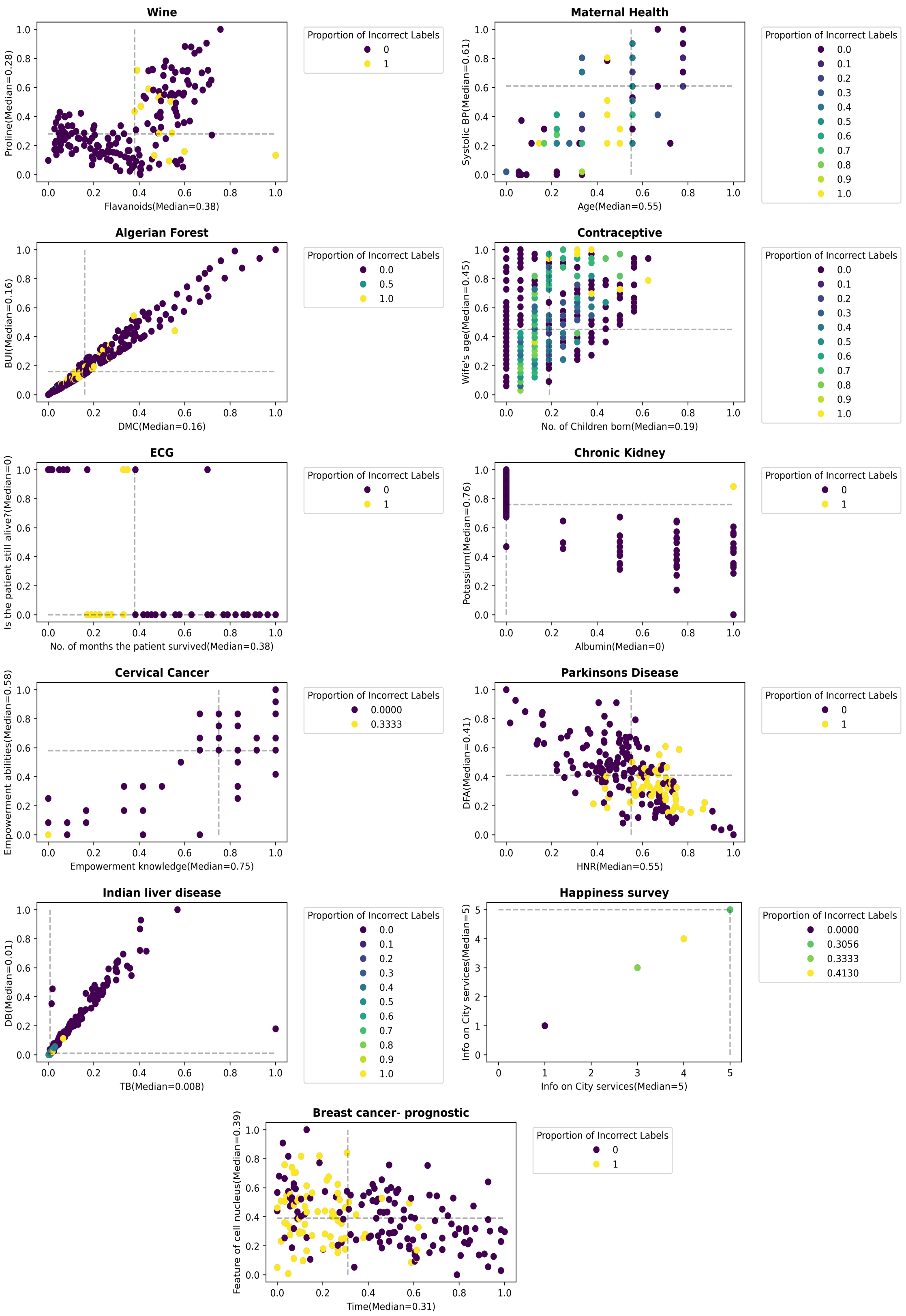

Figure S2: Analysis on predictions by the tallying heuristic

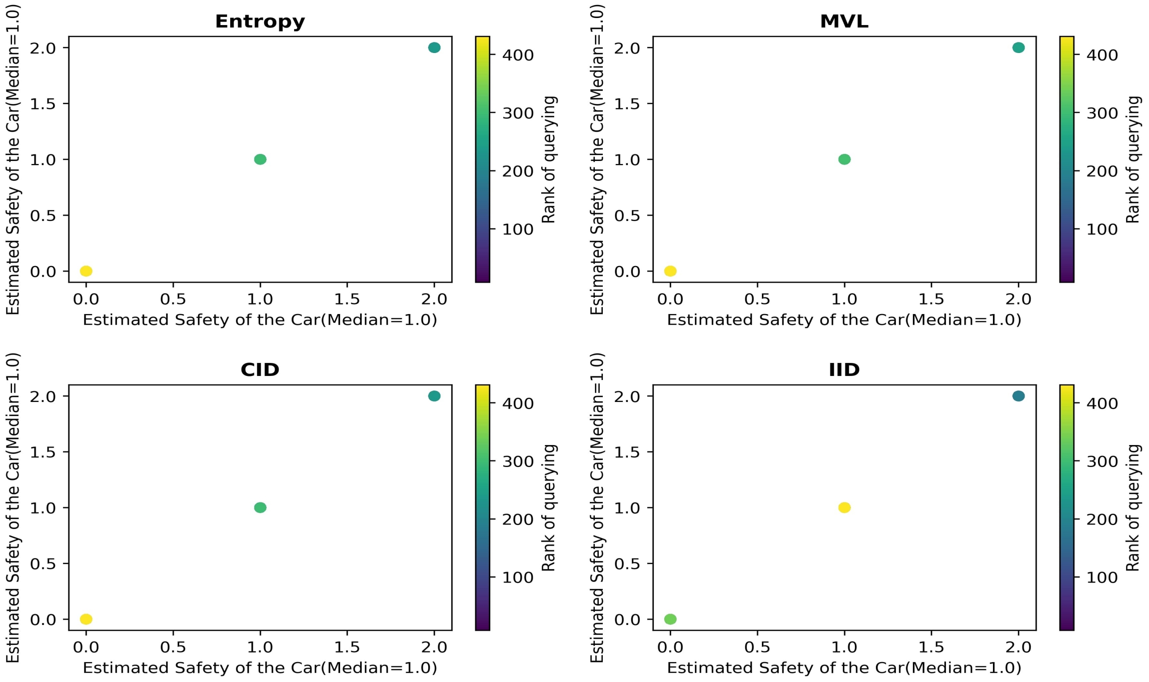

Figure S3: Querying order exhibited by AL algorithms in Car condition dataset

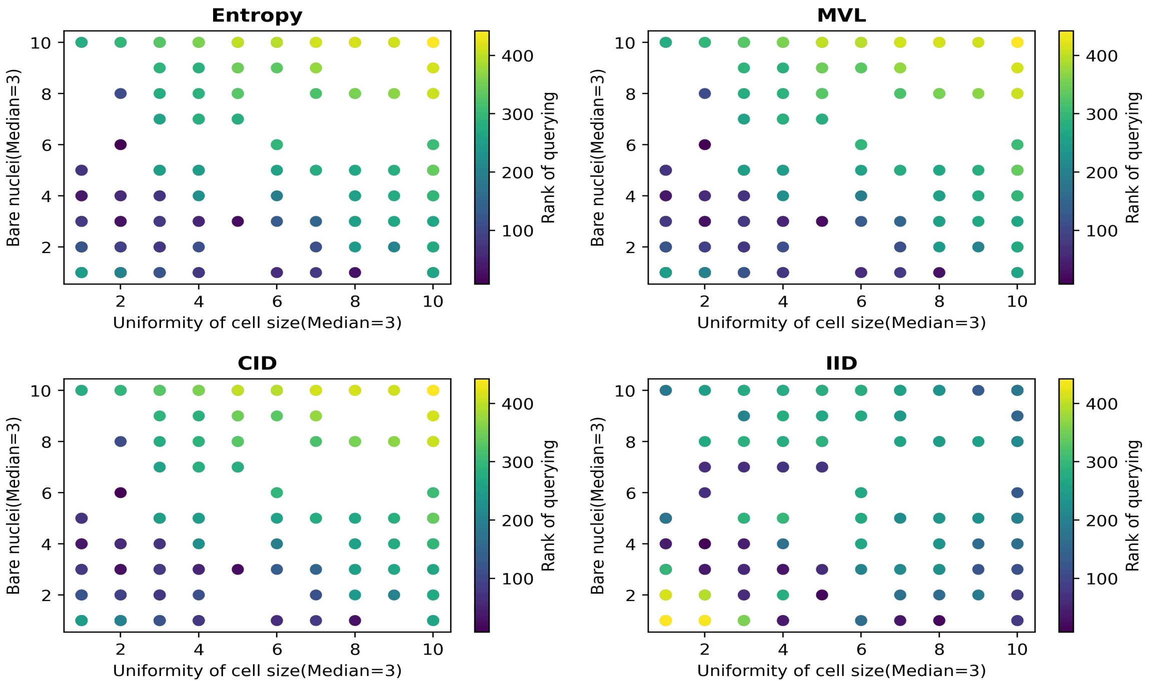

Figure S4: Querying order exhibited by AL algorithms in Breast cancer dataset

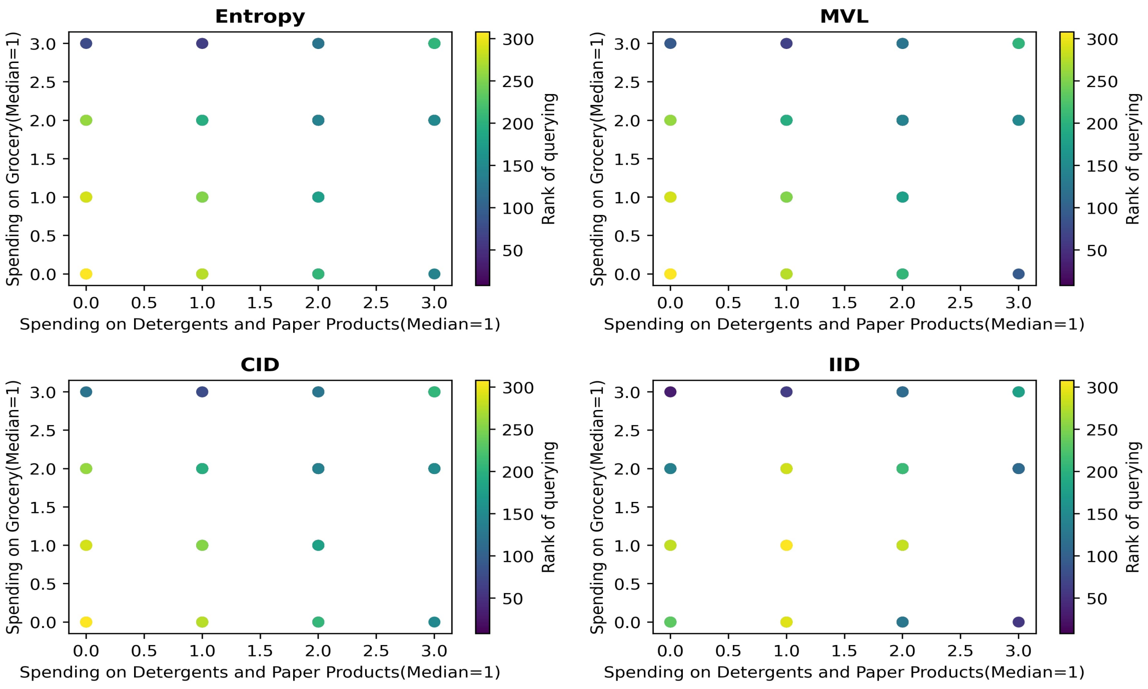

Figure S5: Querying order exhibited by AL algorithms in Wholesale customer dataset

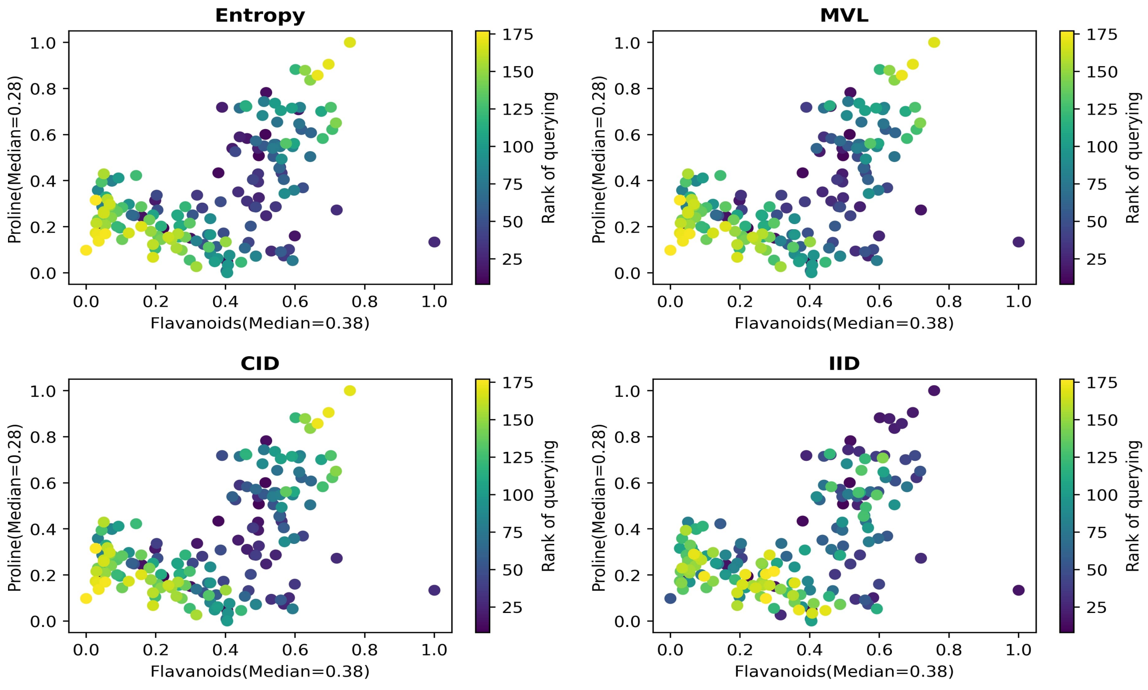

Figure S6: Querying order exhibited by AL algorithms in Wine dataset

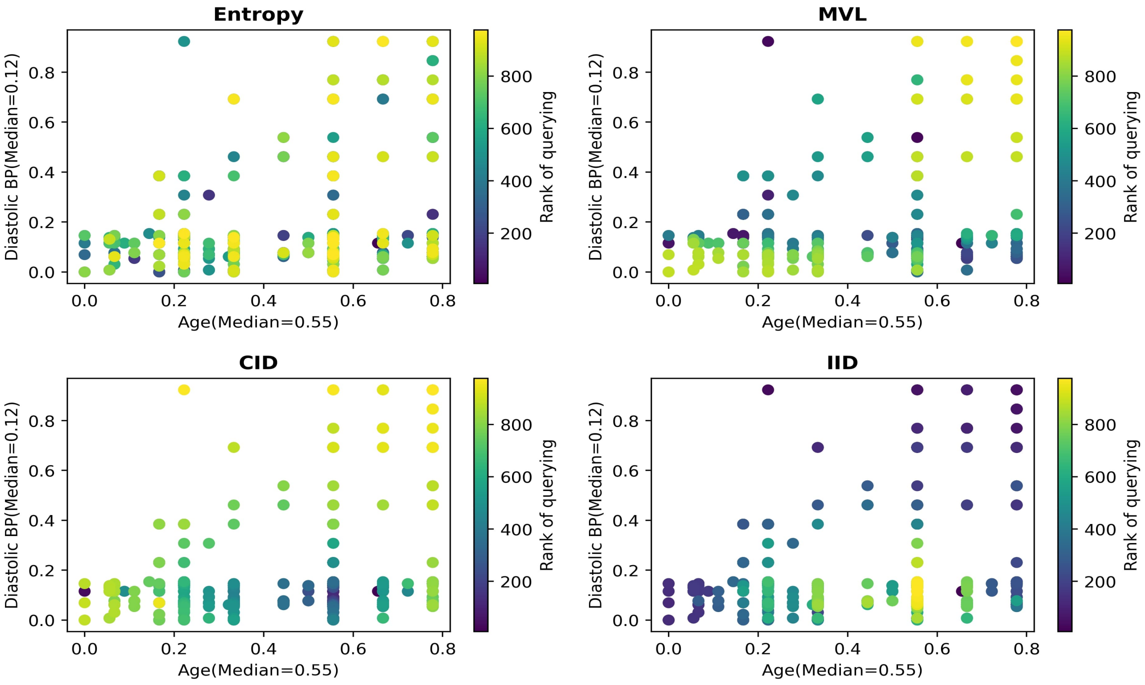

Figure S7: Querying order exhibited by AL algorithms in Maternal health dataset

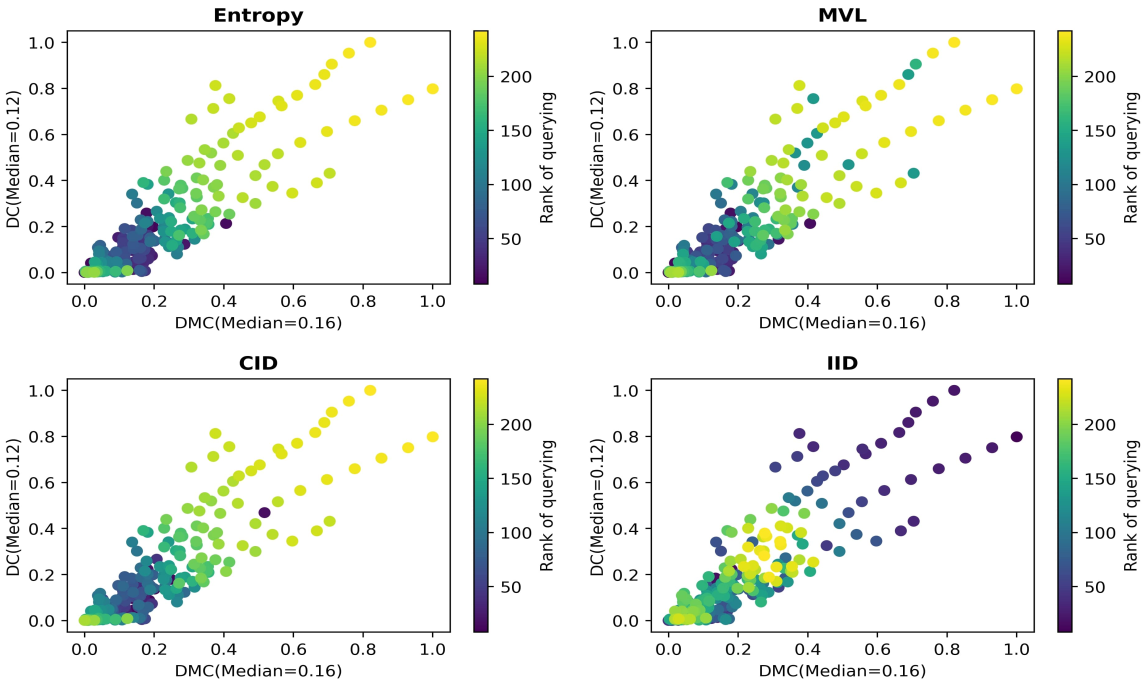

Figure S8: Querying order exhibited by AL algorithms in Algerian dataset

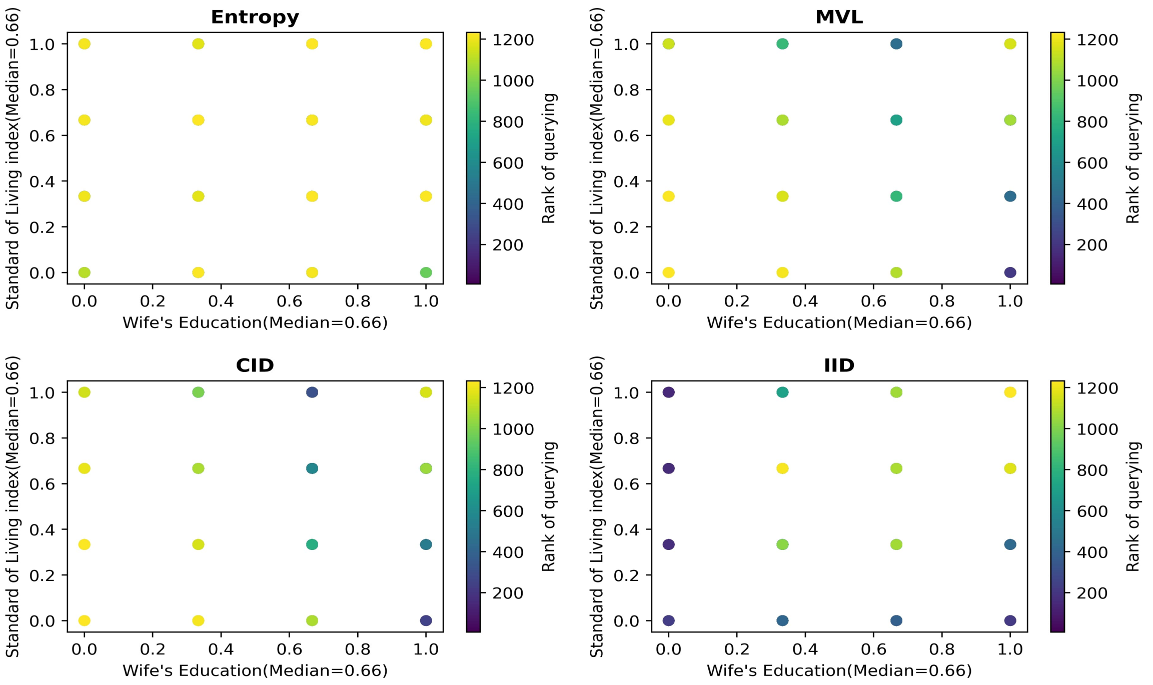

Figure S9: Querying order exhibited by AL algorithms in Contraceptive dataset

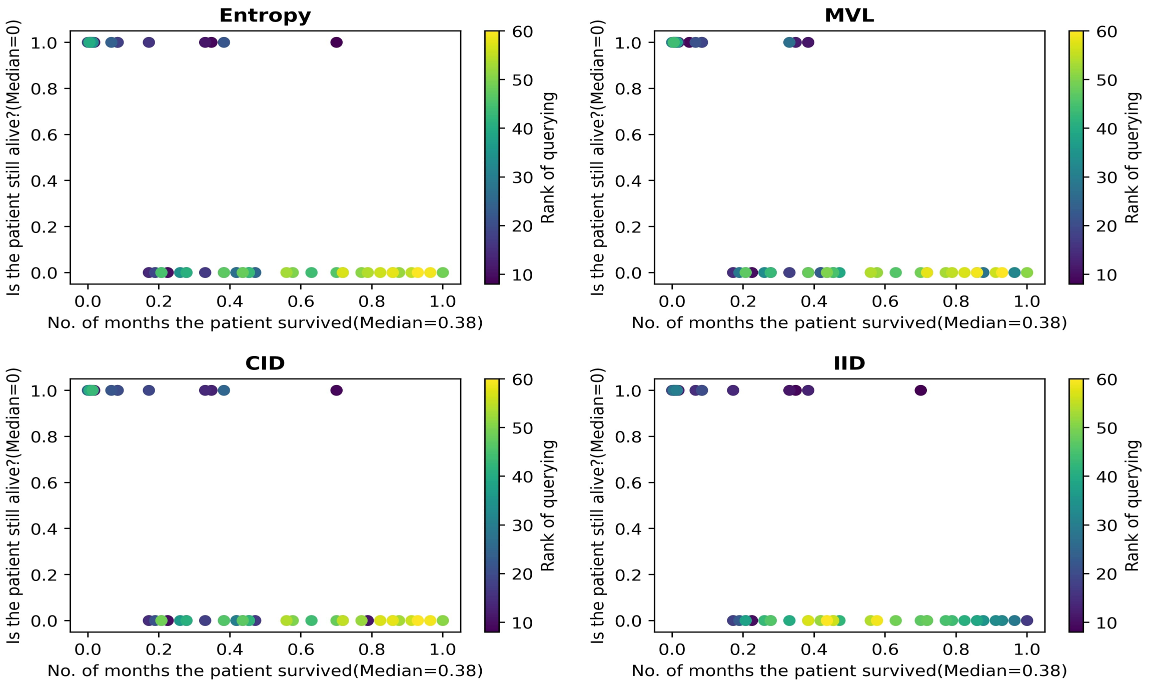

Figure S10: Querying order exhibited by AL algorithms in ECG dataset

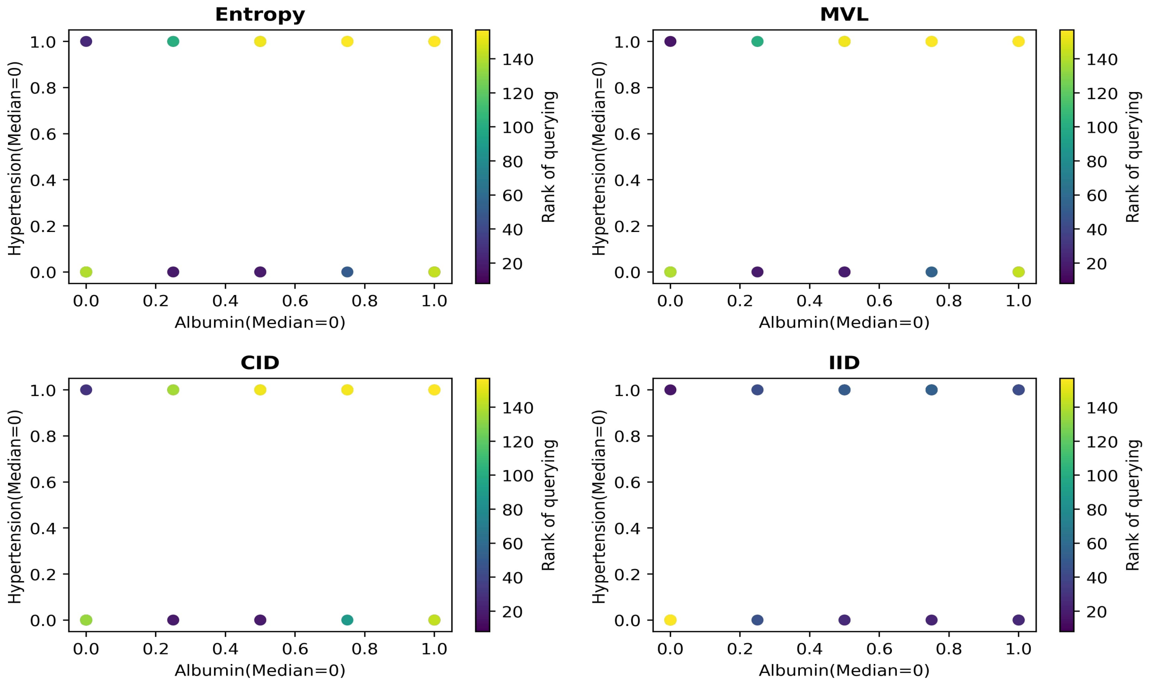

Figure S11: Querying order exhibited by AL algorithms in Chronic Kidney dataset

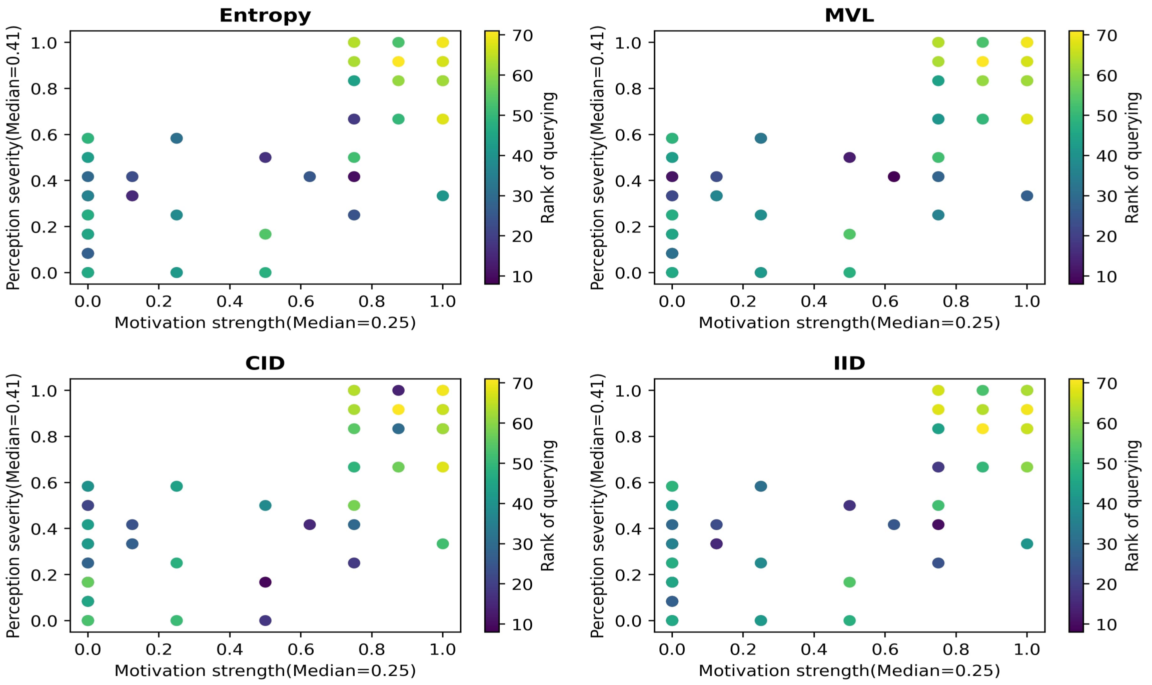

Figure S12: Querying order exhibited by AL algorithms in Cervical cancer dataset

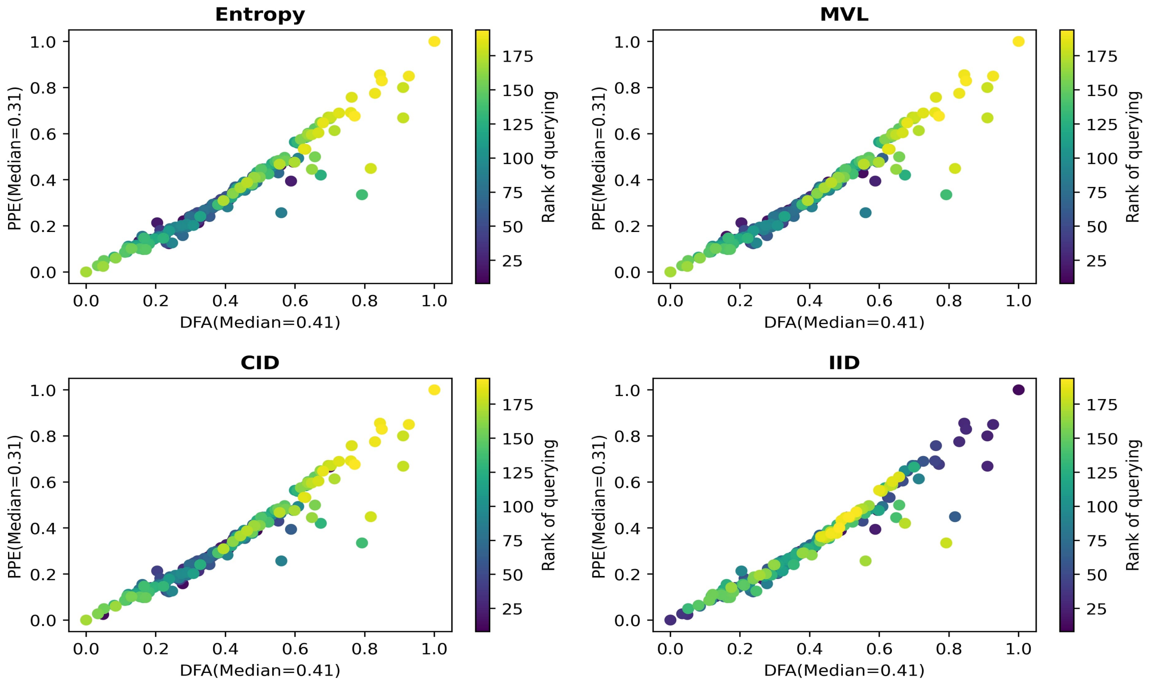

Figure S13: Querying order exhibited by AL algorithms in Parkinson's dataset

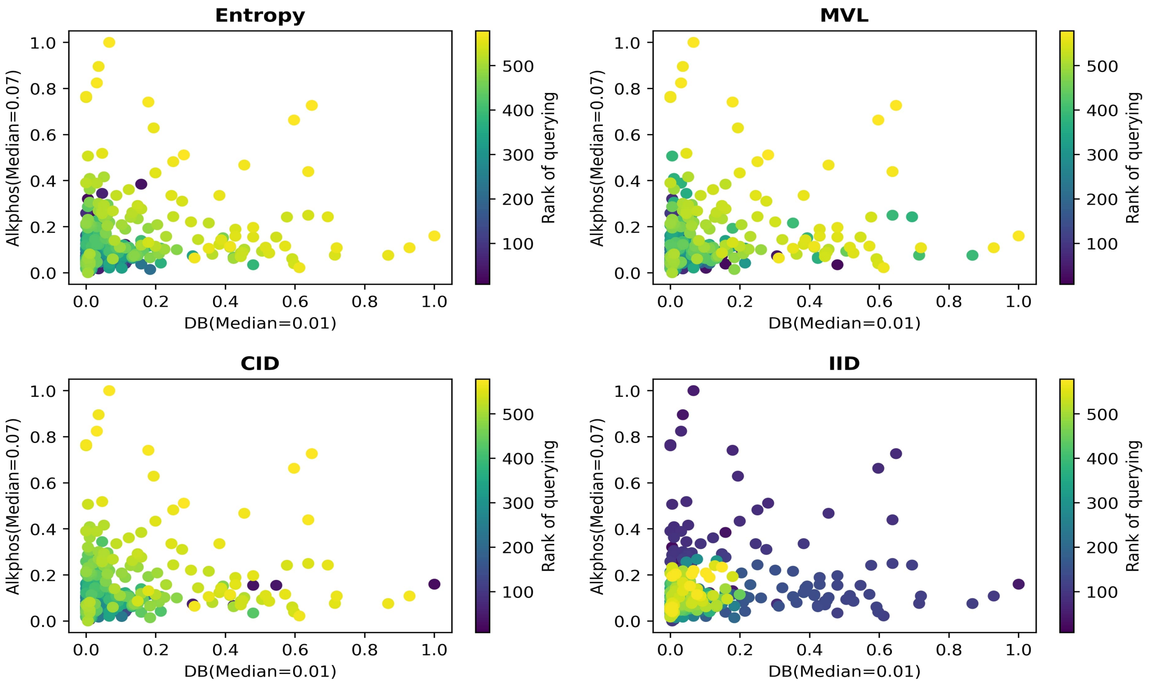

Figure S14: Querying order exhibited by AL algorithms in Indian liver dataset

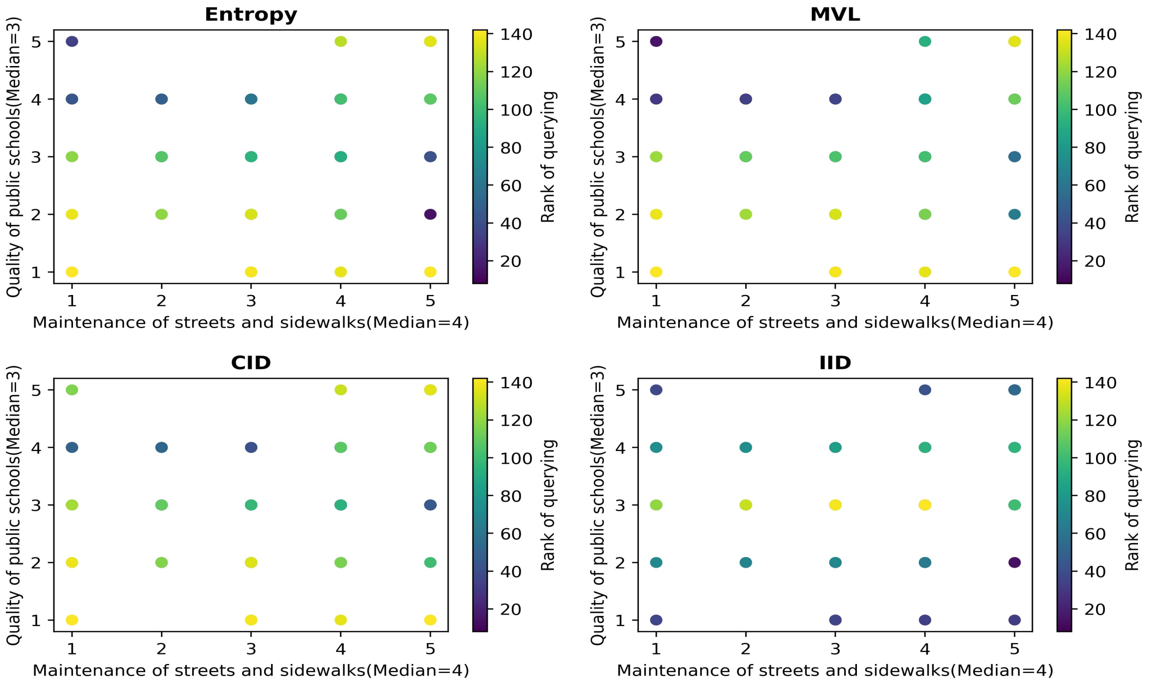

Figure S15: Querying order exhibited by AL algorithms in Happiness survey dataset

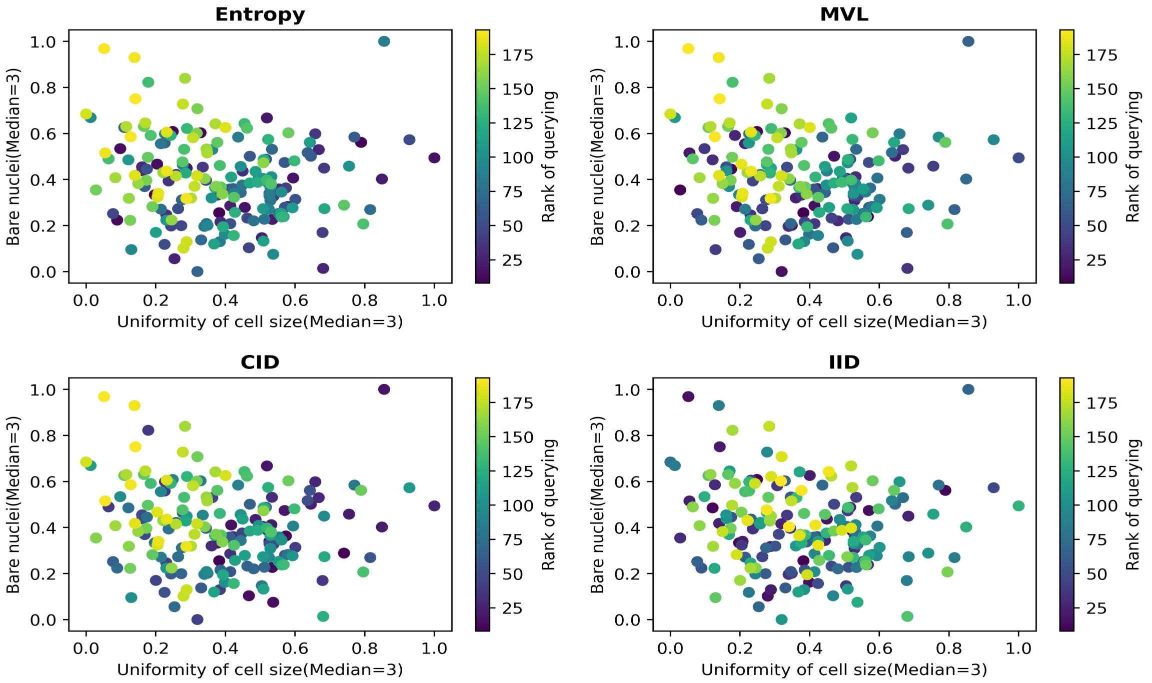

Figure S16: Querying order exhibited by AL algorithms in Breast cancer-prognostic dataset

3 SECTION C: PERFORMANCE OF AL ALGORITHMS WITH RF AND SVM CLASSIFIERS

Dataset	Labelling Strategy	Max. value	Random	Entropy	MVL	CID	Proposed IID
	Ground Truth	422	398.26	406.75	405.55	405.03	**407.31**
Car Condition	FFT	422	318.71	320.13	320.14	320.03	**320.17**
	Tallying	422	364.20	364.91	364.88	364.61	**364.95**
	Ground Truth	439	422.60	**427.58**	427.42	426.93	427.05
Breast Cancer	FFT	439	408.38	407.37	407.41	**407.47**	407.24
	Tallying	439	416.23	417.97	**418.00**	417.64	417.72
	Ground Truth	279	255.14	**259.75**	259.63	259.47	258.33
Wholesale Customer	FFT	279	247.93	**248.26**	**248.26**	**248.26**	248.21
	Tallying	279	201.41	201.35	201.36	201.19	**201.72**
	Ground Truth	852	736.53	**754.72**	754.36	754.61	753.56
Raisin	FFT	852	705.88	705.50	**705.51**	705.50	**705.51**
	Tallying	852	691.04	691.95	691.91	691.90	**691.96**
	Ground Truth	161	159.13	**160.77**	**160.77**	160.74	160.68
Wine	FFT	161	153.95	152.84	152.84	152.76	**153.05**
	Tallying	161	151.37	149.33	149.46	149.40	**149.47**
	Ground Truth	958	756.61	**781.87**	770.75	777.17	778.78
Maternal Health	FFT	958	656.40	657.43	**657.55**	657.04	656.87
	Tallying	958	599.89	600.03	**600.05**	**600.05**	600.00
	Ground Truth	231	216.86	224.35	223.83	223.22	**224.53**
Algerian Forest	FFT	231	198.94	**200.35**	200.33	200.22	200.29
	Tallying	231	193.12	193.96	193.87	193.89	**194.05**
	Ground Truth	1222	885.84	**914.64**	913.28	906.19	913.78
Contraceptive	FFT	1222	710.62	711.81	**711.82**	711.78	**711.82**
	Tallying	1222	756.31	**756.51**	756.50	756.50	756.36
	Ground Truth	55	53.89	**54.57**	54.52	54.40	54.55
Echocardiogram	FFT	55	52.06	52.09	**52.12**	52.11	51.83
	Tallying	55	44.33	44.04	**44.06**	43.91	44.03
	Ground Truth	143	142.47	**142.96**	**142.96**	**142.96**	142.95
Chronic Kidney Disease	FFT	143	**142.96**	**142.96**	**142.96**	142.95	142.95
	Tallying	143	141.70	**142.07**	**142.07**	**142.07**	**142.07**

Cervical Cancer	Ground Truth	65	61.14	**63.33**	63.24	62.59	63.15
	FFT	65	56.07	56.35	**56.45**	56.12	56.23
	Tallying	65	56.54	58.10	**58.32**	57.96	58.13
Parkinsons Disease	Ground Truth	186	172.75	180.39	**180.41**	178.91	179.58
	FFT	186	145.51	**145.75**	**145.74**	145.48	145.69
	Tallying	186	132.18	132.43	**132.47**	132.50	132.30
Indian Liver Patient	Ground Truth	568	446.12	464.72	**465.23**	457.44	464.90
	FFT	568	384.03	**384.68**	384.58	357.64	384.57
	Tallying	568	**376.75**	376.31	376.04	376.45	376.13
Happiness Survey	Ground Truth	135	100.10	102.16	**102.71**	100.56	102.22
	FFT	135	84.73	85.40	**85.41**	85.33	85.21
	Tallying	135	89.23	**89.34**	**89.34**	89.31	89.30
Breast Cancer-Prognostic	Ground Truth	185	160.25	164.48	**164.67**	161.02	163.26
	FFT	185	135.55	138.79	139.26	137.93	**140.50**
	Tallying	185	113.12	115.19	115.65	113.83	**118.03**

Table S1: Reported is the area under the Learning curve for each query approach(Maximum value is the total number of data points queried) when RF classifier was used. The cases where they perform worse than random are Underlined

Dataset	Labelling Strategy	Max. value	Random	Entropy	MVL	CID	Proposed IID
Car Condition	Ground Truth	422	388.41	**398.69**	398.19	395.04	398.56
	FFT	422	319.38	320.34	**320.89**	320.18	320.44
	Tallying	422	360.46	361.84	362.08	357.03	**362.46**
Breast Cancer	Ground Truth	439	420.53	420.87	421.29	421.02	**421.35**
	FFT	439	414.73	**413.30**	412.98	412.83	410.40
	Tallying	439	420.19	**421.26**	420.95	420.82	420.21
Wholesale Customer	Ground Truth	279	252.81	**256.63**	256.60	256.19	256.00
	FFT	279	246.09	247.33	247.46	246.94	**248.02**
	Tallying	279	203.46	201.99	203.47	200.47	**204.58**
Raisin	Ground Truth	852	715.68	718.33	**718.68**	718.02	717.02
	FFT	852	708.00	**708.59**	708.38	708.56	708.50
	Tallying	852	689.41	687.84	687.44	**688.08**	686.93
Wine	Ground Truth	161	160.49	**160.95**	159.17	**160.95**	160.93
	FFT	161	155.96	**156.21**	152.96	156.05	**156.21**

	Tallying	161	155.48	155.93	152.72	**155.97**	155.85
Maternal Health	Ground Truth	958	718.76	725.71	675.00	**726.04**	725.02
	FFT	958	658.92	**669.48**	647.11	666.16	664.50
	Tallying	958	618.27	615.93	597.44	614.56	**620.46**
Algerian Forest	Ground Truth	231	188.77	194.27	**195.35**	193.05	193.04
	FFT	231	189.45	192.14	**192.17**	189.45	190.75
	Tallying	231	181.16	184.90	184.46	182.97	**185.50**
Contraceptive	Ground Truth	1222	807.94	825.16	**829.84**	828.85	825.63
	FFT	1222	690.12	695.23	694.35	691.82	**695.51**
	Tallying	1222	717.77	738.19	732.99	735.63	**747.48**
Echocardiogram	Ground Truth	55	51.70	51.72	**51.96**	51.48	51.80
	FFT	55	51.54	51.18	**51.32**	50.97	51.29
	Tallying	55	44.43	42.98	43.24	43.01	**43.84**
Chronic Kidney Disease	Ground Truth	143	142.88	**142.99**	**142.99**	**142.99**	**142.99**
	FFT	143	142.88	**142.99**	**142.99**	**142.99**	**142.99**
	Tallying	143	142.14	**142.96**	**142.96**	**142.96**	142.85
Cervical Cancer	Ground Truth	65	59.15	60.45	**60.79**	60.71	60.54
	FFT	65	56.05	56.14	**56.70**	56.07	56.06
	Tallying	65	57.57	59.19	**59.50**	59.14	59.29
Parkinsons Disease	Ground Truth	186	160.15	164.91	**165.38**	164.00	164.04
	FFT	186	144.06	144.49	**145.30**	143.92	144.62
	Tallying	186	130.63	**129.63**	**129.63**	129.54	129.59
Indian Liver Patient	Ground Truth	568	405.82	**405.94**	405.90	405.54	405.96
	FFT	568	369.72	349.37	357.72	341.65	**363.60**
	Tallying	568	345.37	342.21	344.36	334.93	**353.82**
Happiness Survey	Ground Truth	135	89.97	89.86	**91.66**	90.32	88.50
	FFT	135	82.78	83.55	**84.03**	82.36	83.61
	Tallying	135	87.55	88.39	**88.52**	88.06	86.87
Breast Cancer-Prognostic	Ground Truth	185	148.04	150.51	**149.95**	148.76	149.50
	FFT	185	134.87	138.53	138.69	**138.72**	137.82
	Tallying	185	109.81	114.20	**115.93**	109.68	115.40

Table S2: Reported is the area under the Learning curve for each query approach(Maximum value is the total number of data points queried) when SVM classifier was used. The cases where they perform worse than random are Underlined
